# Early-Stage Screening of Piperonylic Acid Analogues and Their Salts as Synergists for the Class of Pyrethroid Insecticides

**DOI:** 10.1021/acsomega.6c05301

**Published:** 2026-08-26

**Authors:** Ramana Pydi, Alexander Wolf, Jennifer Bernard, Christoph Zutz, Birgit Bromberger, Anita Sosnowska, Szymon Zdybel, Tomasz Puzyn, Rolf Breinbauer, Patrick-Julian Mikuni-Mester

**Affiliations:** † Centre for Food Science and Veterinary Public Health, Clinical Department for Farm Animals and Food System Science, 27260 University of Veterinary Medicine Vienna, Veterinaerplatz 1 Vienna 1210, Austria; ‡ Institute of Organic Chemistry, 27253 Graz University of Technology, Stremayrgasse 9, Graz 8010, Austria; § 474572 Kwizda Agro GmbH, Universitätsring 6, Vienna 1010, Austria; ∥ 49646 QSAR Lab, Grunwaldzka Ave, 103A, Gdansk 80-244, Poland; ⊥ University of Gdansk, Faculty of Chemistry, Wita Stwosza 63, Gdansk 80-308, Poland

## Abstract

This study presents an early-stage screening of piperonylic acid analogues and their salts as synergists for the class of pyrethroid insecticides. Using the carbonate-based ionic liquid synthesis (CBILs) method, we synthesized 32 potential synergists from 4 piperonylic acid analogues in a one-step process and experimentally determined general toxicity parameters and synergistic activity in comparison with the commercial synergist piperonyl butoxide (PBO). *In vitro* assays revealed structure-dependent activity of synergists on detoxification enzymes of *Blattella germanica*, with several candidates demonstrating similar or even higher activity compared to PBO. Subsequent *in vivo* testing of selected compounds confirmed their overall synergistic potential but showed lower activity than PBO, underscoring the need to validate *in vitro* findings *in vivo*. The reported synergistic activity of piperonylic acid analogues and the ability for noncovalent modification can act as a starting point for future structure optimization to find alternatives for PBO in pyrethroid insecticide formulations.

## Graphical abstract

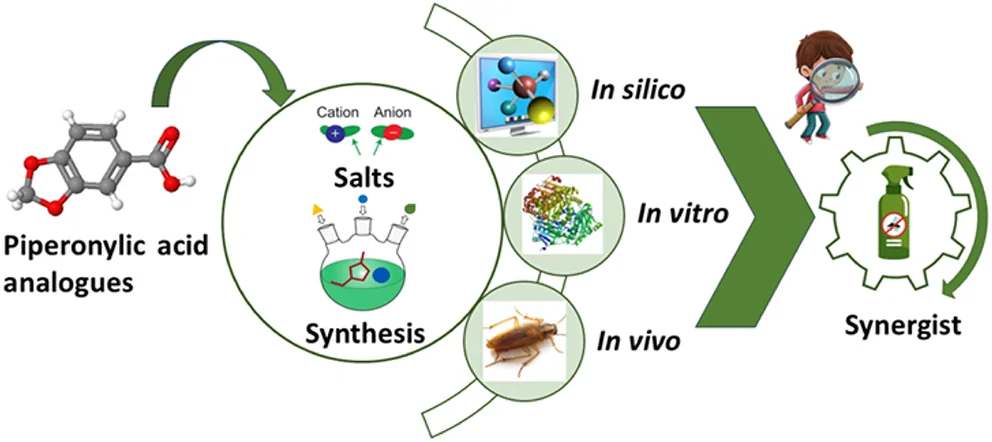

## Introduction

Agriculture is crucial for many national economies, yet biotic stresses, especially weeds and insects, cause major crop losses, accounting for 33% and 26% annual loss, respectively. With the global population projected to reach 9.8 billion by 2050, climate-driven pest outbreaks are expected to further increase losses, threatening food security in vulnerable regions.^1^ Pesticides and insecticides are widely used in agriculture to control insects, pests, and weeds and remain essential to modern farming and public health. Their global demand continues to rise, driven largely by persistent pest pressure and resistance development, which cause severe crop damage and major economic losses for farmers.^2^

Among the most widely utilized insecticides are synthetic pyrethroid compounds modeled after natural pyrethrins extracted from chrysanthemum flowers. Pyrethroids are known for their high potency, rapid knockdown capability, and relatively low toxicity to humans. However, their widespread and repeated application has led to the development of resistance in many insect pest populations.^3–10^ This growing resistance not only reduces the efficacy of control measures but also necessitates increased dosage and application frequency, thereby elevating costs and the environmental impact. A major factor contributing to pyrethroid resistance is metabolic detoxification, whereby insects enzymatically break down insecticides before they can exert their toxic effects. This process involves a range of detoxification enzymes, notably carboxylesterases, glutathione *S*-transferases, and cytochrome P450 monooxygenases (P450s).^11–13^ To counteract these resistance mechanisms and increase the effectiveness of insecticides, the use of chemical synergists has become an important complementary strategy, and in Figure 1, the currently most relevant commercial synergists are depicted.^14,15^ Although synergists have little or no insecticidal activity on their own, they increase insecticide effectivity, thus improving pest control while also allowing for lower insecticide doses.^16,17^ Synergists are especially useful in managing resistant pest species, and their application contributes to more efficient and sustainable pest control practices.^18–21^

**Figure 1:**
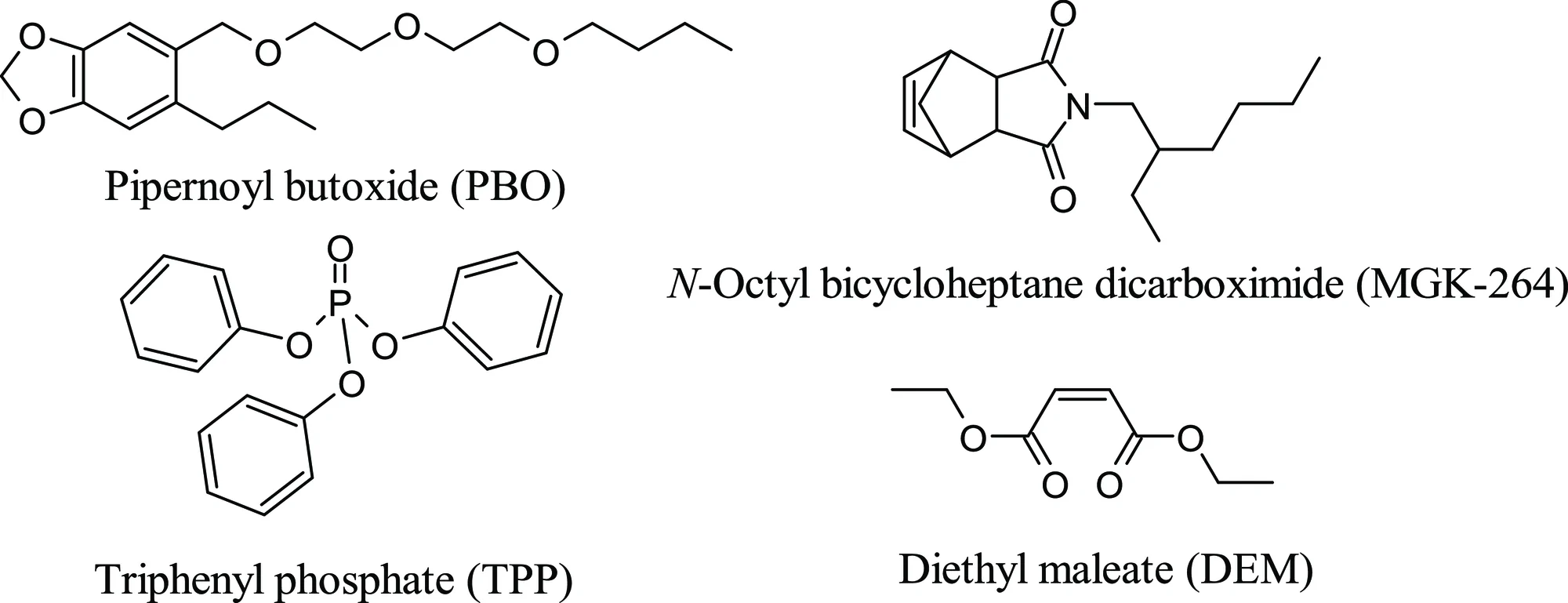
Common chemical synergists used in insecticide formulations.

Piperonyl butoxide (PBO) is the most widely used synergist in commercial insecticide formulations based on pyrethroids.^22,23^ Its primary role is the inhibition of detoxification enzymes, which are responsible for the metabolic degradation of pyrethroids.^22–25^ By suppressing these enzymes, PBO increases the susceptibility of resistant insects, such as*Blattella germanica* (German cockroach), to pyrethroid application.^26^ Nevertheless, PBO is not without limitations, including issues related to its environmental persistence, toxicity, and suspected carcinogenicity, which have prompted efforts to identify improved alternatives.^27^

In response to these challenges, recent studies have focused on the development of structural analogues of PBO with enhanced synergistic properties.^28–32^ These novel compounds aim to deliver more effective and selective inhibition of resistance-associated enzymes; however, so far, they have not reached the market. The continued innovation and application of such synergists hold significant promise for overcoming insecticide resistance and reinforcing less impact on the environment by chemically based pest management systems. Secondary plant metabolites, such as alkaloids, saponins, phenols, and terpenes, show significant potential as ecological alternatives for controlling insect pests because of their bioactive properties.^33,34^ While numerous studies have focused on the direct application of these compounds as ecological insecticides, their synergistic potential remains insufficiently investigated. Investigating these metabolites as synergists could facilitate the use of lower insecticide doses, thereby reducing the environmental impact and enhancing insecticide efficacy. Our research focused on studying plant metabolites such as piperonylic acid (PA), 3,4-(methylenedioxy)­phenylacetic acid (3,4-MPA), 3,4-(methylenedioxy)­cinnamic acid (3,4-MCA), and 3,4-dimethoxycinnamic acid (3,4-DCA), as these compounds all pertain to the methylenedioxyphenyl ring and exhibit structural similarities to piperonyl butoxide. Although piperonylic acid analogues have been studied as enzyme inhibitors,^35,36^ their potential as synergists for insecticides has not been explored. Our objective was to investigate their potential as synergists by assessing their inhibitory effects on detoxification enzymes and their efficacy in insecticide formulations.

Our inspiration to design potential synergists comes from the concept of Active Pharmaceutical Ingredient–Ionic Liquids (API-ILs). Developing salts of target active compounds is a well-established, noncovalent strategy that enables efficient new formulations. In this approach, the biological properties of an active molecule can be tuned by selecting an appropriate counterion rather than by covalent modification,^37^ utilizing the CBILs method.^38^ To modulate the efficacy of piperonylic acid analogues, we employed CBILs to synthesize 32 novel piperonylic acid analogue salts, chosen because of their clean, one-pot reaction conditions, efficiency in generating structurally distinct salts in pure form, and halide-free synthesis process.^38,39^ These have demonstrated applications in the functionalization of naturally occurring precursors such as fatty acids^40^ and lignin derivatives,^41^ thereby equipping them to modulate properties.

All 32 synthesized salts, along with 4 piperonylic acid analogues, were evaluated through a combination of experimental and computational methods, including antimicrobial activity, biodegradability, preliminary *in vitro* screening, and *in vivo* synergistic activity. Furthermore, the synergistic activity observed in the present study is demonstrated on*B. germanica*, a widely recognized insect pest and a well-established model for evaluating insecticide resistance. Its documented resistance to pyrethroid insecticides makes it particularly suitable for assessing efficacy.^5,26,42^

## Results and Discussion

### Synthesis

We implemented the carbonate-based ionic liquid synthesis (CBILs)^38^ method to synthesize 32 distinct salts through a clean, one-pot reaction (Scheme 1) utilizing 8 structurally different cations and 4 piperonylic acid analogues (Figure 2 and Table S1). The transformation proceeded quantitatively, achieving complete conversion and quantitative yields without the need for additional reagents or purification steps. The desired salts were obtained simply by the evaporation of the volatile alcohol formed during the reaction. This solvent-efficient, high-atom-economy approach aligns with a streamlined approach with higher yields and the ability to obtain structurally diverse salts in pure form. Structures of all 32 synthesized salts were confirmed by NMR (Figures S2 and S7).

**Scheme 1:**
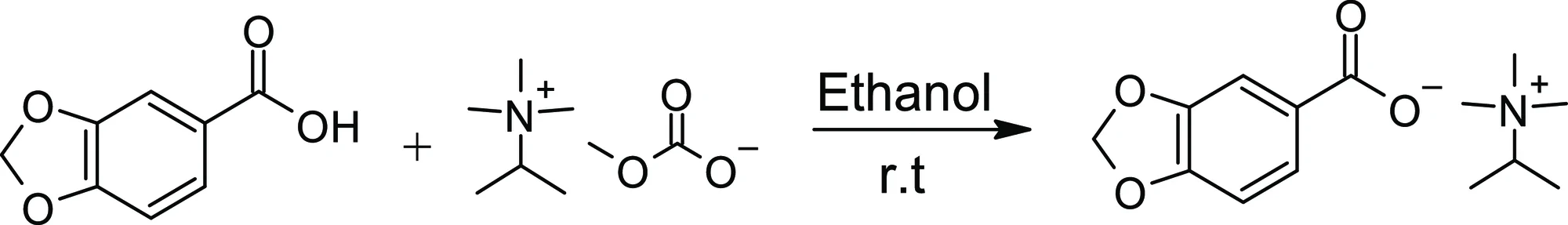
General Concept of the Carbonate Based Ionic Liquid Synthesis (CBILs) Route

**Figure 2:**
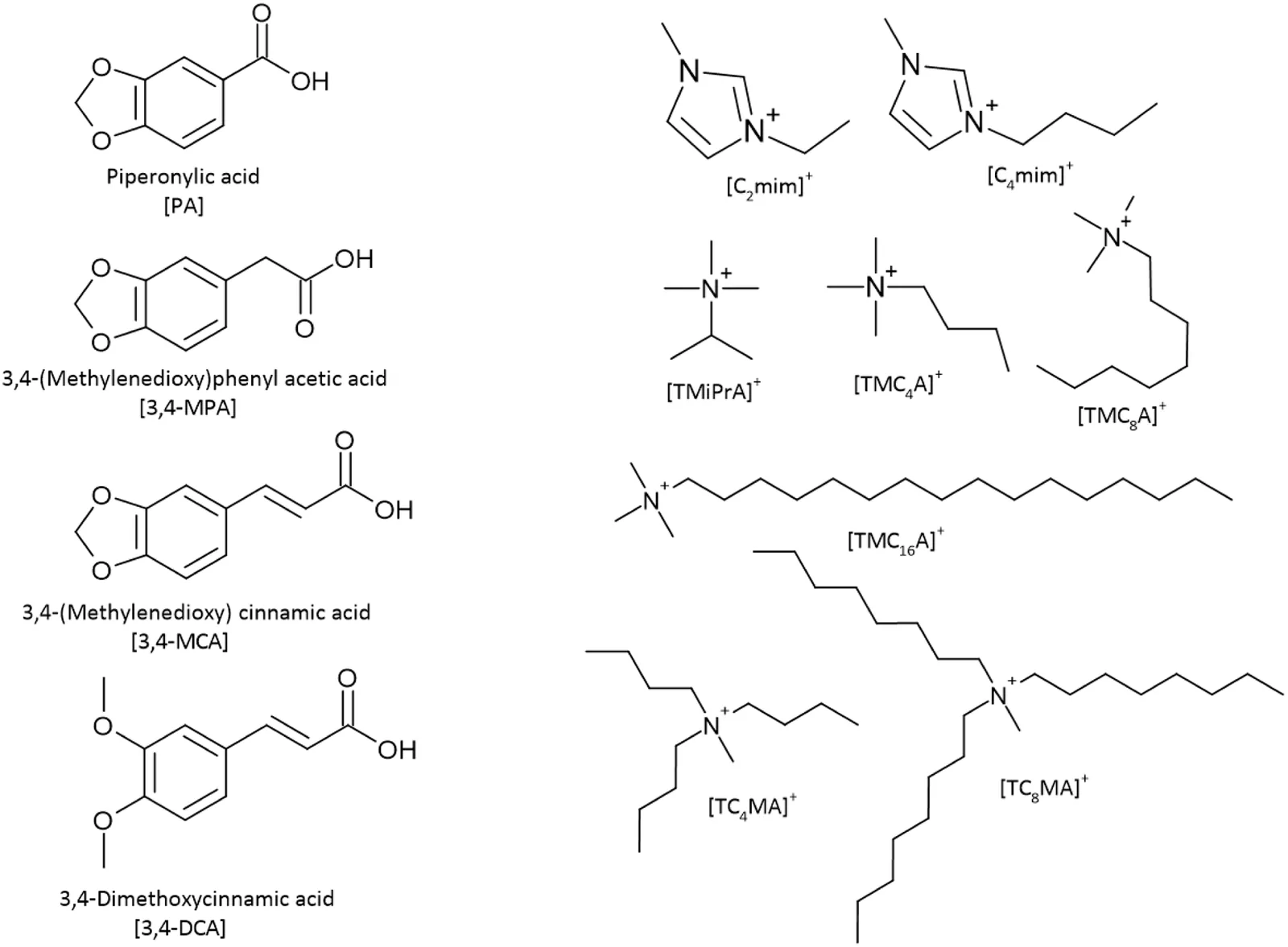
Piperonylic acid analogues and cations used to obtain salts in this study.

### Antimicrobial Activity, qPCR-Based Enzyme Inhibition Assay, and Biodegradation of Salts

Antimicrobial activity, qPCR-based enzyme inhibition, and biodegradation can provide preliminary indications of the compatibility of potential synergists with the environment and an initial toxicity evaluation in order to exclude the most problematic compounds for subsequent testing. All 32 novel salts were evaluated by using a combination of experimental and computational methods. For toxicity indication, antimicrobial activity and enzyme inhibition activity were experimentally determined following a previously published approach.^40,43^ Due to short generation times and existing screening methods, bacterial species are commonly used for toxicity studies.^44^ To examine antimicrobial activity, the minimal inhibitory concentration (MIC), the lowest concentration inhibiting bacterial growth, was determined on two different bacterial species, Gram-positive*L. monocytogenes* and Gram-negative*E. coli* (Table S3). The classification criteria were applied according to the approach from Pasino and Smith;^45^ salts exhibiting MIC values above 1 mg/mL were classified as harmless, those with MIC values between 0.1 and 1 mg/mL as practically harmless, MIC values between 0.01 and 0.1 mg/mL as slightly toxic, and MIC values below 0.01 mg/mL as moderately toxic.

The antimicrobial activity tests of all compounds revealed no significant differences between the two species and are therefore discussed together (see Supporting Information, Table S3 for detailed results). As per toxicity assessment (Table 1), 20 salts containing imidazolium cations with short alkyl chains such as [C_2_mim] and [C_4_mim] and ammonium cations with short alkyl chains such as [TMiPrA], and [TMC_4_A], [TC_4_MA] were considered harmless (MIC > 1 mg/mL), 4 salts containing [TMC_8_A] cation were practically harmless (MIC 0.1 to 1 mg/mL), while 4 salts containing [TC_8_MA] cation exhibited slight toxicity (MIC 0.01 to 0.1 mg/mL), and 4 salts containing [TMC_16_A] cation showed moderate toxicity (MIC < 0.01 mg/mL). The results demonstrate that the overall toxicity of salts followed the well-known cationic side-chain effect, with the length or number of elongated alkyl chains increasing overall toxicity,^40,43^ while the respective anion demonstrated no significant impact. These findings were confirmed by the results of the general enzyme inhibition assay, where the lowest concentrations leading to complete PCR inhibition were determined (Tables 1 and S4).^43^ By adapting the same toxicity classification of antimicrobial activity tests, it is followed in the qPCR-based enzyme inhibition assay that 12 salts were harmless (>1 mg/mL), 15 salts practically harmless (0.1 to 1 mg/mL), 4 salts containing the [TMC_16_A] cation exhibited slight toxicity (0.01 to 0.1 mg/mL), and 1 salt containing [TC_8_MA] showed moderate toxicity (Table 1).

**Table 1:** Toxicity and Predicted Biodegradation of Piperonylic Acid Analogue Salts

		^a^ **Toxicity evaluation**		
**Salt Nr.**	**Salts**	**Bacteria**	**PCR enzyme**	^b^ **Biodegradation Classification**
**1**	[C_2_mim][POAc]	harmless	harmless	not easily biodegradable
**2**	[C_4_mim][POAc]	harmless	harmless	not easily biodegradable
**3**	[TMiPrA][POAc]	harmless	practically harmless	easily biodegradable
**4**	[TMC_4_A][POAc]	harmless	practically harmless	easily biodegradable
**5**	[TMC_8_A][POAc]	practically harmless	practically harmless	easily biodegradable
**6**	[TMC_16_A][POAc]	moderately toxic	slightly toxic	easily biodegradable
**7**	[TC_4_MA][POAc]	harmless	practically harmless	easily biodegradable
**8**	[TC_8_MA][POAc]	slightly toxic	practically harmless	easily biodegradable
**9**	[C_2_mim][3,4-MPOAc]	harmless	practically harmless	not easily biodegradable
**10**	[C_4_mim][3,4-MPOAc]	harmless	harmless	not easily biodegradable
**11**	[TMiPrA][3,4-MPOAc]	harmless	harmless	easily biodegradable
**12**	[TMC_4_A][3,4-MPOAc]	harmless	practically harmless	easily biodegradable
**13**	[TMC_8_A][3,4-MPOAc]	practically harmless	harmless	easily biodegradable
**14**	[TMC_16_A][3,4-MPOAc]	slightly toxic	slightly toxic	easily biodegradable
**15**	[TC_4_MA][3,4-MPOAc]	harmless	harmless	easily biodegradable
**16**	[TC_8_MA][3,4-MPOAc]	slightly toxic	moderately toxic	easily biodegradable
**17**	[C_2_mim][3,4-MCOAc]	harmless	practically harmless	not easily biodegradable
**18**	[C_4_mim][3,4-MCOAc]	harmless	practically harmless	not easily biodegradable
**19**	[TMiPrA][3,4-MCOAc]	harmless	practically harmless	easily biodegradable
**20**	[TMC_4_A][3,4-MCOAc]	harmless	practically harmless	easily biodegradable
**21**	[TMC_8_A][3,4-MCOAc]	practically harmless	practically harmless	easily biodegradable
**22**	[TMC_16_A][3,4-MCOAc]	moderately toxic	slightly toxic	easily biodegradable
**23**	[TC_4_MA][3,4-MCOAc]	harmless	harmless	easily biodegradable
**24**	[TC_8_MA][3,4-MCOAc]	moderately toxic	practically harmless	easily biodegradable
**25**	[C_2_mim][3,4-DCOAc]	harmless	practically harmless	not easily biodegradable
**26**	[C_4_mim][3,4-DCOAc]	harmless	harmless	not easily biodegradable
**27**	[TMiPrA][3,4-DCOAc]	harmless	harmless	easily biodegradable
**28**	[TMC_4_A][3,4-DCOAc]	harmless	harmless	easily biodegradable
**29**	[TMC_8_A][3,4-DCOAc]	practically harmless	harmless	easily biodegradable
**30**	[TMC_16_A][3,4-DCOAc]	moderately toxic	slightly toxic	easily biodegradable
**31**	[TC_4_MA][3,4-DCOAc]	harmless	harmless	easily biodegradable
**32**	[TC_8_MA][3,4-DCOAc]	slightly toxic	practically harmless	easily biodegradable

a Classification for toxicity assessment: >1 mg/mL concentration is harmless; 0.1 to 1 mg/mL concentration is practically harmless; 0.01 to 0.1 mg/mL concentration is slightly toxic; and <0.01 mg/mL concentration is moderately toxic.

b Biodegradation classification as per AquaBoxIL.

Although toxicity evaluation was limited to antimicrobial activity assays and qPCR-based enzyme inhibition responses, these data indicate concerns regarding environmental compatibility for compounds with longer alkyl chains. In addition to the experimental toxicity evaluation, we utilized quantitative structure–activity/property relationship models (QSAR/QSPR) for predicting the biodegradability of novel compounds using the AquaBoxIL tool.^46^ The predicted end point corresponds to the percentage of biodegradation following the OECD 310 protocol. The analysis of the estimated values revealed that imidazolium-based cations exhibit lower biodegradability and should be avoided, corroborating previous findings,^47^ whereas quaternary ammonium salts demonstrated greater biodegradability (Tables 1 and Table S5). Overall, a decrease in the biodegradability of salts appears to be primarily associated with the presence of aromatic functional groups in cations, while the nature of the anion had negligible influence, and these findings could serve as a preliminary screening to understand environmental safety and synergistic compatibility. Nevertheless, the data presented in this study have to be considered an initial screening to exclude problematic and potentially toxic compounds from subsequent experiments. In the case of more promising candidates with a chance for real-world application, further characterization is required, including a broader toxicity evaluation, environmental persistence, bioaccumulation potential, and standardized OECD toxicity end points.^48,49^

### *In Vitro* Preliminary Synergistic Activity Screening of Piperonylic Acid Analogues and Salts

To evaluate the preliminary potential activity of synergists for insecticide formulations, 4 piperonylic acid analogues and all 32 salts were evaluated in a preliminary *in vitro* screening for inhibition of detoxification enzymes from *B. germanica*. Specifically, glutathione-*S*-transferase (GST) and esterase (EST) were tested, as they play crucial roles in insect defense against pyrethroid insecticides.^5,11^ A commercial synergist (PBO) was included for comparison. The inhibitory effects of salts on GST and EST enzymes were assessed by determining EC_50_ values for both enzymes (Figure 3; Table S6).

**Figure 3:**
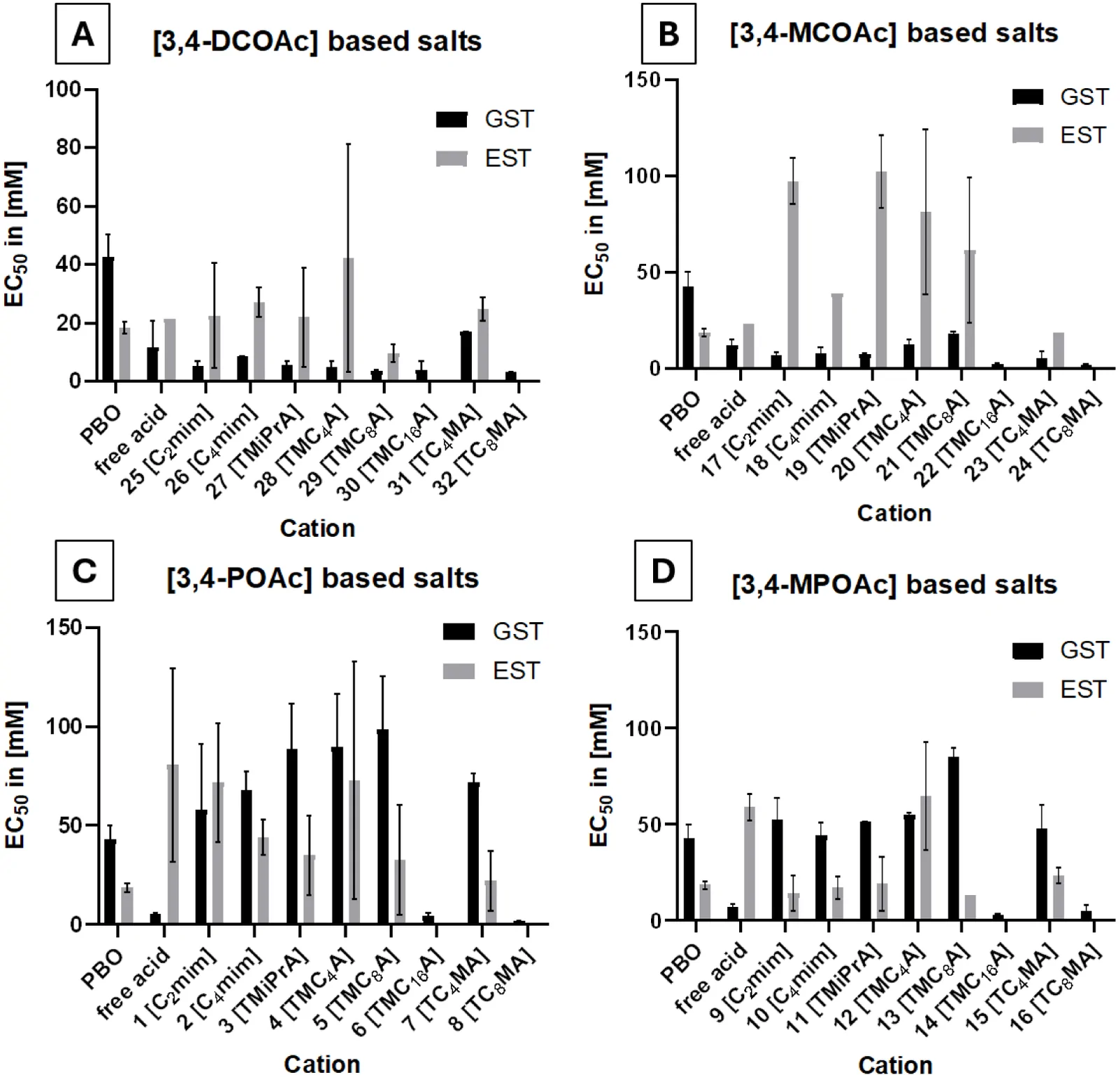
EC_50_ values for salts in GST and EST enzyme inhibition assays in comparison to PBO; results are presented as mean values in millimolar (mM) concentrations, including standard deviations obtained from two biological and technical replicates. **A.** [3,4-DCOAc]-based salts, **B.** [3,4-MCOAc]-based salts, **C.** [POAc]-based salts, **D.** [3,4-MPOAc]-based salts.

As expected, the commercial synergist PBO showed inhibition toward both detoxification enzymes (GST: EC_50_ = 42.8 mM, EST: EC_50_ = 18.5 mM) and therefore provides a benchmark for evaluating the relative inhibitory effects of the salts. 3,4-Dimethoxycinnamic acid (3,4-DCA) proved to be an effective inhibitor of both detoxification enzymes. Inhibition of EST (EC_50_ = 21.5 mM) was similar to that of PBO, while for GST (EC_50_ = 11.75 mM) the activity was higher (Figure 3A and Table S6). The inhibitory effects of 3,4-DCOAc-based salts showed enhanced activity against GST, and in a few cases, of enhanced inhibitory effects against EST compared to the commercial synergist PBO. Almost all of the 3,4-DCOAc-based salts inhibited GST enzyme in the lower millimolar range, with **29, 30**, and **32** displaying the strongest activity (EC_50_ = 3.5 mM, 3.9 mM, and 3.2 mM, respectively). These values represent more than a 10-fold increase in potency compared to PBO. However, **30** and **32** containing long alkyl-chain cations such as TMC_8_A also displayed a general enzyme inhibition capacity (Table 1), and therefore an unspecific inhibition is most likely. Salts **25–28** also demonstrated higher inhibitory activities compared to PBO while being predicted to have low toxicity in the toxicity tests, and **31** showed similar efficacy as free acid. For EST enzyme inhibition, the 3,4-DCOAc-based salts displayed wider variability but showed similar activity patterns compared to the free acid. Notably, **30** and **32** were the most potent EST inhibitors in this series (EC_50_ = 0.4 mM), with similar performance in the GST assay results. **29** also demonstrated balanced inhibition of both enzymes, with EC_50_ values of 3.5 mM (GST) and 9.6 mM (EST).

3,4-(Methylenedioxy)­cinnamic acid (3,4-MCA) also proved to be an effective inhibitor of both detoxification enzymes. Inhibition of EST (EC_50_ = 23.2 mM) was similar to that of PBO, while for GST (EC_50_ = 11.8 mM) the activity was higher compared to that of PBO (Figure 3B and Table S6). Overall, the 3,4-MCA inhibition potential is similar to that of 3,4-DCA, indicating no effect of their structural difference. The 3,4-MCOAc-based salts (Figure 3B) also showed inhibitory effects on GST similar to the 3,4-DCOAc-based salts, while for EST, a reduced activity was observed. Compared to the free acid, **22** and **24** demonstrated high activity against both enzymes, but as they also showed high activity in general toxicity (Table 1), unspecific effects are most likely. One interesting effect was observed for **19**, as it demonstrated strong inhibition of GST but was the weakest inhibitor of EST (EC_50_ = 102.4 mM) among all 32 salts and 4 piperonylic acid analogues.

Piperonylic acid (PA) exhibited high inhibition activity on GST (EC_50_ = 5.4 mM) compared to PBO, being the most effective GST inhibitor among all 4 piperonylic acid analogues, while for EST (EC_50_ = 80.5 mM) the activity was lower compared to PBO (Figure 3C, Table S6). Surprisingly, the inhibition potency of POAc-based salts was drastically reduced on GST, which was not observed for either 3,4-DCOAc or 3,4-MCOAc-based salts. The only exceptions were **6** and **8**, which is in alignment with their general toxicity in toxicity tests (Table 1).

Similar to PA, 3,4-(methylenedioxy)­phenylacetic acid (3,4-MPA) also demonstrated high inhibition activity on GST (EC_50_ = 7 mM) but lower activity against EST (EC_50_ = 59 mM) compared to PBO (Figure 3D, Table S6). As with the POAc-based salts, the 3,4-MPOAc-based salts demonstrated drastically lower inhibition of GST in comparison to the free acid, while for EST the activity was higher for most salts, with the exception of **12**, which exhibited low inhibitory potency.

Overall, *in vitro* analysis revealed that all 4 piperonylic acid analogues are indeed possible potential synergists for insecticides, as they demonstrated strong inhibition potency on GST and EST, similar to that of PBO. In the case of 3,4-DCA and 3,4-MCA, the observed inhibition profiles indicate that structural modifications, such as the opening of the piperonyl ring in 3,4-DCA, do not significantly influence GST inhibition. In contrast, this structural change affected EST inhibition, where a loss of activity was observed, suggesting that the intact piperonyl ring plays an important role in conferring inhibitory potency, particularly toward EST. For PA and 3,4-MPA, contrasting inhibition profiles were observed for both GST and EST, and although these compounds are structurally very similar, they exhibited distinct modulation of enzyme activity, highlighting the effect of structural variations on inhibition.

Salts containing cations with long alkyl chains exhibited strong inhibition of both GST and EST and showed a broad inhibition capacity (Table 1), indicating that the structural features of the countercation significantly influence inhibitory potential. As these salts also demonstrated elevated toxicity (Table 1), their applicability is limited. The experimental design for preliminary phenotypic screening for potential synergists was intended to be as close as possible to the target organism, and therefore, *in vitro* assays were performed on crude enzyme extracts from*Blattella germanica* homogenates. Overall, the enzyme inhibition results indicate possible structure–activity relationship (SAR) trends, demonstrating that careful structural tuning, particularly through variation of the cationic component, could improve the synergistic activity of piperonylic acid analogues and their corresponding salts. In order to obtain more accurate kinetic parameters and a mechanistic interpretation, future studies should aim to utilize purified enzymes as well as perform molecular docking analyses for elucidating ligand–enzyme interactions and validating the mechanistic basis of the observed synergistic effects, which were not within the scope of this study.^50–52^

### *In Vivo* Evaluation of Piperonylic Acid Analogues and Salts as Potential Synergists

Based on the predicted toxicity and *in vitro* activity evaluations, it became clear that salts containing either elongated or multiple alkyl chains in the cation demonstrate considerable toxicity themselves, thus not being applicable as potential synergists. Therefore, for *in vivo* experiments, we focused on three structurally different cations ([C_2_mim], [TMiPrA], and [TMC_4_A]) that demonstrated no toxicity themselves, thus enabling evaluation of their applicability as potential synergists. In total, 12 salts and 4 piperonylic acid analogues were tested as potential synergists in combination with pyrethrum as the active ingredient against*Blattella germanica*, which is a well-established model insect for evaluating resistance to pyrethroid insecticides. Figure 4 shows the effects of both pyrethrum alone and in combination with the commercial synergist PBO. No knockdown effect or mortality of either DMSO or ethanol, which were used as solvents in the experiments, was observed (data not shown). While having an initial knockdown effect on the insects (60% of affected individuals), pyrethrum alone does not lead to a significant mortality rate (20%) after 48 h. This observable recovery is due to the aforementioned detoxification enzymes. In contrast, in combination with PBO, the mortality increased noticeably (yielding 60% mortality), indicating that metabolic detoxification pathways were suppressed and that the insects were more susceptible to pyrethrum. These results confirm PBO’s well-established role in enhancing pyrethroid toxicity through effective inhibition of metabolic enzymes responsible for detoxification and serving as a benchmark synergist for *in vivo* evaluation of the novel synergists in this study.

**Figure 4:**
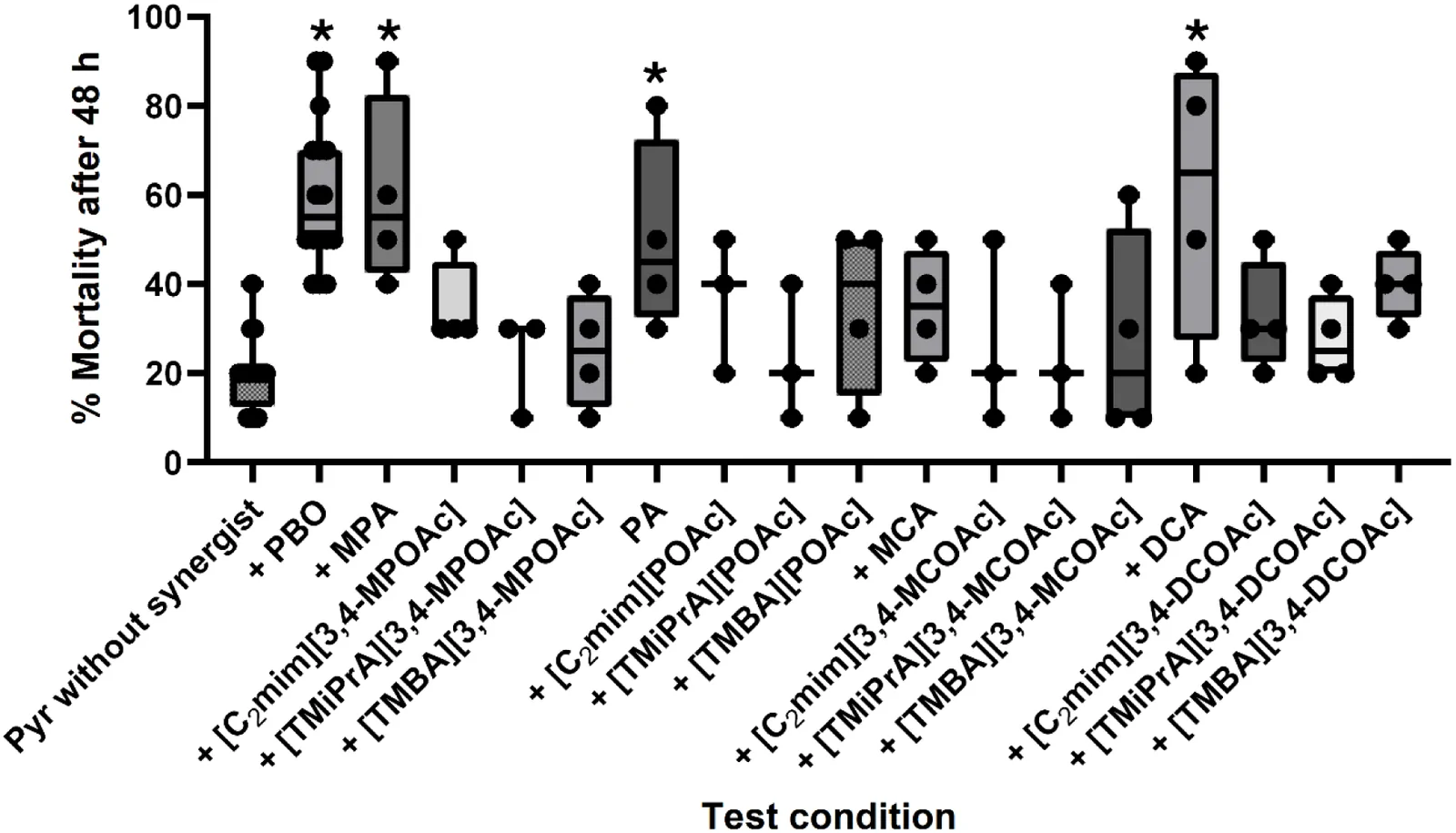
Synergist efficacy with pyrethrum (Pyr). 48-h mortality of *B. germanica* (% of 10 treated individuals per experiment) after exposure to pyrethrum (0.5 mg/mL in ethanol) alone or in combination with synergists. PBO was tested at 10 mg/mL in ethanol, while all salts and the corresponding free acids were tested at 50 mg/mL in DMSO. Asterisks indicate significant differences versus pyrethrum alone (* *p* < 0.05).

In initial *in vivo* tests, piperonylic acid analogues and salt concentrations similar to PBO (10 mg/mL), in combination with pyrethrum (0.5 mg/mL), were tested. However, at these concentrations, no synergistic effects of either the free acids or the corresponding PAILs were observed (data not shown). Therefore, 5-fold higher concentrations of all compounds were tested while the concentration of pyrethrum was kept constant (0.5 mg/mL). Figure 3 summarizes the respective 48-h mortality rates, while the respective knockdown rates (1 h) and all molar concentrations for each synergist are included in the Supporting Information (Table S7).

Under the tested conditions, for 3,4-dimethoxycinnamic acid (3,4-DCA), 3,4-(methylenedioxy) phenylacetic acid (3,4-MPA), and piperonylic acid (PA), significant synergistic effects were found, while for 3,4-(methylenedioxy)­cinnamic acid (3,4-MCA), elevated but not statistically significantly increased mortality in comparison to pyrethrum without a synergist were found (Figure 3). These results clearly demonstrate that piperonylic acid analogues have the potential to be used as synergists in pyrethrum-based insecticide formulations. Surprisingly, out of the 12 tested salts, none showed statistically significant synergistic activity compared to pyrethrum alone, and only 4 (**1, 4, 9**, and **28**) showed an increased mortality rate of at least 40% but also a clearly reduced synergistic activity in comparison to their corresponding free acids. These results were surprising, as 3,4-MCOAc- and 3,4-DCOAc-based salts demonstrated low *in vivo* activity while being the most effective *in vitro* inhibitors. Nevertheless, the results also experimentally confirmed, *in vitro* and *in vivo*, both the general synergistic activity of piperonylic acid analogues and the ability to modulate their activity by conversion into salts, while also indicating possible suboptimal penetration of salts *in vivo* and highlighting that transferability between *in vitro* and *in vivo* activity is limited. Possible explanations for the observed discrepancies include decreased penetration of the cuticle or restricted membrane permeability of the respective compounds, resulting in lower *in vivo* concentrations. Another possibility is that the observed activity originates solely from the dissociated anion, which cannot be ruled out in vivo. However, because the salts of the piperonylic acid analogues were tested at slightly lower molar concentrations than the corresponding free acids (SI, Table S6) due to the *in vivo* assay design, yet still showed clear synergistic activity, this effect is likely minor. Nonetheless, dissociation rates should be experimentally determined for further optimized compounds.^53^ Thus, the lack of correlation between *in vitro* enzyme inhibition and *in vivo* synergistic efficacy reflects the complexity of translating biochemical activity into biological activity performance and suggests that parameters influencing the permeability of the cuticle or membranes should be additionally assessed in phenotypic screenings.

Overall, although the piperonylic acid analogues and their salts showed synergy *in vivo and in vitro*, none matched the potency of PBO, and at this state, they have to be considered as starting points for further optimization.

## Materials and Methods

### Materials

Piperonylic acid (PA), 3,4-(methylenedioxy)­phenylacetic acid (3,4-MPA), 3,4-(methylenedioxy)­cinnamic acid (3,4-MCA), 3,4-dimethoxycinnamic acid (3,4-DCA), reduced l-glutathione (GSH), Fast Garnet dye, DMSO, and ethanol were purchased from Merck, Germany. All cations in the alkyl carbonate form were purchased from Proionic GmbH, Austria. The DNA dye EvaGreen was purchased from Jena Bioscience GmbH, Germany. Taq DNA polymerase was purchased from Biozym Scientific GmbH, Germany.

*Listeria monocytogenes* EDGe (ATCC BAA-679) was used as a model organism for Gram- positive bacteria. *Escherichia coli* was used as a model organism for Gram-negative bacteria. The bacteria were maintained at −80 °C and were part of the collection of bacterial strains at the Center for Food Science and Veterinary Public Health, University of Veterinary Medicine Vienna, Vienna, Austria. All bacterial strains were grown overnight in tryptone soya broth with 0.6% (w/v) yeast extract (TSB-Y) at the respective growth temperatures of 37 °C for *Listeria monocytogenes* and *Escherichia coli*.

### Synthesis

The concept of the carbonate-based ionic liquid synthesis (CBILs)^38^ method was adapted to synthesize salts from piperonylic acid analogues (Scheme 1). Eight cations (Figure 1), employed as 1,3-dialkylimidazolium alkyl carbonates, were used as cation sources and reacted with the corresponding piperonylic acid analogues (Figure 1) in equimolar stoichiometric ratios in ethanol to afford the target salts. Upon reaction, the acid was converted to the corresponding conjugate anion, which induced hydrolysis of the alkyl carbonate, with the generation of CO_2_ and methanol as benign byproducts. The synthesized 32 distinct salts (Table S1) were characterized by NMR for their structural determination. NMR data for all 32 salts are available in (Supporting Information S2 and S7).

### Minimal Inhibitory Concentration (MIC) Assay

Minimal inhibitory concentrations (MICs) of the respective compounds were calculated by applying the serial 2-fold dilution microtiter plate method in TSB-Y medium.^43^ To create a synchronized cell status for each experiment, 1 mL aliquots of the respective overnight cultures were transferred into 9 mL of fresh TSB-Y medium and incubated for 3 h at respective temperatures. This ensured that cells were in a logarithmic growth phase. Subsequently, each well, which contained a serially diluted compound (dilution 1:2), was inoculated with 5 × 10^5^ CFU/mL of the respective bacterial cells. After bacterial inoculation, the 96-well microtiter plates were measured at a 610 nm wavelength in a Tecan F100 microplate reader (Tecan Austria GmbH, Groeding, Austria) to monitor any possible interference by compounds. The microtiter plates were then incubated for 24 h at respective temperatures, and afterward, bacterial growth was assessed by measuring absorbance at 610 nm. The MIC was defined as the lowest concentration of the respective compound where no bacterial growth could be measured after 24 h. Results are displayed as mean MICs, including upper and lower limits, of at least 3 experiments performed on different days. This allows for improved accuracy and reproducibility of results. Each experiment included positive and negative controls. The highest applied concentration for the experiment was 5000 mg/L. The assay was performed at least twice on different days (performed in two biological and two technical replicates).

### qPCR Enzyme Inhibition Assay

The p*rfA* gene assay was modified according to Sommer et al.^43^ using the nonspecific DNA dye EvaGreen (Jena Bioscience GmbH, Germany) to determine the toxicological effect of the salts at the enzyme level. Each qPCR reaction was performed in a final volume of 25 μL containing 2.5 μL of 10x reaction buffer (Biozym Scientific GmbH, Germany), 500 nM of each primer (LIP1: GAT ACA GAA ACA TCG GTT GGC and LIP2: GTG TAA TCT TGA TGC CAT CAG G (both Eurofins Genomics, Germany), 1.25 μM of probe EvaGreen, 200 μM of each dNTP (dATP, dTTP, dGTP, dCTP), 1 U of Taq DNA polymerase (Biozym Scientific GmbH, Hessisch Oldendorf, Germany), ∼150 copies of the internal amplification control *L. monocytogenes* EGDe (ATCC BAA-679), and 5 μL of the respective salt solution. Enzymatic inhibition of the tested compound was defined as the half-maximum effective concentration (EC_50_ value) in mg/mL, resulting in 50% efficiency of the qPCR reaction, determined by linear regression. Additionally, the minimal inhibitory concentration (MIC) was determined as the lowest concentration that prevents enzymatic activity. All the compounds were tested at concentrations of 50.000, 10.000, 2.000, and 400 mg/L in DMSO. All experiments were performed at least twice on different days, in duplicate (two biological and two technical replicates).

### AquaBoxil

SMILES representations for all cation–anion combinations of the 32 salts were initially generated using the free version of ChemSketch. Three-dimensional molecular structures were subsequently constructed and encoded in SMILES format using Open Babel (version 2.4.0).^53^ Geometry optimizations of the structures were performed using the PM7 semiempirical method as implemented in the MOPAC software package (Stewart, J. J. P. (2016), MOPAC2016, Stewart Computational Chemistry, Colorado Springs, CO, USA). The geometry of cations and anions was optimized separately, as indicated in the work of Rybinska et al.^54^ Molecular descriptors were calculated from the optimized structures using AlvaDesc.^55^ A total of 9 molecular descriptors required for the QSPR model equations, implemented in the AquaBoxIL^46^ tool, were selected. These descriptors were then used as input variables to estimate the biodegradability of the salts. The applicability domain of the results was evaluated using the leverage approach to ensure the reliability of the predictions.

### *In Vitro* Assays (Enzyme Inhibition Assays); Enzyme Source Preparation

Whole *Blattella germanica* (precooled at −20 °C) adults were used to prepare crude homogenates as enzyme sources. Four adults were mortared by adding 1 mL of phosphate buffer (50 mM, pH 7). The homogenized insect crude extract was added to an Eppendorf tube and then extracts were centrifuged at 10,000 g for 10 min to separate the supernatant from the biomass. The supernatant was collected, and protein estimation in the enzyme source was performed by the Bradford method with Bradford reagent using bovine serum albumin (BSA) as the standard. Concentrated enzyme source was diluted to 1 mg/mL in phosphate buffer (50 mM, pH 7) for performing enzyme assays. All experiments were performed at least twice on different days in duplicate (two biological and two technical replicates)

### Glutathione-*S*-Transferase (GST) Enzyme Inhibition Assay

The glutathione *S*-transferase (GST) enzyme inhibition assay was performed to measure the catalytic activity of GST using 1-chloro-2,4-dinitrobenzene (CDNB from Sigma-Aldrich) as the substrate, following existing protocols.^13^ The GST inhibition by piperonyl butoxide (PBO), free acids (piperonylic acid analogues), and salts was determined using a 96-well microplate format. Each reaction well contained a total volume of 200 μL. Reduced l-glutathione (GSH; 7 mM in 50 mM phosphate buffer, pH 7.0, Sigma-Aldrich, Product No. G4251) (120 μL) was first added to each well, followed by 50 μL of inhibitors prepared as serial dilutions in dimethyl sulfoxide (DMSO). Subsequently, 20 μL of the enzyme source was added to initiate preincubation. The final concentrations of inhibitors (PBO, free acids, and salts) in the reaction mixture ranged up to 100 mM. Blank controls without enzyme or without inhibitor were included in the assay. The reaction was initiated by adding 10 μL of freshly prepared 1-chloro-2,4-dinitrobenzene (CDNB; 20 mM in ethanol). GST-catalyzed conjugation of GSH with CDNB produced a chromophoric product, the formation of which was monitored by measuring the increase in absorbance at 340 nm at room temperature. Absorbance readings were recorded at 1-min intervals for 30 min using a SYNERGY H1 microplate reader (BioTek Instruments, USA). The assay was performed in duplicate on at least two different days. The percentage inhibition at each inhibitor concentration was calculated relative to the uninhibited control, and the half-maximal effective enzyme inhibitory concentration (EC_50_) values were obtained. The inhibitory potency of PBO, free acids, and salts on the GST enzyme was evaluated based on the derived EC_50_ values.

### Esterase (EST) Inhibition Assay

The esterase (EST) inhibition assay was adapted and optimized from methods previously described^13^ and performed using a 96-well microplate format. In each reaction, 308 μL of phosphate buffer (50 mM, pH 7.0) was mixed with 44 μL of salts as inhibitors (prepared as serial dilutions in DMSO) and 44 μL of enzyme source. The mixture was preincubated for 10 min at room temperature. Subsequently, 44 μL of α-naphthyl acetate substrate (1 mM in distilled water containing 1% acetone) was added, and the reaction was incubated for 10 min at 30 °C. The reaction was terminated by transferring 200 μL of the reaction mixture into a 96-well plate containing 20 μL of a Fast Garnet solution (10 mM) in distilled water with 35 mg/mL sodium dodecyl sulfate (SDS). The coupling reaction between α-naphthol and the diazonium salt produced a colored complex, and the absorbance was measured at 550 and 650 nm using a SYNERGY H1 microplate reader (BioTek Instruments, USA). Blank controls without enzyme or inhibitor were included in all assays. Each assay was performed in duplicate on at least two different days. The percentage inhibition at each inhibitor concentration was calculated relative to the uninhibited control, and half-maximal effective enzyme inhibitory concentration (EC_50_) values were obtained. The inhibitory potency of salts toward EST activity was evaluated across inhibitor concentrations up to 100 mM.

### *In Vivo* Assay (Spot On Test with *Blattella germanica*)

Synergistic efficacy was evaluated for piperonylic acid analogues (free acids) and the respective 32 salts, alone and in combination with pyrethrum (PYR). Free acids and salts were tested at final concentrations of 10 and 50 mg/mL, while pyrethrum was tested at a final concentration of 0.5 mg/mL. The respective solvents, DMSO and ethanol (96%), were also included as negative controls in each experiment. The knockdown effect was assessed after 30 min and 1 h of exposure, and mortality was recorded after 24 and 48 h. The knock-down^56^ effect refers to the rapid onset of paralysis upon exposure to insecticides and was identified by impaired coordination and reduced mobility, progressing to paralysis. Mortality refers to the death of the individual treated species. All test solutions were prepared by dilution in ethanol (96%). A volume of 1 μL of the test solution was applied topically to the pronotum of adult *Blattella germanica*. After treatment, insects were transferred to Petri dishes and maintained under laboratory conditions with access to water provided via a cotton pad. For each concentration, ten individuals of *Blattella germanica* were tested. Free acids and salts were applied either individually or in a 1:1 mixture with pyrethrum, yielding final concentrations of 10 or 50 mg/mL for free acids and salts and a final concentration of 0.5 mg/mL for pyrethrum. Pyrethrum (PYR) alone at the same concentration (0.5 mg/mL) was used as a reference control. Also, pyrethrum (PYR) with PBO (10 mg/mL) was used as the control. Each experiment was conducted at least three times on three different days.

### Statistical Analysis

Mortality rate was measured as described in the previous section and summarized as mean ± 95% CI. Assumptions for parametric analysis were assessed by visual inspection and by Shapiro–Wilk tests for normality and Brown–Forsythe tests for homogeneity of variances, and, with small sample sizes, these diagnostics were interpreted cautiously. Group differences were evaluated by one-way ANOVA, followed by Dunnett’s multiple comparisons test to compare each condition with the control. All tests were two-sided with α = 0.05, and multiplicity-adjusted p-values from Dunnett’s procedure are reported. Analyses and graphics were performed in GraphPad Prism (version 11.0.2; GraphPad Software).

## Conclusions

The present study demonstrates that selected piperonylic acid analogues possess synergistic activity in combination with pyrethroids against *B. germanica* and can inhibit key detoxification enzymes associated with insecticide resistance. These findings identify the compounds as promising lead candidates for the development of new synergistic agents. However, the observed activity should be considered preliminary, and additional investigations, including comprehensive toxicological assessment, environmental risk evaluation, pharmacokinetic characterization, and field validation, are required before their suitability as practical alternatives to piperonylbutoxide can be determined.

## Supplementary Material



## Notes

Additional information on experimental results in this study (Tables S1, S3, S4, S5, S6, S7): synthesis of salts (Table S1); detailed information about NMR data for salts (Table S2); minimum inhibitory concentration (MIC) of salts on bacteria (Table S3); minimum inhibitory concentration (MIC) and non inhibitory concentration (NIC) of salts on PCR enzyme inhibition (Table S4); predicted biodegradation for salts (Table S5); *in vitro* synergistic activity of piperonylic acid analogue salts on *Blattella germanica* (Table S6); synergistic activity of salts on *Blattella germanica* (Table S7); and original NMR spectra for salts (Table S8) (PDF)

## Abbreviations

CFU: colony forming unit
Std. Dev: standard deviation
Mol. Wt: molecular weight
Nr.: Number
[C_2_mim]^+^: 1-ethyl-3-methylimidazolium
[C_4_mim]^+^: 1-butyl-3-methylimidazolium
[TMC_4_A]^+^ or TMBA: Trimethylbutylammonium
[TMC_8_A]^+^: Trimethyloctylammonium
[TMC_16_A]^+^: Trimethylhexadecylammonium
[TC_4_MA]^+^: Tributymethylammonium
[TC_8_MA]^+^: trioctylmethylammonium.
