# Supplementary material for: Early-Stage Screening of Piperonylic Acid Analogues and Their Salts as Synergists for the Class of Pyrethroid Insecticides
Source: ACS Omega. 2026 Aug 26;11(35):53010–20. doi: 10.1021/acsomega.6c05301 (PMC13563995; doi:10.1021/acsomega.6c05301)

Early-stage screening of piperonylic acid analogues and their salts as synergists for the class of pyrethroid insecticides.

*Ramana Pydi,[a] Alexander Wolf,[b] Jennifer Bernard,[c] Christoph Zutz,[c] Birgit Bromberger,[a] Anita Sosnowska,[d,e] Szymon Zdybel,[d,e] Tomasz Puzyn,[d,e] Rolf Breinbauer[b] and Patrick-Julian Mikuni-Mester *[a]*

a. Centre for Food Science and Veterinary Public Health, Clinical Department for Farm Animals and Food System Science, University of Veterinary Medicine Vienna, Veterinaerplatz 1, 1210 Vienna, Austria. Email: Patrick-Julian.Mester@vetmeduni.ac.at
b. Institute of Organic Chemistry, Stremayrgasse 9, Graz University of Technology, 8010 Graz, Austria.
c. Kwizda Agro GmbH, Universitätsring 6, 1010 Vienna, Austria.
d. QSAR Lab, Grunwaldzka Ave, 103A, 80-244 Gdansk, Poland.
e. University of Gdansk, Faculty of Chemistry, Wita Stwosza 63, 80-308 Gdansk, Poland.

Supporting Information (SI)

Number of of Pages: 51;
Number of Tables: 6;
Number of NMR Spectra: 32.

Captions of Tables

Table S1. Synthesis of piperonylic acid analogues salts……………………………………S2
S2. NMR data for piperonylic acid analogues salts………………………………S3 to S15
Table S3. Minimum Inhibitory Concentration (MIC) of piperonylic acid analogues salts on Bacteria………………. …………………………………………………………………...S16
Table S4. Minimum Inhibitory Concentration (MIC) and Non Inhibitory Concentration (NIC) of piperonylic acid analogues salts on PCR Enzyme inhibition……………………….......S17
Table S5. Predicted biodegradation for piperonylic acid analogues salts………...……....S18
Table S6. *In vitro* synergistic activity of piperonylic acid analogues salts on *Blattella germanica*…………………………………………………………………………………S19
Table S7. Synergistic activity of piperonylic acid analogues salts on *Blattella germanica*…………………………………………………………………………………S20
S8. NMR spectra for piperonylic acid analogues salts……………………………S20 to S51

Table S1. Synthesis of piperonylic acid analogues salts

Nr.	Salts	Mol. Wt [g/mol]	Anion	Supplier	Cation	Supplier
**1**	[C_2mim][POAc]	276.3	Pieronylic Acid	Merck	[C_2mim]	Proionic GmbH
**2**	[C_4mim][POAc]	304.3	Pieronylic Acid	Merck	[C_4mim]	Proionic GmbH
**3**	[TMiPrA][POAc]	267.3	Pieronylic Acid	Merck	[TMiPrA]	Proionic GmbH
**4**	[TMC_4A][POAc]	281.3	Pieronylic Acid	Merck	[TMC_4A]	Proionic GmbH
**5**	[TMC_8A][POAc]	337.5	Pieronylic Acid	Merck	[TMC_8A]	Proionic GmbH
**6**	[TMC_{16}A][POAc]	449.7	Pieronylic Acid	Merck	[TMC_{16}A]	Proionic GmbH
**7**	[TC_4MA][POAc]	365.5	Pieronylic Acid	Merck	[TC_4MA]	Proionic GmbH
**8**	[TC_8MA][POAc]	533.8	Pieronylic Acid	Merck	[TC_8MA]	Proionic GmbH
**9**	[C_2mim][3,4-MPOAc]	290.3	3,4-(Methylenedioxy) phenyl acetic acid	Merck	[C_2mim]	Proionic GmbH
**10**	[C_4mim][3,4-MPOAc]	318.4	3,4-(Methylenedioxy) phenyl acetic acid	Merck	[C_4mim]	Proionic GmbH
**11**	[TMiPrA][3,4-MPOAc]	281.3	3,4-(Methylenedioxy) phenyl acetic acid	Merck	[TMiPrA]	Proionic GmbH
**12**	[TMC_4A][3,4-MPOAc]	295.4	3,4-(Methylenedioxy) phenyl acetic acid	Merck	[TMC_4A]	Proionic GmbH
**13**	[TMC_8A][3,4-MPOAc]	351.5	3,4-(Methylenedioxy) phenyl acetic acid	Merck	[TMC_8A]	Proionic GmbH
**14**	[TMC_{16}A][3,4-MPOAc]	463.7	3,4-(Methylenedioxy) phenyl acetic acid	Merck	[TMC_{16}A]	Proionic GmbH
**15**	[TC_4MA][3,4-MPOAc]	379.5	3,4-(Methylenedioxy) phenyl acetic acid	Merck	[TC_4MA]	Proionic GmbH
**16**	[TC_8MA][3,4-MPOAc]	547.9	3,4-(Methylenedioxy) phenyl acetic acid	Merck	[TC_8MA]	Proionic GmbH
**17**	[C_2mim][3,4-MCOAc]	302.3	3,4-(Methylenedioxy) cinnamic acid	Merck	[C_2mim]	Proionic GmbH
**18**	[C_4mim][3,4-MCOAc]	330.4	3,4-(Methylenedioxy) cinnamic acid	Merck	[C_4mim]	Proionic GmbH
**19**	[TMiPrA][3,4-MCOAc]	293.4	3,4-(Methylenedioxy) cinnamic acid	Merck	[TMiPrA]	Proionic GmbH
**20**	[TMC_4A][3,4-MCOAc]	307.4	3,4-(Methylenedioxy) cinnamic acid	Merck	[TMC_4A]	Proionic GmbH
**21**	[TMC_8A][3,4-MCOAc]	363.5	3,4-(Methylenedioxy) cinnamic acid	Merck	[TMC_8A]	Proionic GmbH
**22**	[TMC_{16}A][3,4-MCOAc]	475.7	3,4-(Methylenedioxy) cinnamic acid	Merck	[TMC_{16}A]	Proionic GmbH
**23**	[TC_4MA][3,4-MCOAc]	391.5	3,4-(Methylenedioxy) cinnamic acid	Merck	[TC_4MA]	Proionic GmbH
**24**	[TC_8MA][3,4-MCOAc]	559.9	3,4-(Methylenedioxy) cinnamic acid	Merck	[TC_8MA]	Proionic GmbH
**25**	[C_2mim][3,4-DCOAc]	318.4	3,4-Dimethoxycinnamic acid	Merck	[C_2mim]	Proionic GmbH
**26**	[C_4mim][3,4-DCOAc]	346.4	3,4-Dimethoxycinnamic acid	Merck	[C_4mim]	Proionic GmbH
**27**	[TMiPrA][3,4-DCOAc]	309.4	3,4-Dimethoxycinnamic acid	Merck	[TMiPrA]	Proionic GmbH
**28**	[TMC_4A][3,4-DCOAc]	323.4	3,4-Dimethoxycinnamic acid	Merck	[TMC_4A]	Proionic GmbH
**29**	[TMC_8A][3,4-DCOAc]	379.4	3,4-Dimethoxycinnamic acid	Merck	[TMC_8A]	Proionic GmbH

**30**	[$TMC_{16}A$][3,4-DCOAc]	491.7	3,4-Dimethoxycinnamic acid	Merck	[$TMC_{16}A$]	Proionic GmbH
**31**	[TC_4MA][3,4-DCOAc]	407.6	3,4-Dimethoxycinnamic acid	Merck	[TC_4MA]	Proionic GmbH
**32**	[TC_8MA][3,4-DCOAc]	575.9	3,4-Dimethoxycinnamic acid	Merck	[TC_8MA]	Proionic GmbH

Piperonylic acid (PA), 3,4-(methylenedioxy) phenyl acetic acid (3,4-MPA), 3,4-(methylenedioxy) cinnamic acid (3,4-MCA), 3,4-dimethoxycinnamic acid (3,4-DCA) were obtained directly from Merck and used as recieved (Technical grade purity).All cationic precursors were used at purity grades of >98% and were provided by proionic GmbH.

S2. NMR data for piperonylic acid analogues salts

All NMR spectra were recorded with a Jeol JNM-ECZL 400 MHz NMR Spectrometer (^{1}H: 399.78 MHz; ^{13}C: 100.53 MHz) with autosampler. Chemical shifts δ are referenced to residual protonated solvent signals as internal standard. Signal multiplicities J are abbreviated as s (singlet), d (doublet), dd (doublet of doublet), t (triplet) and m (multiplet). Deuterated solvents for NMR spectroscopy were purchased from Euriso-top. All original spectra can be found in S7 below.

1 [C_2mim][POAc]

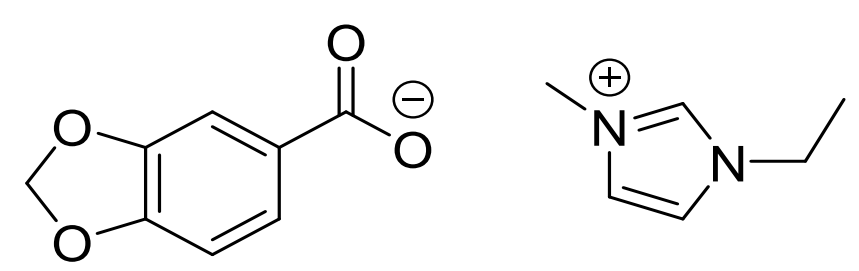

^{1}H-NMR (399.78 MHz, DMSO-d6) δ = 9.54 (s, 1H), 7.81 (t, J = 1.72 Hz, 1H), 7.73 (t, J = 1.72 Hz, 1H), 7.41 (dd, J = 7.41 Hz, 1.48 Hz, 1H), 7.31 (d, J = 1.48 Hz, 1H), 6.76 (d, J = 7.99 Hz, 1H), 5.97 (s, 2H), 4.20 (q, J = 7.32 Hz, 2H), 3.86 (s, 3H), 1.40 (t, J = 7.31 Hz, 3H) ppm.

^{13}C NMR (100.53 MHz, DMSO-d6) δ = 168.1, 148.1, 146.8, 137.3, 136.1, 124.1, 123.6, 122.5, 109.7, 107.2, 101.2, 44.6, 36.2, 15.7 ppm.

2 [C_4mim][POAc]

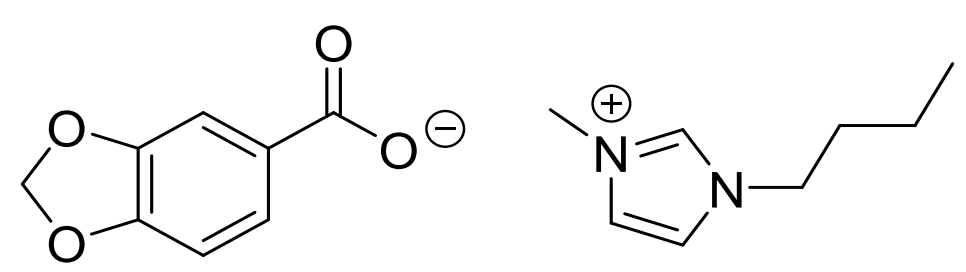

^{1}H-NMR (399.78 MHz, DMSO-d6) δ = 9.53 (s, 1H), 7.80 (s, 1H), 7.73 (s, 1H), 7.42 (d, J = 8.00 Hz, 1H), 7.32 (s, 1H), 6.76 (d, J = 8.00 Hz, 1H), 5.97 (s, 2H), 4.17 (t, J = 7.26 Hz, 2H), 3.87 (s, 3H), 1.75 (m, 2H), 1.24 (m, 2H) 0.88 (t, J = 7.40 Hz, 3H) ppm.

^{13}C NMR (100.53 MHz, DMSO-d6) δ = 167.5, 147.6, 146.3, 137.1, 135.1, 123.6, 123.1, 122.2, 109.1, 106.7, 100.7, 48.4, 35.6, 31.4, 13.2 ppm.

3 [TMiPrA][POAc]

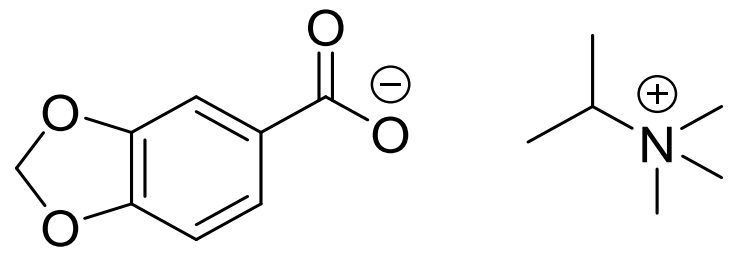

^{1}H-NMR (399.78 MHz, DMSO-d6) δ = 7.31 (dd, *J* = 7.96 Hz, 1.48 Hz, 1H), 7.29 (d *J* = 1.48 Hz, 1H), 6.72 (d, *J* = 7.96 Hz, 1H), 5.94 (s, 2H), 3.65 (m, 1H), 3.00 (s, 9H), 1.28 (dt, *J* = 6.60 Hz, 1.89 Hz, 6H) ppm.

^{13}C NMR (100.53 MHz, DMSO-d6) δ = 167.7, 147.5, 137.5, 123.3, 109.8, 107.0, 101.0, 66.5, 66.4, 50.3, 16.7 ppm.

4 [TMC$_4$A][POAc]

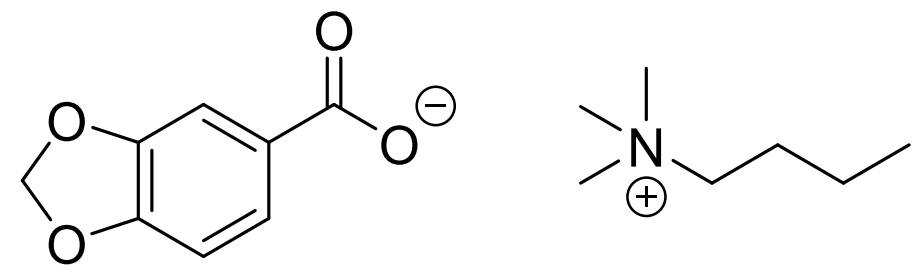

^{1}H-NMR (399.78 MHz, DMSO-d6) δ = 7.39 (d, J = 8.0 Hz, 1H), 7.30 (s, 1H), 6.73 (d, J = 8.0 Hz, 1H), 5.95 (s, 2H), 3.28 (t, J = 8.3 Hz, 2H), 3.06 (s, 9H), 1.63 (m, 2H), 1.27 (m, 2H), 0.91 (t, J = 7.3 Hz, 3H) ppm.

^{13}C NMR (100.53 MHz, DMSO-d6) δ = 167.5, 147.2, 146.1, 136.6, 123.0, 109.3, 106.5, 100.5, 65.1, 52.2, 52.1, 52.1, 24.1, 19.2, 13.5 ppm.

5 [TMC$_8$A][POAc]

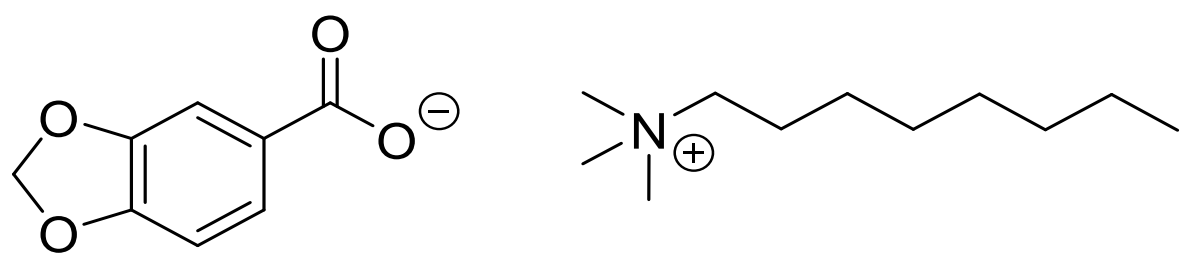

^{1}H-NMR (399.78 MHz, DMSO-d6) δ = 7.37 (d, *J* = 8.00 Hz, 1H), 7.28 (s, 1H), 6.71 (d, *J* = 8.00 Hz, 1H), 5.94 (s, 2H), 3.28 (m, 2H), 3.05 (s, 9H), 1.65 (m, 2H), 1.26 (s, 10H), 0.86 (m, 3H) ppm.

^{13}C NMR (100.53 MHz, DMSO-d6) δ = 167.7, 147.5, 146.6, 137.7, 123.3, 109.8, 107.0, 101.0, 65.8, 52.7, 52.6, 52.6, 31.7, 29.0, 26.3, 22.6, 14.5 ppm.

6 [$TMC_{16}A$][POAc]

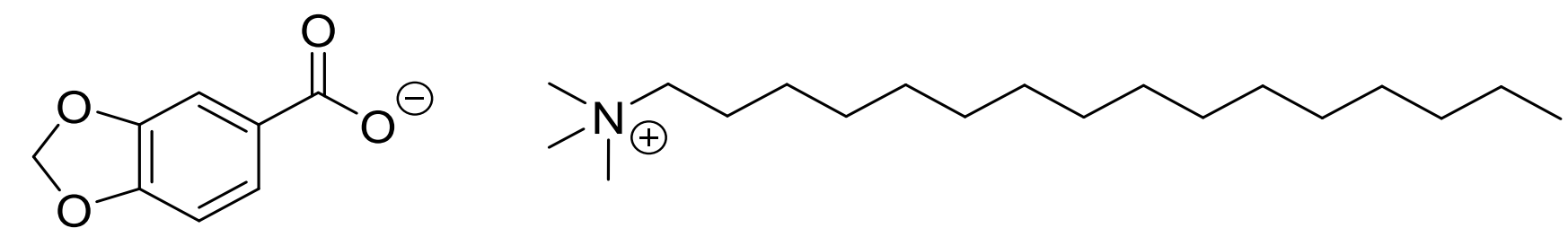

^{1}H-NMR (399.78 MHz, DMSO-d6) δ = 7.37 (d, *J* = 7.94 Hz, 1H), 7.29 (s, 1H), 6.70 (d, *J* = 7.94 Hz, 1H), 5.93 (s, 2H), 3.26 (m, 2H), 3.05 (s, 9H), 1.65 (m, 2H), 1.24 (s, 26H), 0.85 (m, 3H) ppm.

^{13}C NMR (100.53 MHz, DMSO-d6) δ = 167.7, 147.4, 146.6, 123.3, 109.8, 106.9, 100.9, 65.8, 52.7, 52.6, 52.6, 31.8, 29.6, 29.5, 29.5, 29.4, 29.2, 29.0, 26.3, 22.6, 22.6, 14.5 ppm.

7 [TC_4MA][POAc]

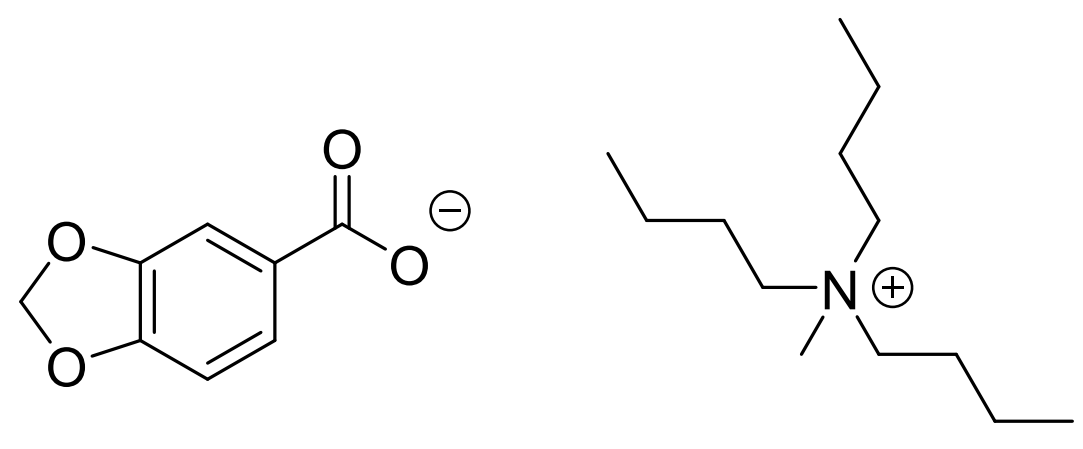

^{1}H-NMR (399.78 MHz, DMSO-d6) δ = 7.41 (d, *J* = 8.00 Hz, 1H), 7.30 (s, 1H), 6.76 (d, *J* = 8.00 Hz, 1H), 5.97 (s, 2H), 3.20 (m, 6H), 2.95 (s, 3H), 1.58 (m, 6H), 1.30 (m, 6H), 0.93 (t, *J* = 7.40 Hz, 9H) ppm.

^{13}C NMR (100.53 MHz, DMSO-d6) = 167.7, 148.0, 146.8, 123.6, 109.7, 107.2, 101.2, 61.0, 48.1, 23.9, 19.8, 14.0 ppm.

8 $[TC_8MA][POAc]$

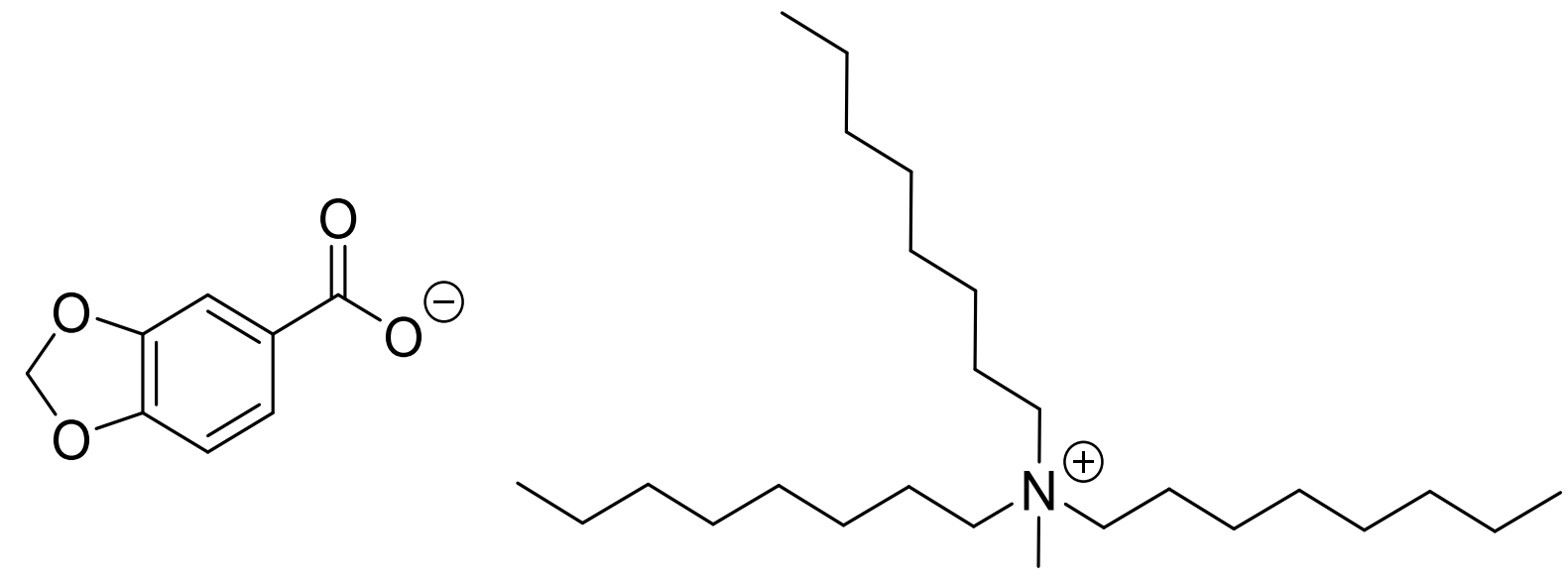

^{1}H-NMR (399.78 MHz, DMSO-d6) δ = 7.38 (d, *J* = 7.92 Hz, 1H), 7.29 (s, 1H), 6.71 (d, *J* = 7.92 Hz, 1H), 5.94 (s, 2H), 3.19 (m, 6H), 2.95 (s, 3H), 1.60 (s, 6H), 1.27 (s, 30H), 0.87 (m, 9H) ppm.

^{13}C NMR (100.53 MHz, DMSO-d6) δ = 167.6, 147.6, 146.6, 137.5, 123.4, 109.8, 106.9, 101.0, 61.0, 31.7, 29.0, 28.9, 26.3, 22.6, 21.9, 14.5 ppm.

9 $[C_2mim][3,4\text{-}MPOAc]$

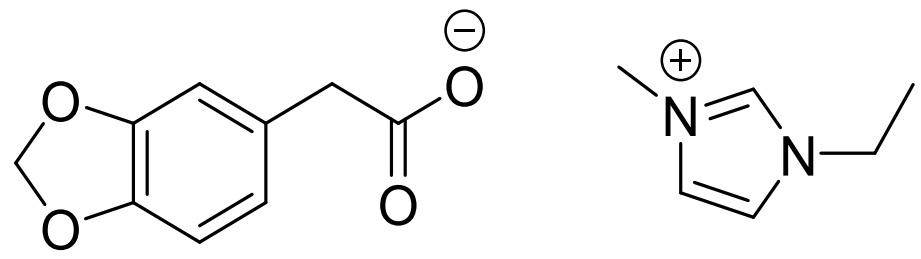

^{1}H-NMR (399.78 MHz, DMSO-d6) δ = 9.59 (s, 1H), 7.79 (t, *J* = 1.73 Hz, 1H), 7.71 (t, *J* = 1.73 Hz, 1H), 6.78 (d, *J* = 1.62 Hz, 1H), 6.70 (d, *J* = 7.87 Hz, 1H), 6.59 (m, 1H), 5.90 (s, 2H), 4.17 (q, *J* = 7.31 Hz, 2H), 3.83 (s, 3H), 3.09 (s, 2H), 1.39 (t, *J* = 7.31 Hz, 3H) ppm.

^{13}C NMR (100.53 MHz, DMSO-d6) δ = 173.3, 147.0, 145.0, 137.5, 134.8, 124.0, 122.4, 122.2, 110.3, 107.8, 100.8, 46.5, 44.5, 36.1, 15.7 ppm.

10 $[C_4mim][3,4\text{-}MPOAc]$

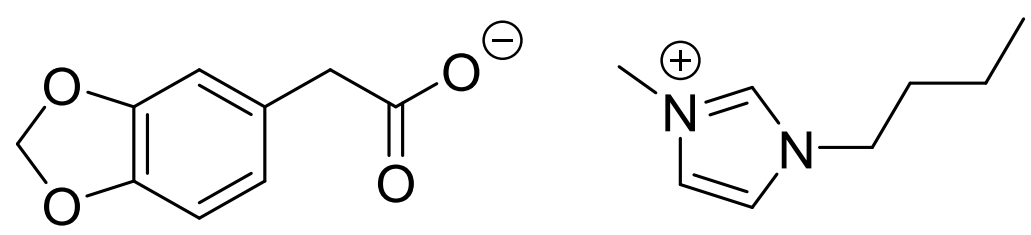

^{1}H-NMR (399.78 MHz, DMSO-d6) δ = 9.38 (s, 1H), 7.76 (s, 1H), 7.69 (s, 1H), 6.79 (s, 1H), 6.71 (d, *J* = 7.90 Hz, 1H), 6.61 (d, *J* = 7.90 Hz, 1H), 5.90 (s, 2H), 4.14 (t, *J* = 7.23 Hz, 2H), 3.83 (s, 3H), 3.13 (s, 2H), 1.74 (m, 2H), 1.24 (m, 2H), 0.88 (t, *J* = 7.41 Hz, 3H) ppm.

^{13}C NMR (100.53 MHz, DMSO-d6) δ = 173.7, 147.1, 145.1, 137.5, 134.1, 124.1, 122.7, 122.2, 110.3, 107.9, 100.8, 48.9, 45.9, 36.2, 31.9, 19.3, 13.8 ppm.

11 [TMiPrA][3,4-MPOAc]

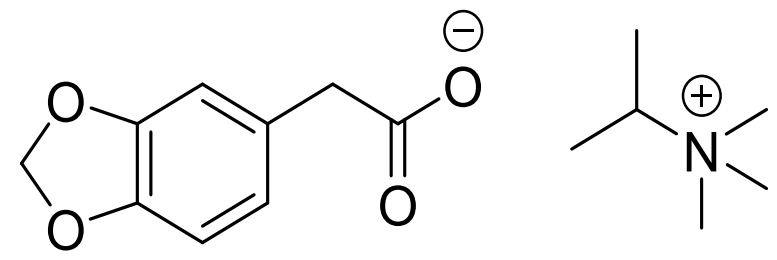

^{1}H-NMR (399.78 MHz, DMSO-d6) δ = 6.78 (d, *J* = 1.60 Hz, 1H), 6.70 (d, *J* = 7.87 Hz, 1H), 6.58 (dd, *J* = 7.88 Hz, 1.60 Hz, 1H), 5.90 (s, 2H), 3.64 (m, 1H), 3.03 (s, 2H), 2.98 (s, 9H), 1.27 (dt, *J* = 6.61 Hz, 1.89 Hz, 6H) ppm.

^{13}C NMR (100.53 MHz, DMSO-d6) δ = 172.8, 147.0, 144.8, 135.3, 122.1, 110.3, 107.7, 100.7, 66.5, 50.3, 47.1, 16.7 ppm.

12 [TMC$_4$A][3,4-MPOAc]

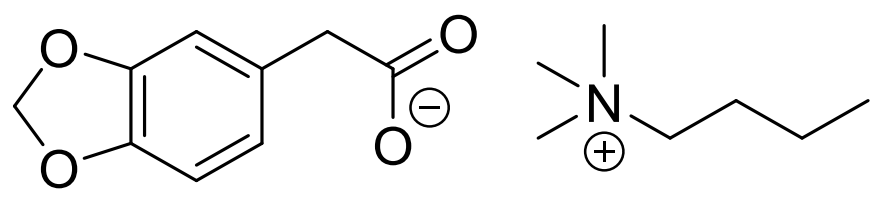

^{1}H-NMR (399.78 MHz, DMSO-d6) δ = 6.79 (s, 1H), 6.70 (d, *J* = 7.6 Hz, 1H), 6.59 (d, *J* = 7.6 Hz, 1H), 5.90 (s, 2H), 3.27 (m, 2H), 3.04 (s, 9H), 1.62 (m, 2H), 1.28 (m, 2H), 0.92 (t, J = 7.2 Hz, 3H) ppm.

^{13}C NMR (100.53 MHz, DMSO-d6) δ = 172.6, 146.5, 144.4, 134.7, 121.6, 109.8, 107.3 100.2, 65.1, 52.1, 52.1, 52.1, 46.5, 24.1, 19.2, 13.5 ppm.

13 [TMC$_8$A][3,4-MPOAc]

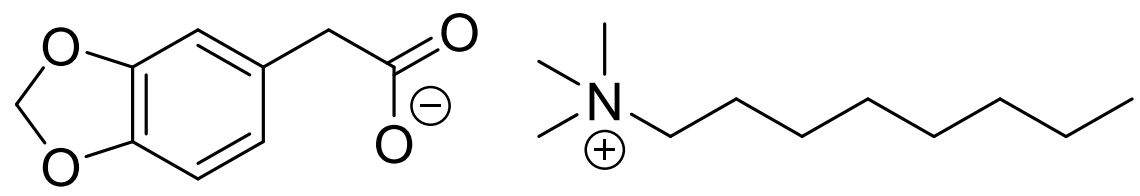

^{1}H-NMR (399.78 MHz, DMSO-d6) δ = 6.77 (s, 1H), 6.69 (d, J = 7.8 Hz, 1H), 6.57 (d, J = 7.8 Hz, 1H), 5.90 (s, 2H), 3.25 (m, 2H), 3.03 (s, 9H), 3.01 (s, 2H), 1.65 (m, 2H), 1.28 (m, 10H), 0.87 (m, 3H) ppm.

^{13}C NMR (100.53 MHz, DMSO-d6) δ = 172.2, 146.5, 144.3, 134.9, 121.5, 109.8, 107.2, 100.1, 65.3, 52.1, 52.1, 52.0, 46.6, 31.2, 28.5, 25.8, 22.0, 14.0 ppm.

14 [TMC$_{16}$A][3,4-MPOAc]

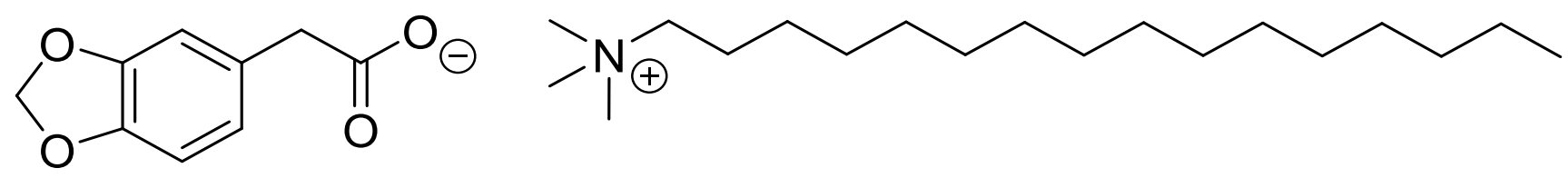

^{1}H-NMR (399.78 MHz, DMSO-d6) δ = 6.78 (s, 1H), 6.71 (d, *J* = 7.70 Hz, 1H), 6.60 (d, *J* = 7.70 Hz, 1H) 5.91 (s, 2H), 3.25 (m, 2H), 3.12 (s, 2H), 3.03 (s, 9H), 1.64 (m, 2H), 1.24 (s, 26H), 0.85 (m, 3H) ppm.

^{13}C NMR (100.53 MHz, DMSO-d6) δ = 173.2, 147.1, 145.1, 122.2, 110.3, 107.9, 100.8, 65.7, 52.6, 45.8, 31.8, 29.6, 29.5, 29.4, 29.3, 29.2, 29.0, 26.3, 22.6, 22.6, 14.5 ppm.

15 [TC$_{4}$MA][3,4-MPOAc]

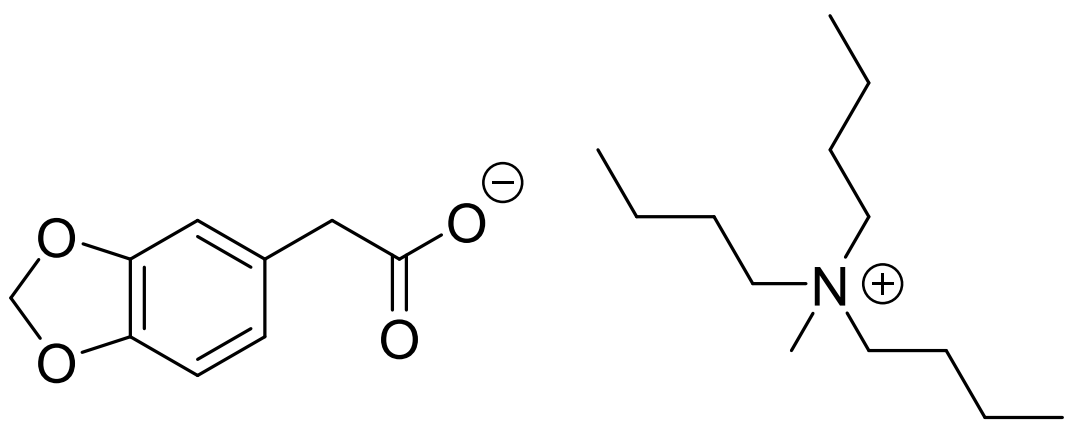

^{1}H-NMR (399.78 MHz, DMSO-d6) δ = 6.78 (s, 1H), 6.71 (d, *J* = 8.03 Hz, 1H), 6.59 (d, *J* = 8.03 Hz, 1H), 5.91 (s, 2H), 3.20 (m, 6H), 3.09 (s, 2H), 2.94 (s, 3H), 1.59 (m, 6H), 1.30 (s, 6H), 0.93 (t, *J* = 7.38 Hz, 9H) ppm.

^{13}C NMR (100.53 MHz, DMSO-d6) = 172.9, 147.0, 145.0, 134.6, 122.2, 110.3, 107.8, 100.7, 60.9, 48.1, 48.1, 48.0, 46.2, 23.9, 19.8, 14.0 ppm.

16 [TC$_{8}$MA][3,4-MPOAc]

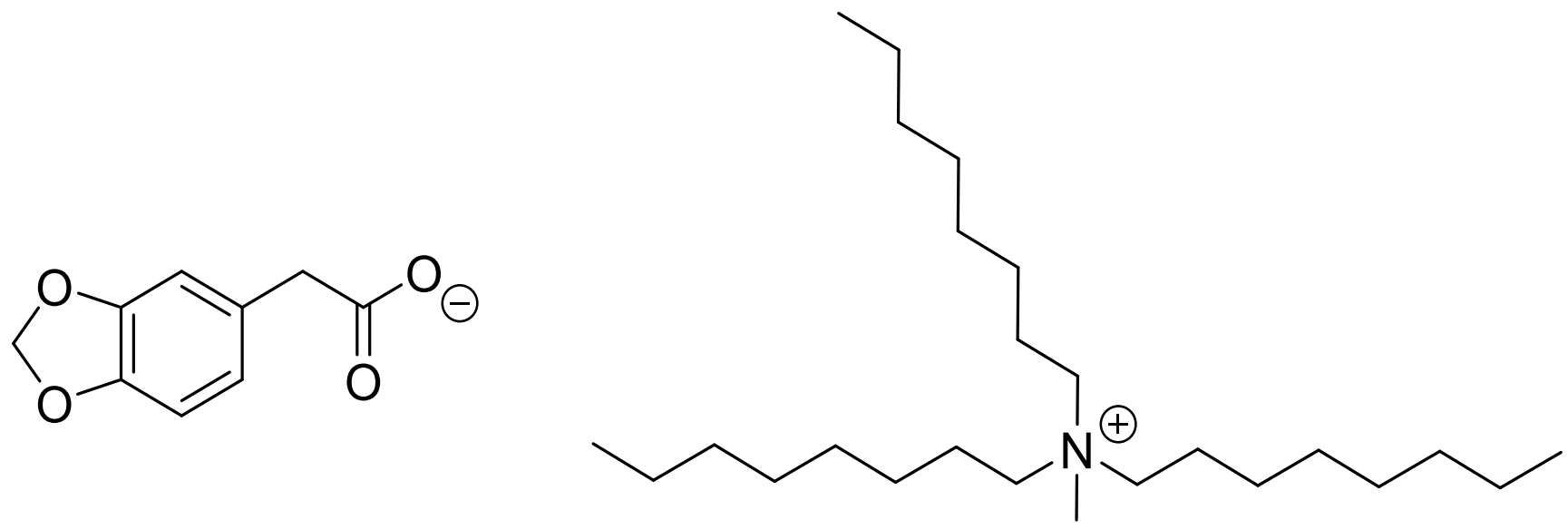

^{1}H-NMR (399.78 MHz, DMSO-d6) δ = 6.77 (s, 1H), 6.68 (d, *J* = 7.88 Hz, 1H), 6.58 (d, *J* = 7.88 Hz, 1H), 5.89 (s, 2H), 3.18 (m, 6H), 3.03 (s, 2H), 2.93 (s, 3H), 1.59 (s, 6H), 1.26 (s, 30H), 0.86 (m, 9H) ppm.

^{13}C NMR (100.53 MHz, DMSO-d6) δ = 172.8, 147.0, 144.8, 135.2, 122.1, 110.3, 107.7, 100.7, 61.0, 46.9, 31.7, 29.0, 28.9, 26.3, 22.6, 21.9, 14.5 ppm.

17 [C$_2$mim][3,4-MCOAc]

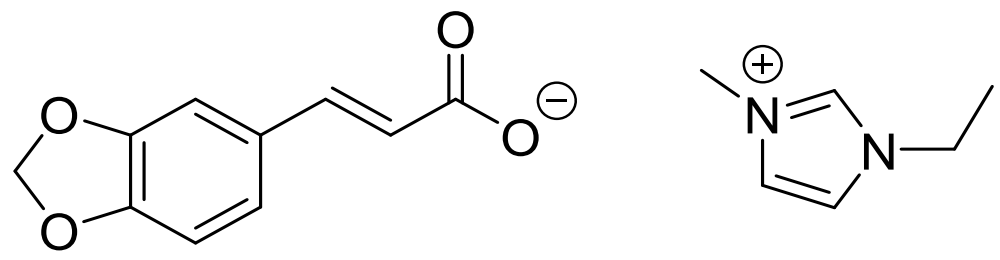

^{1}H-NMR (399.78 MHz, DMSO-d6) δ = 9.64 (s, 1H), 7.81 (t, *J* = 1.70 Hz, 1H), 7.73 (t, *J* = 1.70 Hz, 1H), 7.06 (d, *J* = 1.60 Hz, 1H), 6.94 (d, *J* = 15.8 Hz, 1H), 6.88 (dd, *J* = 8.00 Hz, 1.60 Hz, 1H) 6.84 (d, *J* = 7.97 Hz, 1H), 6.20 (d, *J* = 15.8 Hz, 1H), 6.00 (s, 2H), 4.21 (q, *J* = 7.32 Hz, 2H), 3.86 (s, 3H), 1.40 (t, *J* = 7.30 Hz, 3H) ppm.

^{13}C NMR (100.53 MHz, DMSO-d6) δ = 169.9, 148.2, 147.4, 137.5, 134.6, 132.1, 130.5, 124.1, 122.5, 122.1, 108.8, 106.3, 101.5, 44.6, 36.1, 15.7 ppm.

18 [C$_4$mim][3,4-MCOAc]

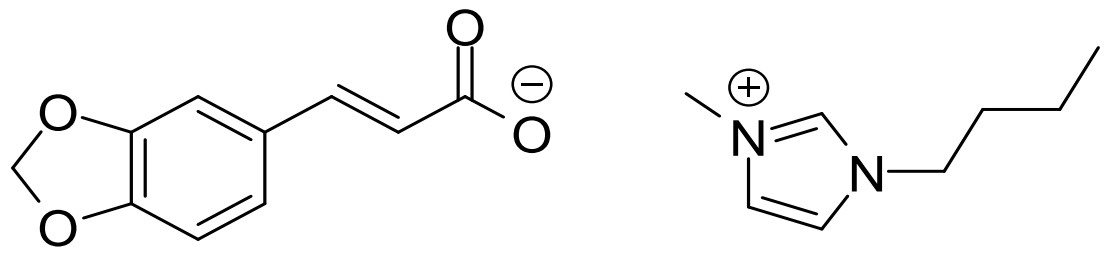

^{1}H-NMR (399.78 MHz, DMSO-d6) δ = 9.9 (s, 1H), 7.80 (s, 1H), 7.73 (s, 1H), 7.07 (s, 1H), 6.97 (d, *J* = 15.8 Hz, 1H), 6.89 (d, *J* = 8.00 Hz, 1H) 6.85 (d, *J* = 8.00 Hz, 1H), 6.23 (d, *J* = 15.8 Hz, 1H), 6.00 (s, 2H), 4.18 (t, *J* = 7.12 Hz, 2H), 3.87 (s, 3H), 1.76 (m, 2H), 1.24 (q, J = 7.45 Hz, 2H) 0.88 (t, *J* = 7.34 Hz, 3H) ppm.

^{13}C NMR (100.53 MHz, DMSO-d6) δ = 169.3, 147.7, 147.0, 137.2, 134.4, 131.5, 129.4, 123.6, 122.2, 121.7, 108.2, 105.8, 101.0, 48.4, 35.6, 31.4, 13.2 ppm.

19 [TMiPrA][3,4-MCOAc]

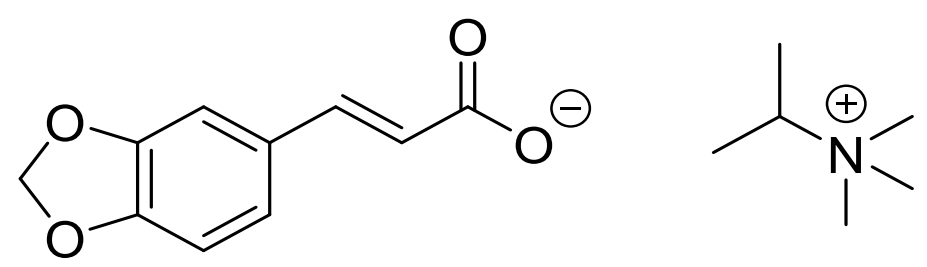

^{1}H-NMR (399.78 MHz, DMSO-d6) δ = 7.05 (d, *J* = 1.60 Hz, 1H), 6.91(d, *J* = 15.9 Hz, 1H), 6.87 (d, *J* = 1.60 Hz, 1H) 6.85 (d, *J* = 7.95 Hz, 1H), 6.18 (d, *J* = 15.8 Hz, 1H), 6.00 (s, 2H), 3.65 (m, 1H), 3.00 (s, 9H), 1.28 (dt, *J* = 6.60 Hz, 1.90 Hz, 6H) ppm.

^{13}C NMR (100.53 MHz, DMSO-d6) δ = 169.6, 148.2, 147.4, 134.6, 132.2, 130.7, 122.1, 108.8, 106.3, 101.5, 66.5, 50.4, 50.3, 50.2, 16.7 ppm.

20 [TMC$_4$A][3,4-MCOAc]

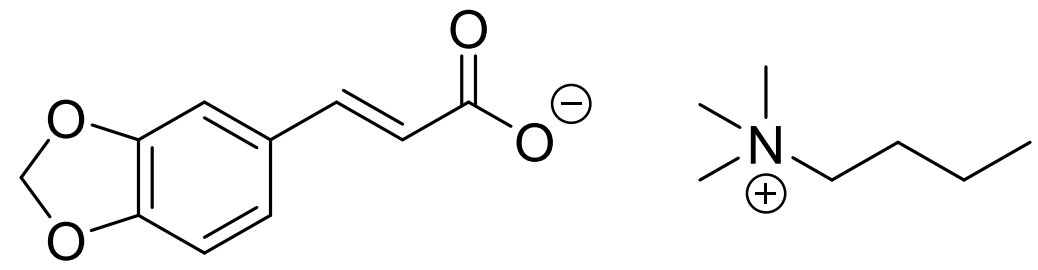

^{1}H-NMR (399.78 MHz, DMSO-d6) δ = 7.05 (s, 1H), 6.89 (m, 3H), 6.18 (d, *J* = 16.2 Hz, 1H), 6.00 (s, 2H), 3.29 (m, 2H), 3.06 (s, 9H), 1.64 (m, 2H), 1.28 (m, 2H), 0.914 (t, *J* = 7.1 Hz, 3H) ppm.

^{13}C NMR (100.53 MHz, DMSO-d6) δ = 169.2, 147.7, 146.9, 134.1, 131.6, 130.0, 121.6, 108.3, 105.8, 101.0, 65.1, 52.2, 52.1, 52.1, 24.1, 19.2, 13.5 ppm.

21 [TMC$_8$A][3,4-DCOAc]

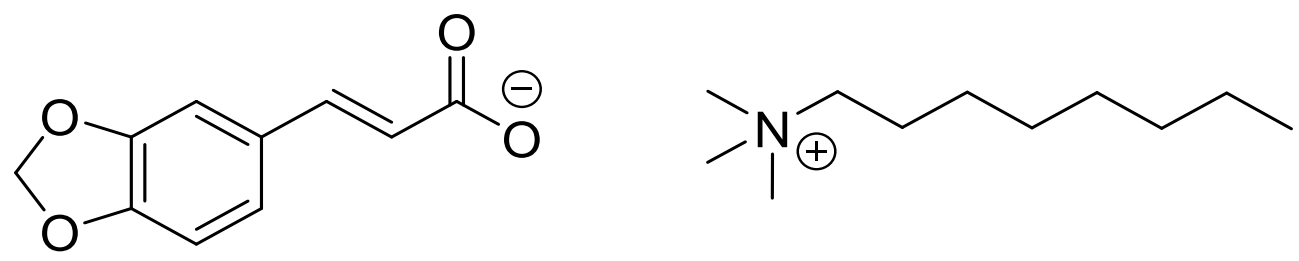

^{1}H-NMR (399.78 MHz, DMSO-d6) δ = 7.04 (s, 1H), 6.87 (m, 3H), 6.17 (d, *J* = 15.5 Hz, 1H), 5.99 (s, 2H), 3.28 (m, 2H), 3.05 (s, 9H), 1.65 (m, 2H), 1.26 (m, 10H), 0.86 (t, *J* = 6.33 Hz, 3H) ppm.

^{13}C NMR (100.53 MHz, DMSO-d6) δ = 168.9, 147.6, 146.8, 133.6, 131.7, 130.5, 121.4, 108.2, 105.8, 100.9, 65.2, 52.1, 52.1, 52.0, 31.1, 28.5, 25.8, 22.0, 13.9 ppm.

22 [TMC$_{16}$A][3,4-MCOAc]

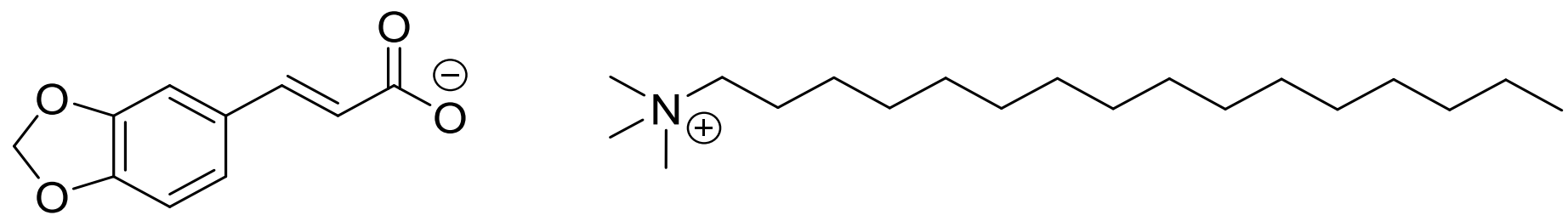

^{1}H-NMR (399.78 MHz, DMSO-d6) δ = 7.06 (s, 1H), 6.94 (d, *J* = 15.8 Hz, 1H), 6.89 (d, *J* = 8.00 Hz, 1H), 6.84 (d, *J* = 6.84 Hz, 1H), 6.21 (d, *J* = 15.8 Hz, 1H), 6.00 (s, 2H), 3.27 (t, *J* = 8.08 Hz, 2H), 3.05 (s, 9H), 1.65 (m, 2H), 1.23 (s, 26H), 0.86 (m, 3H) ppm.

^{13}C NMR (100.53 MHz, DMSO-d6) δ = 169.0, 147.7, 147.0, 134.4, 131.5, 129.5, 121.6, 108.2, 105.8, 100.9, 65.3, 52.1, 52.1, 52.0, 31.3, 29.0, 29.0, 28.9, 28.8, 28.7, 28.5, 25.8, 22.1, 22.0, 13.9 ppm.

23 [TC_4MA][3,4-MCOAc]

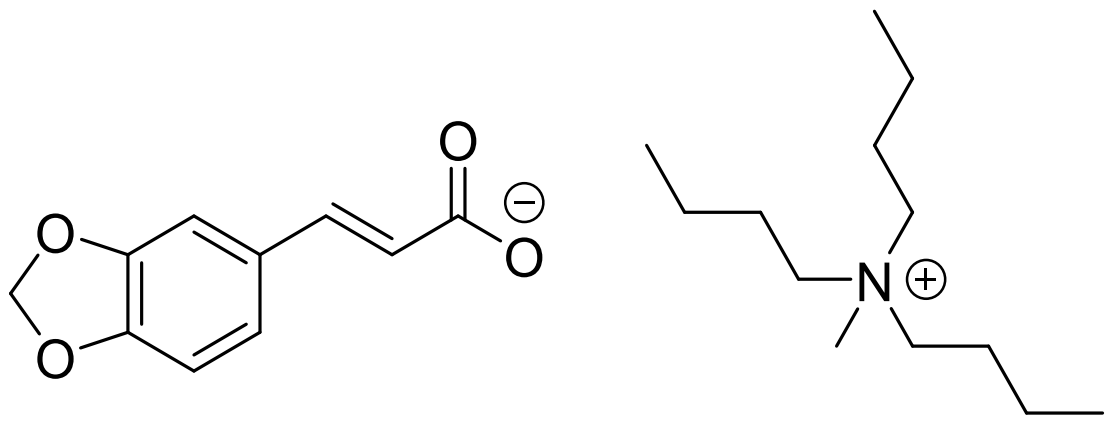

^{1}H-NMR (399.78 MHz, DMSO-d6) δ = 7.06 (s, 1H), 6.95 (d, *J* = 15.8 Hz, 1H), 6.89 (d, *J* = 7.80 Hz, 1H), 6.85 (d, *J* = 7.80 Hz, 1H), 6.22 (d, *J* = 15.8 Hz, 1H), 6.00 (s, 2H), 3.21 (t, *J* = 8.12 Hz, 6H), 2.96 (s, 3H), 1.58 (m, 6H), 1.30 (q, *J* = 7.30 Hz, 6H), 0.92 (t, *J* = 7.30 Hz, 9H) ppm.

^{13}C NMR (100.53 MHz, DMSO-d6) δ = 168.9, 147.7, 146.9, 134.3, 131.5, 129.5, 121.6, 108.2, 105.8, 100.9, 60.4, 23.4, 13.5 ppm.

24 [TC_8MA][3,4-MCOAc]

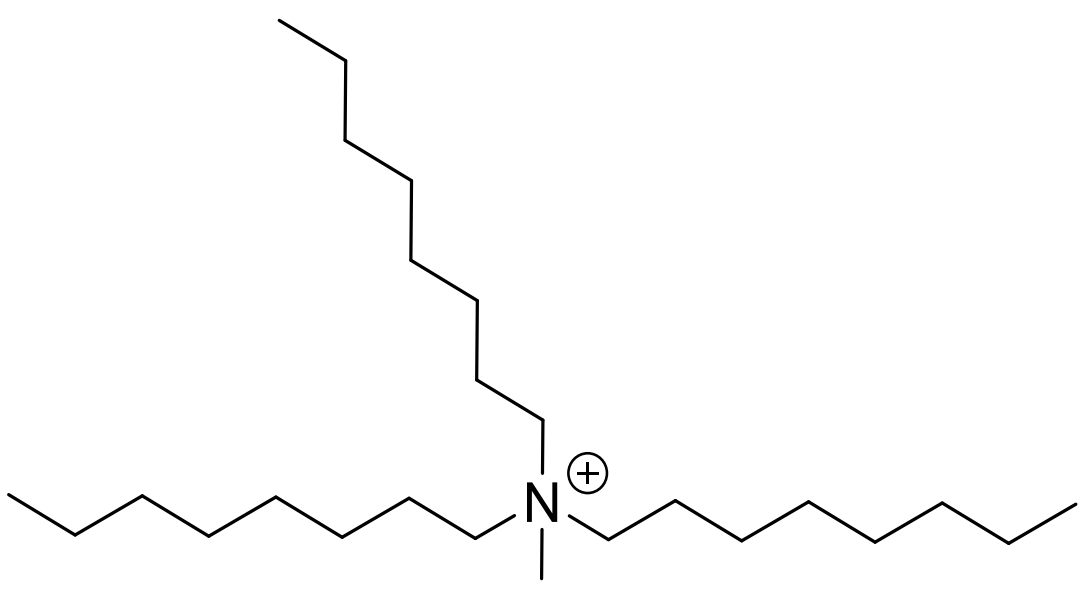

^{1}H-NMR (399.78 MHz, DMSO-d6) δ = 7.04 (s, 1H), 6.87 (m, 3H), 6.18 (d, *J* = 15.8 Hz, 1H), 6.00 (s, 2H), 3.19 (m, 6H), 2.95 (s, 3H), 1.60 (s, 6H), 1.26 (m, 30H), 0.92 (m, 9H) ppm.

^{13}C NMR (100.53 MHz, DMSO-d6) δ = 169.4, 148.2, 147.4, 134.4, 132.2, 130.6, 122.0, 108.7, 106.3, 101.5, 61.0, 48.0, 31.7, 29.0, 29.0, 26.3, 22.6, 21.9, 14.5 ppm.

25 [C_2mim][3,4-DCOAc]

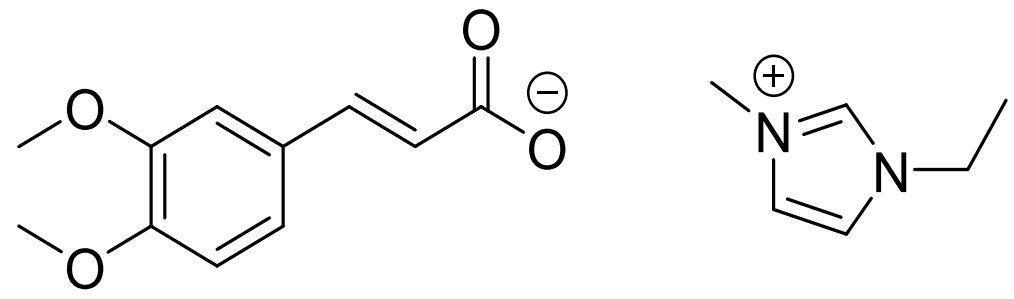

^{1}H-NMR (399.78 MHz, DMSO-d6) δ = 9.59 (s, 1H), 7.81 (t, *J* = 1.72 Hz, 1H), 7.72 (t, *J* = 1.72 Hz, 1H), 7.07 (d, *J* = 1.90 Hz, 1H), 6.98 (d, *J* = 15.9 Hz, 1H), 6.94 (d, *J* = 1.90 Hz, 1H), 6.89 (d, *J* = 8.27 Hz, 1H), 6.27 (d, *J* = 15.9 Hz, 1H), 4.20 (q, *J* = 7.32 Hz, 2H), 3.86 (s, 3H), 3.77 (s, 3H), 3.74 (s, 3H), 1.41 (t, *J* = 7.31 Hz, 3H) ppm.

^{13}C NMR (100.53 MHz, DMSO-d6) δ = 170.1, 149.3, 149.2, 137.4, 135.2, 130.5, 129.7, 124.1, 122.5, 120.6, 112.2, 110.0, 56.0, 55.9, 44.6, 36.2, 15.7 ppm.

26 [C_4mim][3,4-DCOAc]

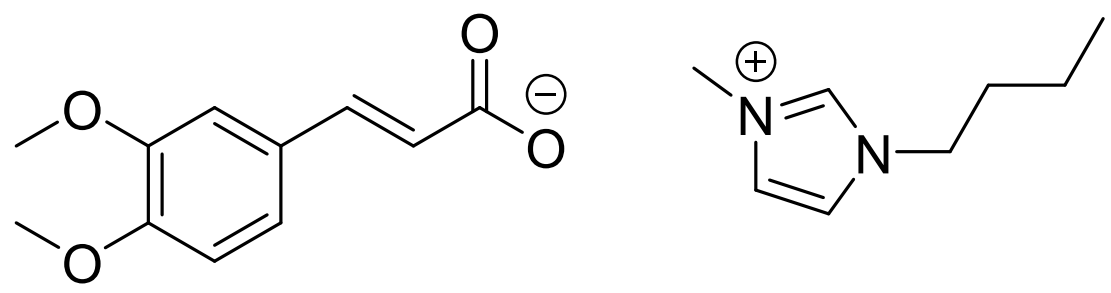

^{1}H-NMR (399.78 MHz, DMSO-d6) δ = 9.54 (s, 1H), 7.79 (s, 1H), 7.73 (s, 1H), 7.10 (s, 1H), 7.03 (d, *J* = 15.8 Hz, 1H), 6.97 (d, *J* = 8.25 Hz, 1H) 6.90 (d, *J* = 8.25 Hz, 1H), 6.31 (d, *J* = 15.8 Hz, 1H), 4.17 (t, *J* = 7.27 Hz, 2H), 3.87 (s, 3H), 3.77 (s, 3H), 3.75 (s, 3H), 1.76 (m, 2H), 1.24 (m, 2H), 0.89 (t, *J* = 7.41 Hz, 3H) ppm.

^{13}C NMR (100.53 MHz, DMSO-d6) δ = 169.4, 148.8, 148.8, 137.1, 135.4, 129.7, 128.1, 123.6, 122.2, 120.3, 111.7, 109.6, 55.4, 55.4, 48.4, 35.6, 31.4, 13.2 ppm.

27 [TMiPrA][3,4-DCOAc]

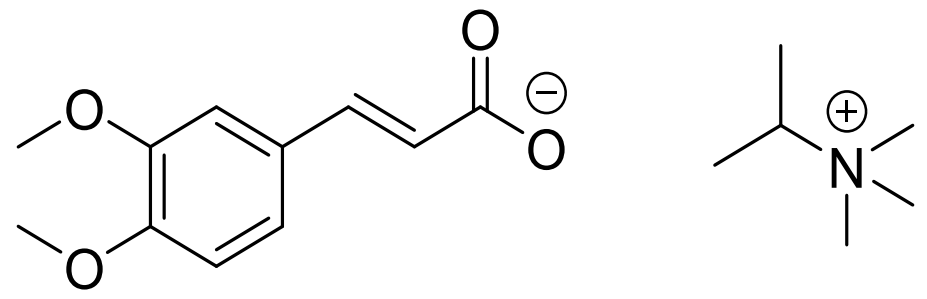

^{1}H-NMR (399.78 MHz, DMSO-d6) δ = 7.05 (d, *J* = 1.90 Hz, 1H), 6.91 (m, 3H), 6.21 (d, *J* = 15.8 Hz, 1H), 3.77 (s, 3H), 3.74 (s, 3H), 3.65 (m, 1H), 2.99 (s, 9H), 1.28 (dt, *J* = 6.60 Hz, 1.89 Hz, 6H) ppm.

^{13}C NMR (100.53 MHz, DMSO-d6) δ = 169.6, 149.3, 149.1, 134.5, 130.7, 130.6, 120.5, 112.2, 122.5, 120.6, 112.2, 110.0, 56.0, 55.9, 50.4, 50.3, 50.2, 16.7 ppm.

28 [TMC$_4$A][3,4-DCOAc]

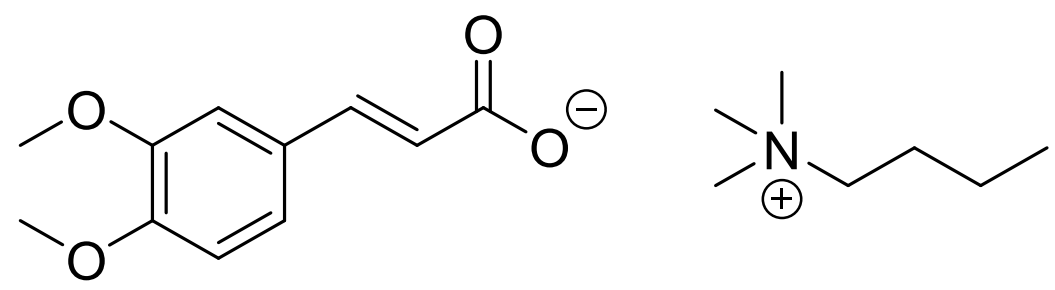

^{1}H-NMR (399.78 MHz, DMSO-d6) δ = 7.05 (s, 1H), 6.93 (m, 3H), 6.23 (d, *J* = 15.7 Hz, 1H), 3.77 (s, 3H), 3.74 (s, 3H), 3.29 (m, 2H), 3.06 (s, 9H), 1.64 (m, 2H), 1.28 (m, 2H), 0.91 (t, *J* = 7.2 Hz, 3H) ppm.

^{13}C NMR (100.53 MHz, DMSO-d6) δ = 169.5, 148.9, 148.7, 134.4, 130.1, 129.6, 120.1, 111.8, 109.6, 65.1, 55.5, 55.4, 52.2, 52.1, 52.1, 24.1, 19.2, 13.5 ppm.

29 [TMC$_8$A][3,4-DCOAc]

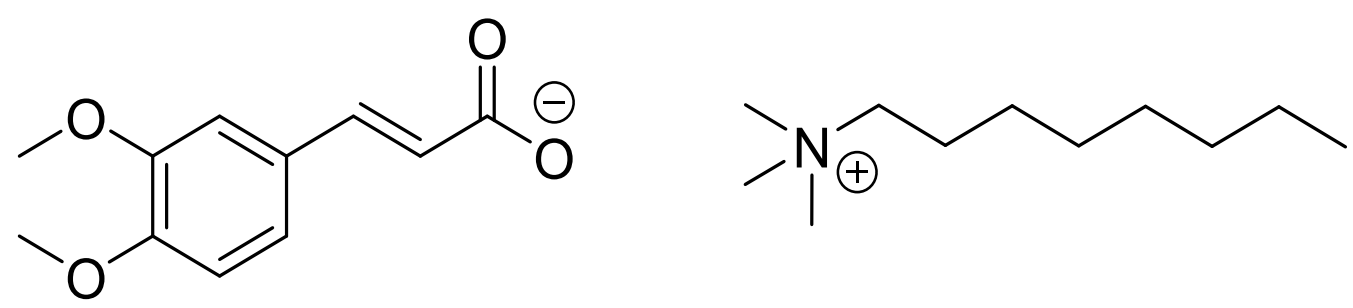

^{1}H-NMR (399.78 MHz, DMSO-d6) δ = 7.04 (s, 1H), 6.90 (m, 3H), 6.21 (d, *J* = 15.8 Hz, 1H), 3.78 (s, 3H), 3.74 (s, 3H), 3.28 (t, *J* = 8.34 Hz, 2H), 3.05 (s, 9H), 1.65 (m, 2H), 1.27 (m, 10H), 0.86 (m, 3H) ppm.

^{13}C NMR (100.53 MHz, DMSO-d6) δ = 169.6, 149.4, 149.1, 134.3, 130.8, 120.4, 112.3, 110.0, 65.8, 55.9, 52.7, 52.6, 52.6, 31.7, 29.0, 26.3, 22.6, 14.5 ppm.

30 [TMC$_{16}$A][3,4-DCOAc]

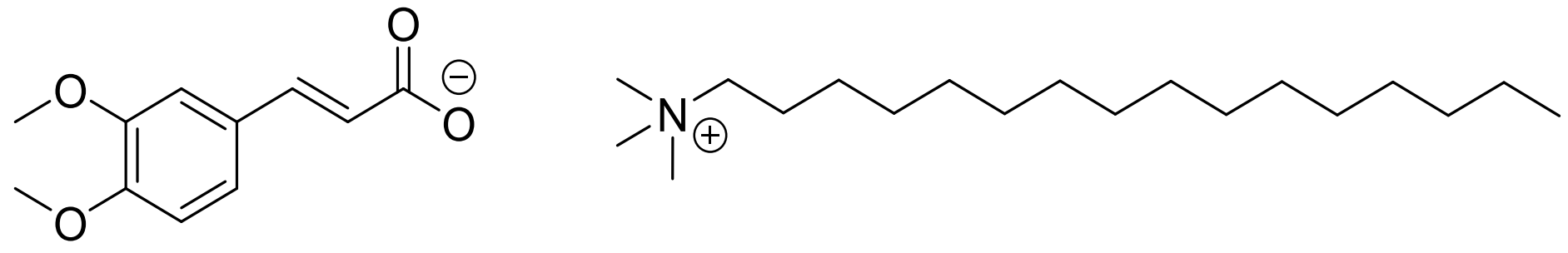

^{1}H-NMR (399.78 MHz, DMSO-d6) δ = 7.09 (m, 2H), 6.98 (d, *J* = 8.26 Hz, 1H), 6.90 (d, *J* = 8.26 Hz, 1H), 6.35 (d, *J* = 15.8 Hz, 1H), 3.77 (s, 3H), 3.75 (s, 3H), 3.27 (t, *J* = 8.31 Hz, 2H), 3.05 (s, 9H), 1.64 (m, 2H), 1.23 (s, 26H), 0.85 (m, 3H) ppm.

^{13}C NMR (100.53 MHz, DMSO-d6) δ = 169.2, 149.0, 148.8, 136.0, 129.5, 127.2, 120.4, 111.7, 109.7, 65.3, 55.5, 55.4, 52.1, 31.3, 29.0, 29.0, 28.9, 28.8, 28.7, 28.5, 25.8, 22.1, 22.0, 13.9 ppm.

31 [TC$_4$MA][3,4-DCOAc]

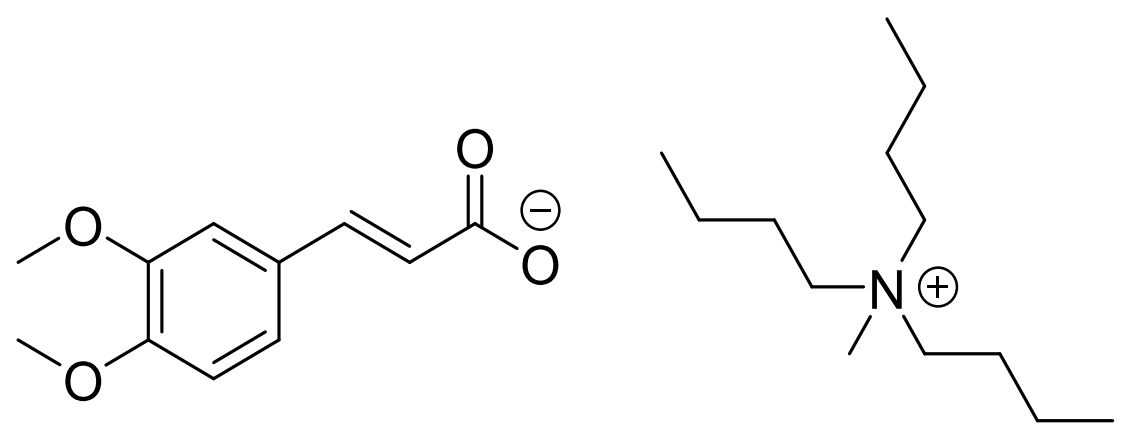

^{1}H-NMR (399.78 MHz, DMSO-d6) δ = 7.09 (s, 1H), 7.02 (d, *J* = 15.8 Hz, 1H), 6.96 (d, *J* = 8.26 Hz, 1H), 6.89 (d, *J* = 8.26 Hz, 1H), 6.31 (d, *J* = 15.8 Hz, 1H), 3.77 (s, 3H), 3.75 (s, 3H), 3.20 (m, 6H), 2.95 (s, 3H), 1.59 (m, 6H), 1.30 (m, 6H), 0.93 (t, *J* = 7.36 Hz, 9H) ppm.

^{13}C NMR (100.53 MHz, DMSO-d6) δ = 169.6, 149.4, 135.9, 130.3, 128.6, 120.8, 112.3, 110.2, 61.0, 56.5, 24.0, 19.8, 14.0 ppm.

32 [TC$_8$MA][3,4-DCOAc]

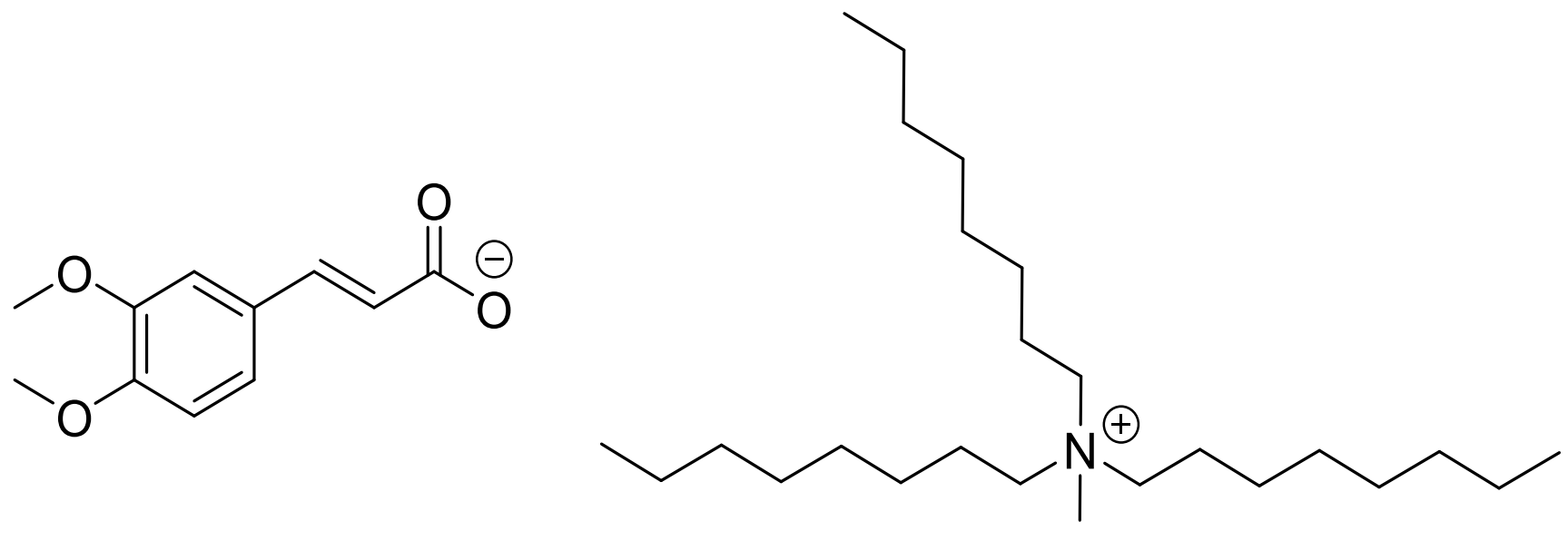

^{1}H-NMR (399.78 MHz, DMSO-d6) δ = 7.05 (s, 1H), 6.92 (m, 3H), 6.24 (d, *J* = 15.8 Hz, 1H), 3.77 (s, 3H), 3.74 (s. 3H), 3.20 (m, 6H), 2.95 (s, 3H), 1.60 (s, 6H), 1.26 (m, 30H), 0.86 (m, 9H) ppm.

^{13}C NMR (100.53 MHz, DMSO-d6) δ = 169.5, 149.4, 149.2, 134.6, 130.7, 130.3, 120.5, 112.3, 110.0, 61.0, 56.0, 31.7, 29.0, 28.9, 26.3, 22.6, 21.9, 14.5 ppm.

Table S3. Minimum Inhibitory Concentration (MIC) of salts on Bacteria

Nr.	Salts	MIC [in mg/L] Mean, [Minimum; Maximum]	
		E. coli	*L. monocytogenes*
**1**	[C_2mim][POAc]	3282, [2188; 4375]	4375, [4375; 4375]
**2**	[C_4mim][POAc]	3282, [2188; 4375]	4375, [4375; 4375]
**3**	[TMiPrA][POAc]	2735, [1095; 4375]	> 4375, [> 4375; > 4375]
**4**	[TMC_4A][POAc]	> 4375, [> 4375; > 4375]	> 4375, [> 4375; > 4375]
**5**	[TMC_8A][POAc]	2188, [2188; 2188]	729, [547; 1094]
**6**	[$TMC_{16}A$][POAc]	19, [17; 27]	2, [2; 2]
**7**	[TC_4MA][POAc]	4375, [4375; 4375]	4375, [4375; 4375]
**8**	[TC_8MA][POAc]	18, [2; 27]	8.5, [3; 14]
**9**	[C_2mim][3,4-MPOAc]	> 4375, [> 4375; > 4375]	4375, [4375; 4375]
**10**	[C_4mim][3,4-MPOAc]	4375, [4375; 4375]	4375, [4375; 4375]
**11**	[TMiPrA][3,4-MPOAc]	> 4375, [> 4375; > 4375]	> 4375, [> 4375; > 4375]
**12**	[TMC_4A][3,4-MPOAc]	2187, [1094; 4375]	3218, [1094; 4375]
**13**	[TMC_8A][3,4-MPOAc]	2917, [2188; 4375]	912, [547; 1094]
**14**	[$TMC_{16}A$][3,4-MPOAc]	10.5, [7; 14]	5.9, [3; 10.9]
**15**	[TC_4MA][3,4-MPOAc]	> 4375, [> 4375; > 4375]	4375, [4375; 4375]
**16**	[TC_8MA][3,4-MPOAc]	29.8, [14; 55]	2.1, [1; 3]
**17**	[C_2mim][3,4-MCOAc]	> 4375, [> 4375; > 4375]	4375, [4375; 4375]
**18**	[C_4mim][3,4-MCOAc]	> 4375, [> 4375; > 4375]	4375, [4375; 4375]
**19**	[TMiPrA][3,4-MCOAc]	> 4375, [> 4375; > 4375]	> 4375, [> 4375; > 4375]
**20**	[TMC_4A][3,4-MCOAc]	> 4375, [> 4375; > 4375]	> 4375, [> 4375; > 4375]
**21**	[TMC_8A][3,4-MCOAc]	9, [9; 9]	<4, [<4; <4]
**22**	[$TMC_{16}A$][3,4-MCOAc]	13, [11; 14]	1.5, [1; 2]
**23**	[TC_4MA][3,4-MCOAc]	4375, [4375; 4375]	4375, [4375; 4375]
**24**	[TC_8MA][3,4-MCOAc]	9, [9; 9]	<4, [<4; <4]
**25**	[C_2mim][3,4-DCOAc]	> 4375, [> 4375; > 4375]	4375, [4375; 4375]
**26**	[C_4mim][3,4-DCOAc]	> 4375, [> 4375; > 4375]	4375, [4375; 4375]
**27**	[TMiPrA][3,4-DCOAc]	> 4375, [> 4375; > 4375]	> 4375, [> 4375; > 4375]
**28**	[TMC_4A][3,4-DCOAc]	> 4375, [> 4375; > 4375]	4375, [4375; 4375]
**29**	[TMC_8A][3,4-DCOAc]	2188, [2188; 2188]	683, [547; 1094]
**30**	[$TMC_{16}A$][3,4-DCOAc]	65, [2; 273]	2, [2; 2]
**31**	[TC_4MA][3,4-DCOAc]	> 4375, [> 4375; > 4375]	> 4375, [> 4375; > 4375]
**32**	[TC_8MA][3,4-DCOAc]	11, [1; 27]	5, [3; 9]

Table S4. Minimum Inhibitory Concentration (MIC) and Non Inhibitory Concentration (NIC) of piperonylic acid analogues salts on PCR Enzyme Inhibition

Nr.	Salts	PCR inhibition			
		MIC (Mean)	95 % CI	NIC (Mean)	95 % CI
1	[C_2mim][POAc]	50000	0	2000	0
2	[C_4mim][POAc]	12500	0	1600	0
3	[TMiPrA][POAc]	2000	0	400	0
4	[TMC_4A][POAc]	6300	0	1600	0
5	[TMC_8A][POAc]	6300	0	1600	0
6	[TMC_{16}A][POAc]	240	313	24	15
7	[TC_4MA][POAc]	10000	0	400	0
8	[TC_8MA][POAc]	2000	0	80	0
9	[C_2mim][3,4-MPOAc]	10000	0	400	0
10	[C_4mim][3,4-MPOAc]	20000	0	400	0
11	[TMiPrA][3,4-MPOAc]	5500	6860	2366	3854
12	[TMC_4A][3,4-MPOAc]	10000	0	2000	0
13	[TMC_8A][3,4-MPOAc]	12500	0	1600	0
14	[TMC_{16}A][3,4-MPOAc]	186	209	24	15,6
15	[TC_4MA][3,4-MPOAc]	25000	0	3100	0
16	[TC_8MA][3,4-MPOAc]	80	0	16	0
17	[C_2mim][3,4-MCOAc]	10000	0	2000	0
18	[C_4mim][3,4-MCOAc]	10000	0	2000	0
19	[TMiPrA][3,4-MCOAc]	1200	1568	240	313
20	[TMC_4A][3,4-MCOAc]	2000	0	400	0
21	[TMC_8A][3,4-MCOAc]	2000	0	400	0
22	[TMC_{16}A][3,4-MCOAc]	240	313	9	12
23	[TC_4MA][3,4-MCOAc]	50000	0	2000	0
24	[TC_8MA][3,4-MCOAc]	10000	0	240	313
25	[C_2mim][3,4-DCOAc]	10000	0	400	0
26	[C_4mim][3,4-DCOAc]	50000	0	2000	0
27	[TMiPrA][3,4-DCOAc]	50000	0	2000	0
28	[TMC_4A][3,4-DCOAc]	50000	0	2000	0
29	[TMC_8A][3,4-DCOAc]	50000	0	2000	0
30	[TMC_{16}A][3,4-DCOAc]	186	209	48	62
31	[TC_4MA][3,4-DCOAc]	50000	0	2000	0
32	[TC_8MA][3,4-DCOAc]	2000	0	48	62

Table S5. Predicted biodegradation for piperonylic acid analogues salts

Nr.	Salts	Biodegradation	
		%Biodegradation	Classification
**R1**	[C_2mim][POAc]	16.04	NotEasily Biodegradable
**R2**	[C_4mim][POAc]	17.84	NotEasily Biodegradable
**R3**	[TMiPrA][POAc]	73.61	Easily Biodegradable
**R4**	[TMC_4A][POAc]	73.61	Easily Biodegradable
**R5**	[TMC_8A][POAc]	73.61	Easily Biodegradable
**R6**	[TMC_{16}A][POAc]	73.61	Easily Biodegradable
**R7**	[TC_4MA][POAc]	73.61	Easily Biodegradable
**R8**	[TC_8MA][POAc]	73.61	Easily Biodegradable
**R9**	[C_2mim][3,4-MPOAc]	19.01	NotEasily Biodegradable
**R10**	[C_4mim][3,4-MPOAc]	20.97	NotEasily Biodegradable
**R11**	[TMiPrA][3,4-MPOAc]	73.26	Easily Biodegradable
**R12**	[TMC_4A][3,4-MPOAc]	73.26	Easily Biodegradable
**R13**	[TMC_8A][3,4-MPOAc]	73.26	Easily Biodegradable
**R14**	[TMC_{16}A][3,4-MPOAc]	73.26	Easily Biodegradable
**R15**	[TC_4MA][3,4-MPOAc]	73.26	Easily Biodegradable
**R16**	[TC_8MA][3,4-MPOAc]	73.26	Easily Biodegradable
**R17**	[C_2mim][3,4-MCOAc]	16.52	NotEasily Biodegradable
**R18**	[C_4mim][3,4-MCOAc]	18.35	NotEasily Biodegradable
**R19**	[TMiPrA][3,4-MCOAc]	85.46	Easily Biodegradable
**R20**	[TMC_4A][3,4-MCOAc]	85.46	Easily Biodegradable
**R21**	[TMC_8A][3,4-MCOAc]	85.46	Easily Biodegradable
**R22**	[TMC_{16}A][3,4-MCOAc]	85.46	Easily Biodegradable
**R23**	[TC_4MA][3,4-MCOAc]	85.46	Easily Biodegradable
**R24**	[TC_8MA][3,4-MCOAc]	85.46	Easily Biodegradable
**R25**	[C_2mim][3,4-DCOAc]	23.79	NotEasily Biodegradable
**R26**	[C_4mim][3,4-DCOAc]	25.99	NotEasily Biodegradable
**R27**	[TMiPrA][3,4-DCOAc]	82.79	Easily Biodegradable
**R28**	[TMC_4A][3,4-DCOAc]	82.79	Easily Biodegradable
**R29**	[TMC_8A][3,4-DCOAc]	82.79	Easily Biodegradable
**R30**	[TMC_{16}A][3,4-DCOAc]	82.79	Easily Biodegradable
**R31**	[TC_4MA][3,4-DCOAc]	82.79	Easily Biodegradable
**R32**	[TC_8MA][3,4-DCOAc]	82.79	Easily Biodegradable

Table S6. ***In vitro*** **preliminary synergistic activity screening of piperonylic acid analogues and salts on** ***Blattella germanica***

Nr	Compound	GST Inhibition			EST Inhibition		
		*[EC_{50}]	±Std.Dev	#95 % CI	*[EC_{50}]	±Std.Dev	#95 % CI
	**PBO**	**42.8**	7.4	10.3	**18.5**	2	2.8
	**PA**	**5.40**	0.70	1.0	**80.6**	49.0	67.9
1	**[C_2mim][POAc]**	**58**	33	45.7	**71.8**	30.0	41.6
2	**[C_4mim][POAc]**	**67.5**	10	13.9	**44.1**	9	12.5
3	**[TMiPrA][POAc]**	**88.6**	23	31.9	**35**	20	27.7
4	**[TMC_4A][POAc]**	**89.7**	27	37.4	**72.7**	60	83.2
5	**[TMC_8A][POAc]**	**98.7**	27	37.4	**32.7**	28	38.8
6	**[TMC_{16}A][POAc]**	**4.3**	1.8	2.5	**0.4**	n.d	n.d
7	**[TC_4MA][POAc]**	**71.6**	5	6.9	**22**	15	20.8
8	**[TC_8MA][POAc]**	**1.8**	0.4	0.6	**0.4**	0.3	0.4
	**3,4-MPA**	**7.05**	1.72	2.4	**59.0**	7.0	9.7
9	**[C_2mim][3,4-MPOAc]**	**52.5**	11.3	15.7	**14.3**	9.0	12.5
10	**[C_4mim][3,4-MPOAc]**	**44.1**	7.2	10.0	**17.2**	6.0	8.3
11	**[TMiPrA][3,4-MPOAc]**	**51.4**	0.4	0.6	**19.2**	14.0	19.4
12	**[TMC_4A][3,4-MPOAc]**	**55.2**	1.1	1.5	**64.9**	28.0	38.8
13	**[TMC_8A][3,4-MPOAc]**	**85.0**	5.0	6.9	**13.2**	n.d	n.d
14	**[TMC_{16}A][3,4-MPOAc]**	**3.2**	0.2	0.3	**0.4**	0.1	0.1
15	**[TC_4MA][3,4-MPOAc]**	**47.7**	12.6	17.5	**23.6**	4.0	5.5
16	**[TC_8MA][3,4-MPOAc]**	**4.9**	3.5	4.9	**0.5**	0.1	0.1
	**3,4-MCA**	**11.86**	3.05	4.2	**23.20**	n.d	n.d
17	**[C_2mim][3,4-MCOAc]**	**6.9**	1.7	2.4	**97.5**	12.0	16.6
18	**[C_4mim][3,4-MCOAc]**	**8.1**	2.9	4.0	**39.3**	n.d	n.d
19	**[TMiPrA][3,4-MCOAc]**	**7.5**	0.3	0.4	**102.4**	19.0	26.3
20	**[TMC_4A][3,4-MCOAc]**	**12.7**	2.3	3.1	**81.6**	43.0	59.6
21	**[TMC_8A][3,4-MCOAc]**	**18.0**	1.3	1.7	**61.5**	38.0	52.7
22	**[TMC_{16}A][3,4-MCOAc]**	**2.1**	0.4	0.5	**0.5**	n.d	n.d
23	**[TC_4MA][3,4-MCOAc]**	**5.5**	3.2	4.5	**18.7**	n.d	n.d
24	**[TC_8MA][3,4-MCOAc]**	**1.7**	0.4	0.5	**0.5**	n.d	n.d
	**3,4-DCA**	**11.75**	9.08	12.6	**21.50**	n.d	n.d
25	**[C_2mim][3,4-DCOAc]**	**5.3**	1.7	2.4	**22.5**	18.0	n.d
26	**[C_4mim][3,4-DCOAc]**	**8.6**	0.2	0.3	**27.1**	5.0	6.9
27	**[TMiPrA][3,4-DCOAc]**	**5.8**	1.2	1.7	**22.0**	17.0	23.6
28	**[TMC_4A][3,4-DCOAc]**	**5.1**	2.0	2.8	**42.2**	39.0	54.1
29	**[TMC_8A][3,4-DCOAc]**	**3.5**	0.4	0.6	**9.6**	3.0	4.2
30	**[TMC_{16}A][3,4-DCOAc]**	**3.9**	3.1	4.3	**0.4**	n.d	n.d
31	**[TC_4MA][3,4-DCOAc]**	**17.0**	0.2	0.3	**24.9**	4.0	5.5
32	**[TC_8MA][3,4-DCOAc]**	**3.2**	0.2	0.2	**0.4**	n.d	n.d

*[EC_{50}] is mean value in milli molar(mM) concentration obtained from two biological & technical replicates; ±Std.Dev is standard deviation of mean; #95 % CI is 95 % confidence intervals.

Table S7. Synergistic Activity of piperonylic acid analogues salts on *Blattella germanica*

Analogues & Salts	mM	[1]N	[2]n	*Salt(50 mg/mL) + PYR (0.5 mg/mL)								
				Knockdown (1 hr)			Mortality (24 hr)			Mortality (48 hr)		
				Mean	SD	CI	Mean	SD	CI	Mena	SD	CI
†PA	300	4	40	80%	20%	20%	40%	20%	20%	50%	20%	20%
1 [C_2mim][POAc]	180	3	30	60%	20%	23%	30%	20%	23%	40%	10%	11%
3 [TMiPrA][POAc]	187	3	30	40%	20%	23%	30%	10%	11%	20%	10%	11%
4 [TMC_4A][POAc]	177	4	40	80%	10%	10%	40%	20%	20%	40%	20%	20%
†3,4-MPA	277	4	40	80%	20%	20%	60%	30%	29%	60%	20%	20%
9 [C_2mim][3,4-MPOAc]	172	4	40	70%	30%	29%	50%	20%	20%	40%	10%	10%
11 [TMiPrA][3,4-MPOAc]	177	3	30	60%	30%	34%	20%	10%	11%	20%	10%	11%
12 [TMC_4A][3,4-MPOAc]	169	4	40	70%	20%	20%	20%	10%	10%	30%	10%	10%
†3,4-MCA	260	4	40	80%	20%	20%	40%	20%	20%	40%	10%	10%
17 [C_2mim][3,4-MCOAc]	165	3	30	60%	20%	23%	30%	20%	23%	30%	20%	23%
19 [TMiPrA][3,4-MCOAc]	170	3	30	70%	30%	34%	30%	10%	11%	20%	10%	11%
20 [TMC_4A][3,4-MCOAc]	162	4	40	70%	10%	10%	30%	20%	20%	30%	20%	20%
†3,4-DCA	240	4	40	80%	30%	29%	60%	30%	29%	60%	30%	29%
25 [C_2mim][3,4-DCOAc]	157	4	40	70%	30%	29%	40%	10%	10%	30%	10%	10%
27 [TMiPrA][3,4-DCOAc]	161	4	40	90%	10%	10%	30%	10%	10%	30%	10%	10%
28 [TMC_4A][3,4-DCOAc]	154	4	40	60%	20%	20%	40%	0%	0%	40%	0%	0%
PBO (10 mg/mL) + PYR (0.5 mg/mL)	30 + 15	19	190	60%	20%	9%	60%	20%	9%	60%	20%	9%
PYR (0.5 mg/mL) only	15	19	190	60%	20%	9%	30%	20%	9%	20%	20%	9%
Ethanol		2	20	0%	0%	0%	0%	0%	0%	0%	0%	0%
DMSO		2	20	0%	0%	0%	0%	0%	0%	0%	0%	0%

* Salts (50 mg/mL) + PYR (0.5 mg/mL) in Ethanol; †Free acids (50 mg/mL) in DMSO; [1]N = Number of Experiments; [2]n = Number of treated individuals; mM is milli molar concentration; SD is standard deviation; CI is % Confidence Interval; Ethanol & DMSO did not show any effect on mortality.

S8. NMR spectra for Piperonylic acid analogues salts

1 [C_2mim][POAc]

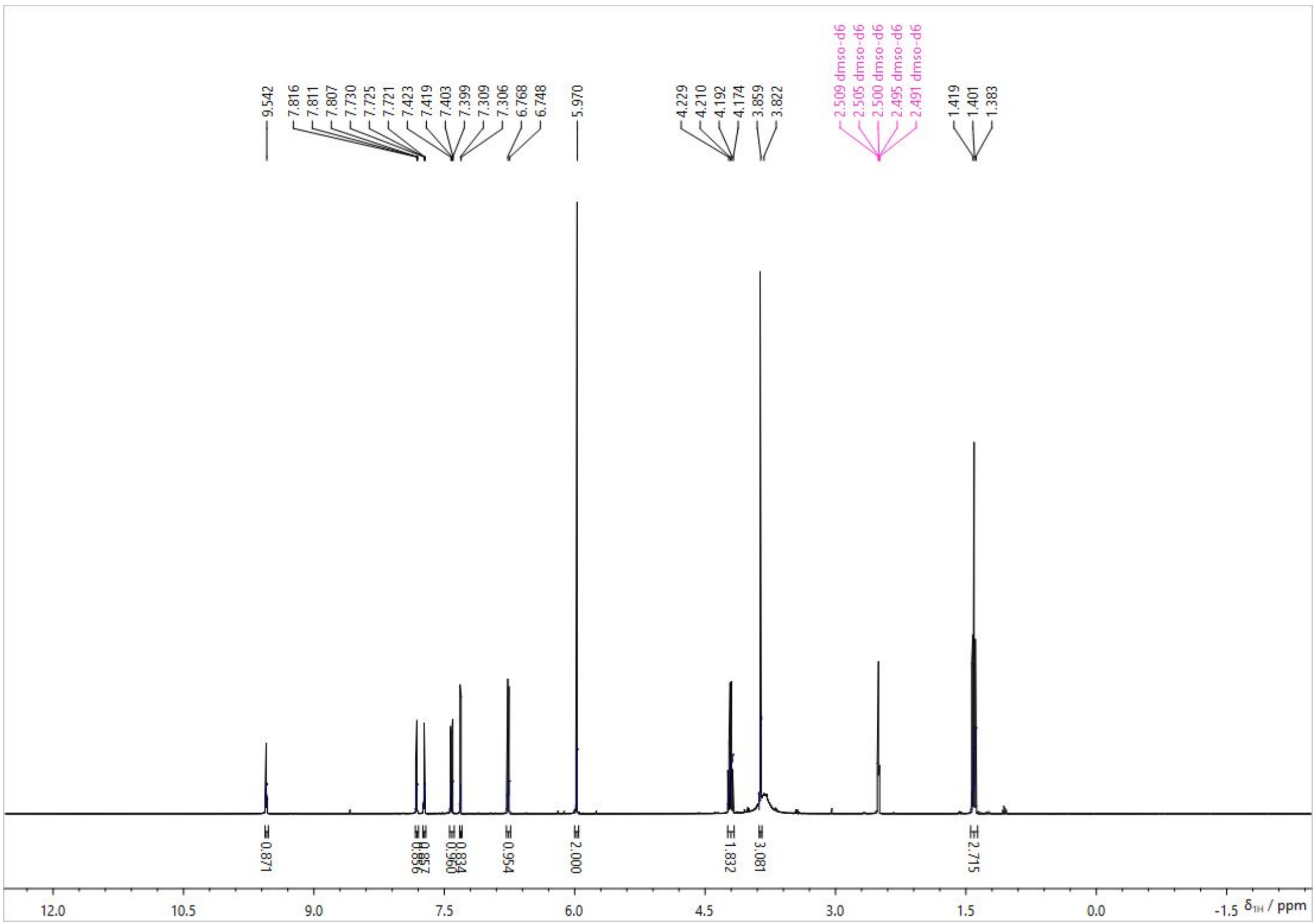

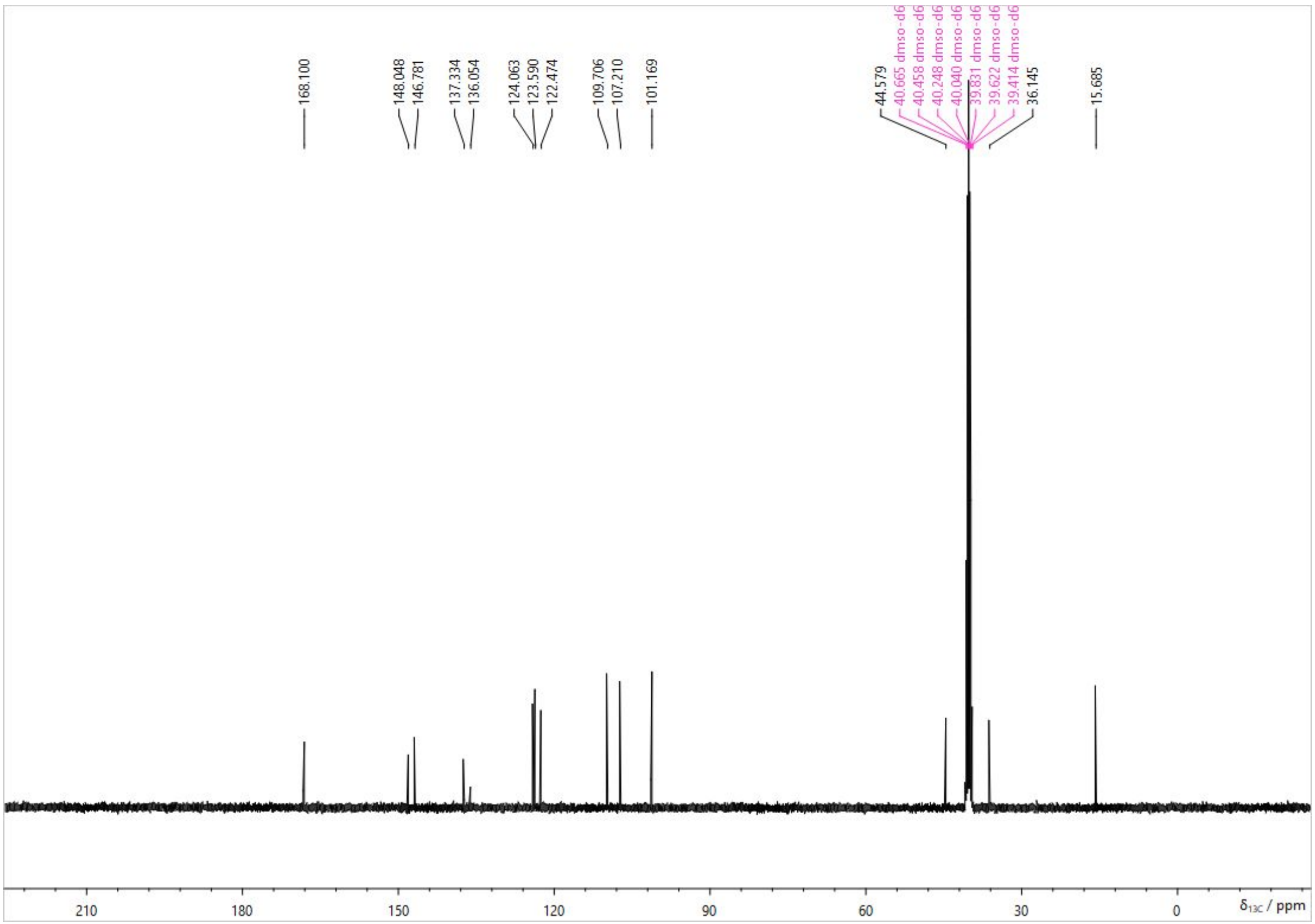
168.100
148.048
146.781
137.334
136.054
124.063
123.590
122.474
109.706
107.210
101.169
44.579
40.665 dmso-d6
40.458 dmso-d6
40.248 dmso-d6
40.040 dmso-d6
39.831 dmso-d6
39.622 dmso-d6
39.414 dmso-d6
36.145
15.685
210
180
150
120
90
60
30
0
δ13C / ppm

2 [C_4mim][POAc]

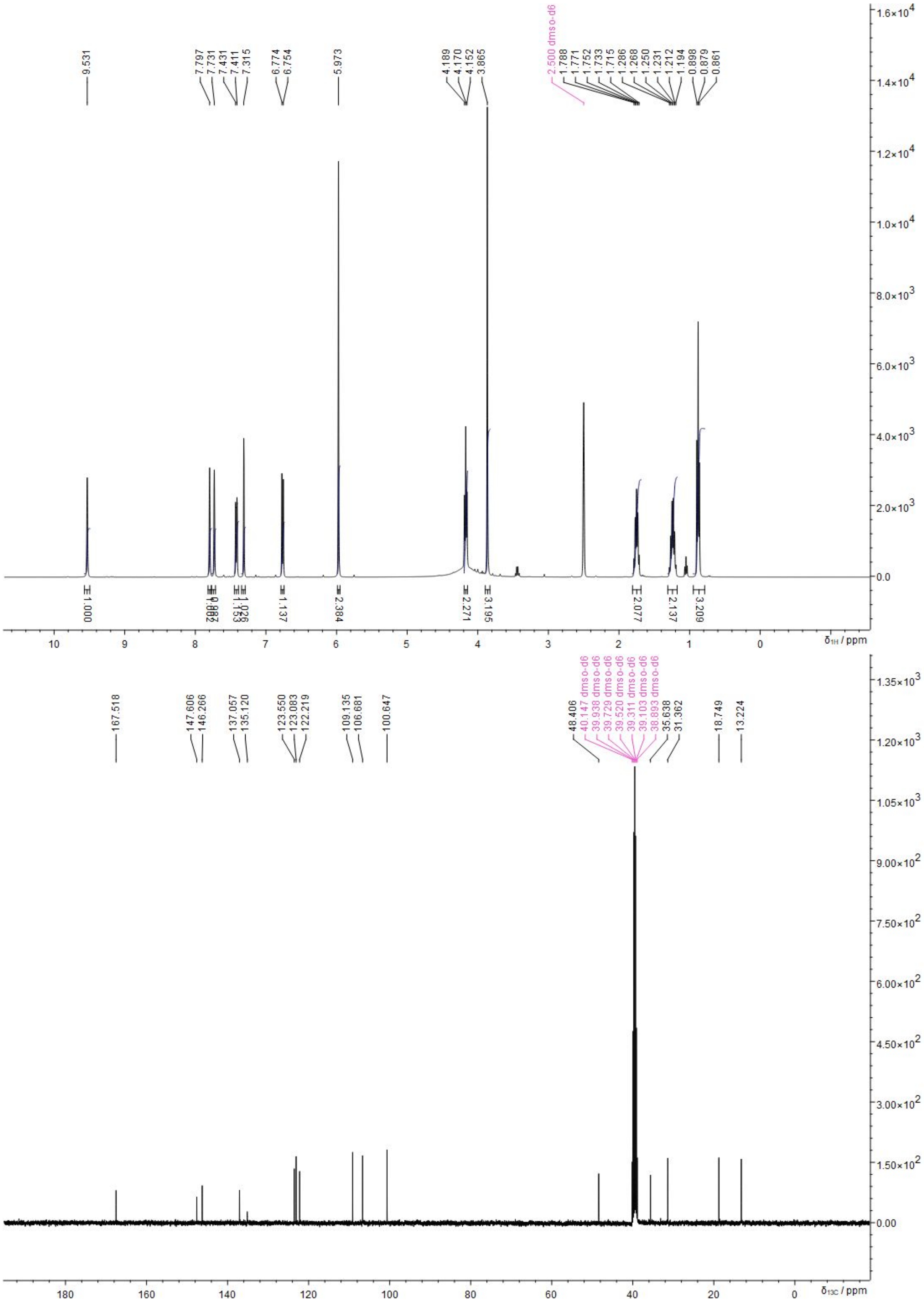
9.531
7.797
7.731
7.431
7.411
7.315
6.774
6.754
5.973
4.189
4.170
4.152
3.865
2.500 dmso-d6
1.788
1.771
1.752
1.733
1.715
1.286
1.268
1.250
1.231
1.212
1.194
0.898
0.879
0.861
1.000
1.137
2.384
2.271
3.195
2.077
2.137
3.209
δ1H / ppm
167.518
147.606
146.266
137.057
135.120
123.550
123.083
122.219
109.135
106.681
100.647
48.406
40.147 dmso-d6
39.938 dmso-d6
39.729 dmso-d6
39.520 dmso-d6
39.311 dmso-d6
39.103 dmso-d6
38.893 dmso-d6
35.638
31.362
18.749
13.224
δ13C / ppm

3 [TMiPrA][POAc]

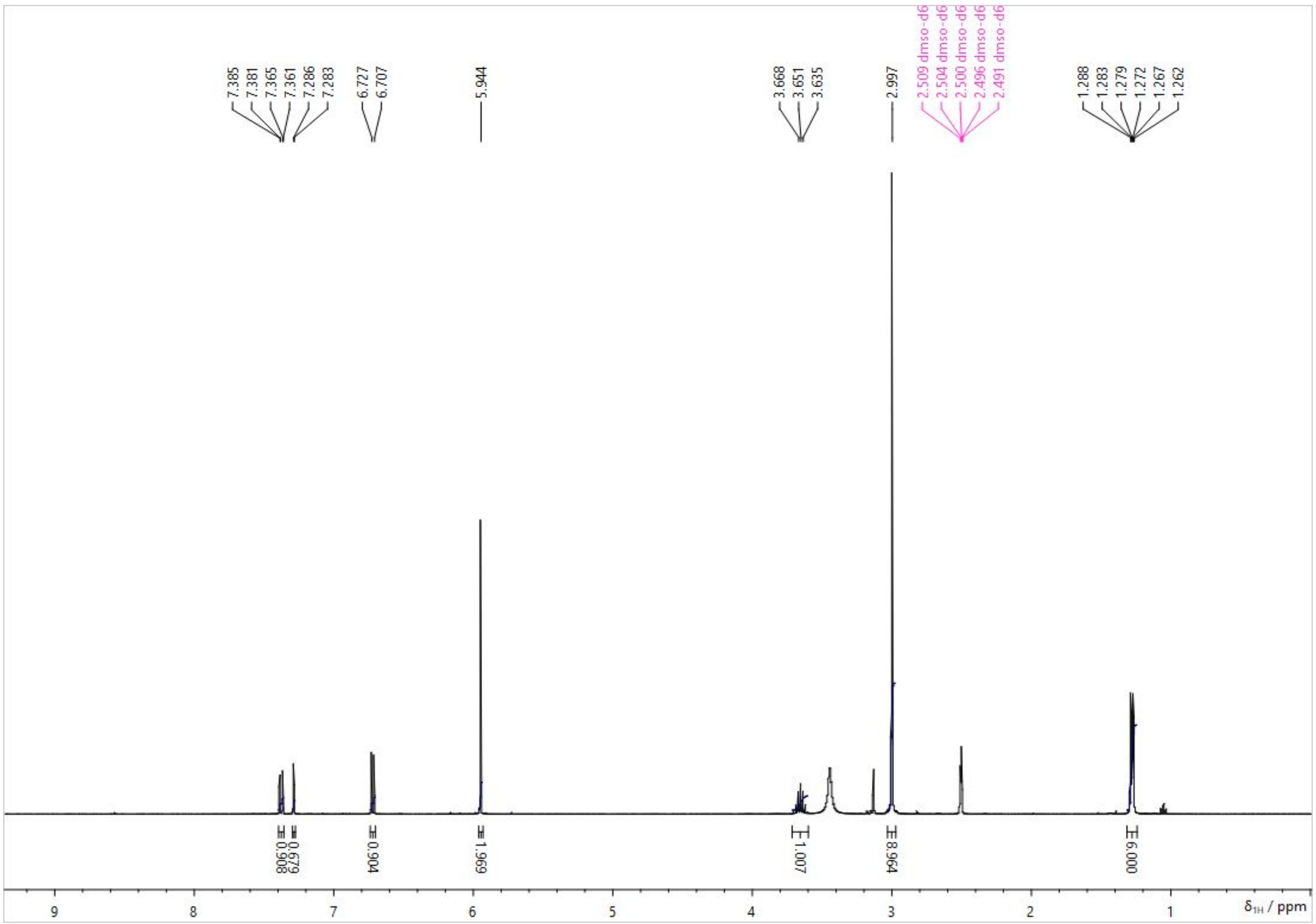

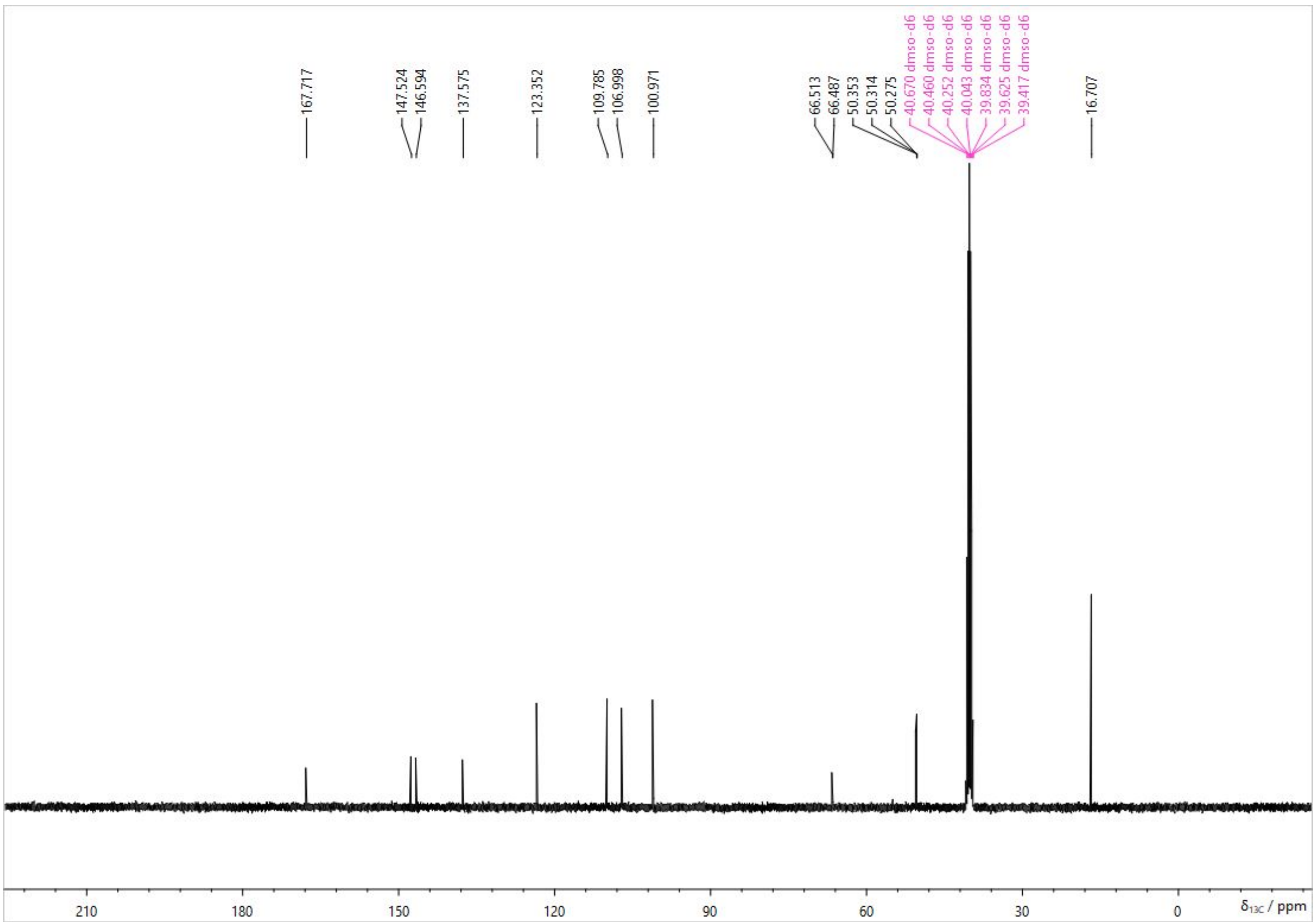

4 [TMC$_4$A][POAc]

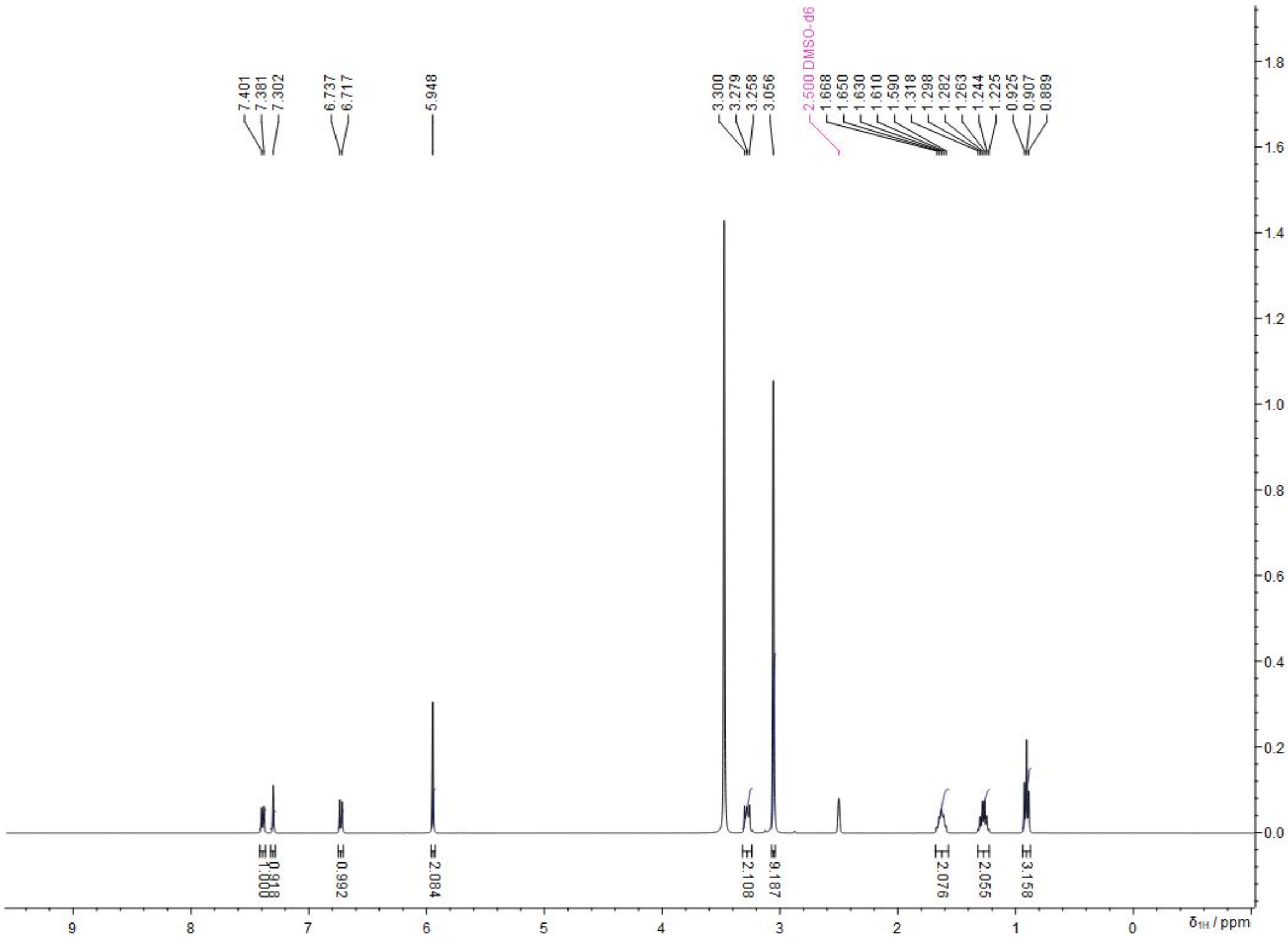
7.401
7.381
7.302
6.737
6.717
5.948
3.300
3.279
3.258
3.056
2.500 DMSO-d6
1.668
1.650
1.630
1.610
1.590
1.318
1.298
1.282
1.263
1.244
1.225
0.925
0.907
0.889
1.000
0.918
0.992
2.084
2.108
9.187
2.076
2.055
3.158
δ1H / ppm

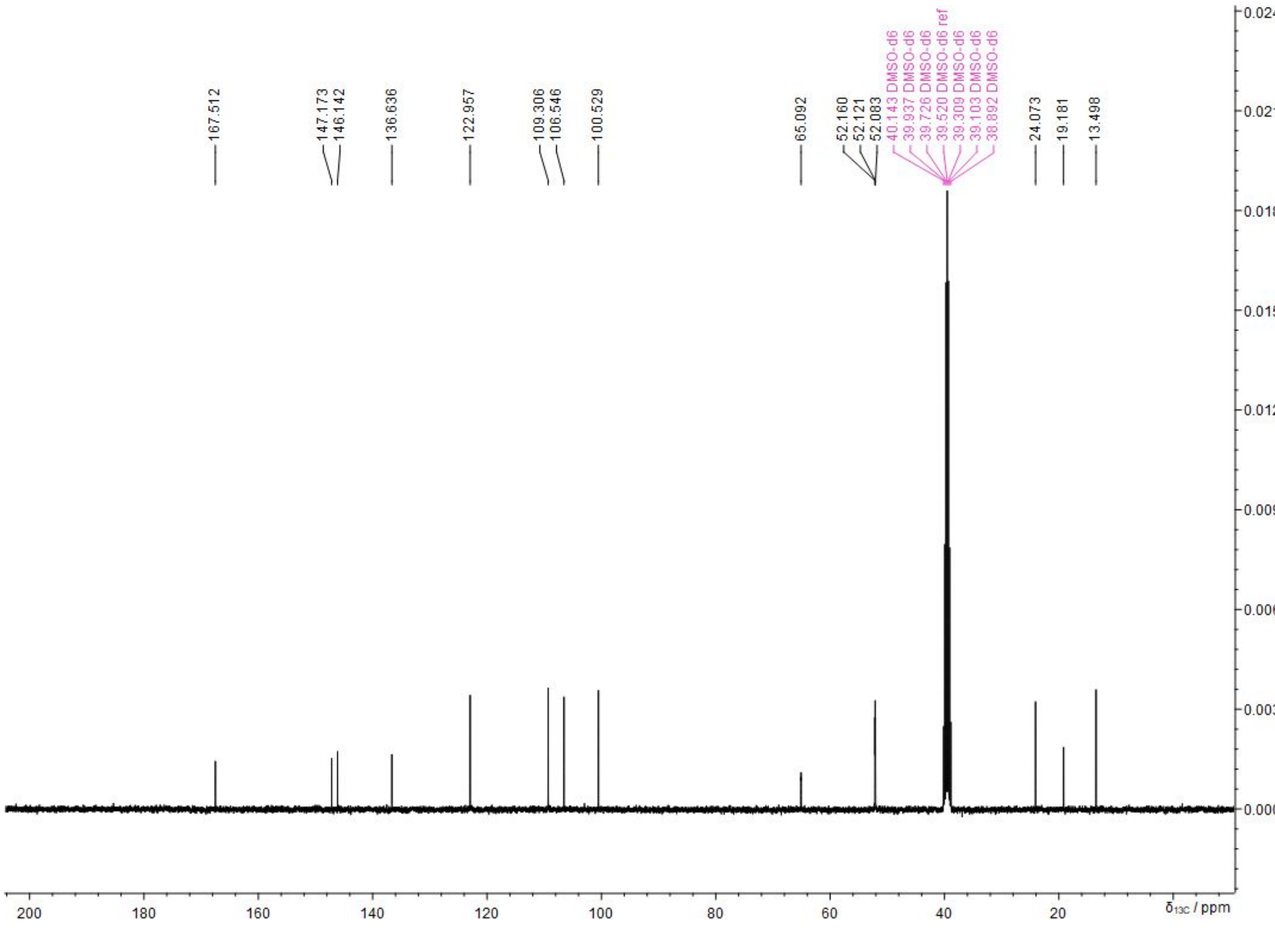
167.512
147.173
146.142
136.636
122.957
109.306
106.546
100.529
65.092
52.160
52.121
52.083
40.143 DMSO-d6
39.937 DMSO-d6
39.726 DMSO-d6
39.520 DMSO-d6 ref
39.309 DMSO-d6
39.103 DMSO-d6
38.892 DMSO-d6
24.073
19.181
13.498
δ13C / ppm

5 [TMC$_8$A][POAc]

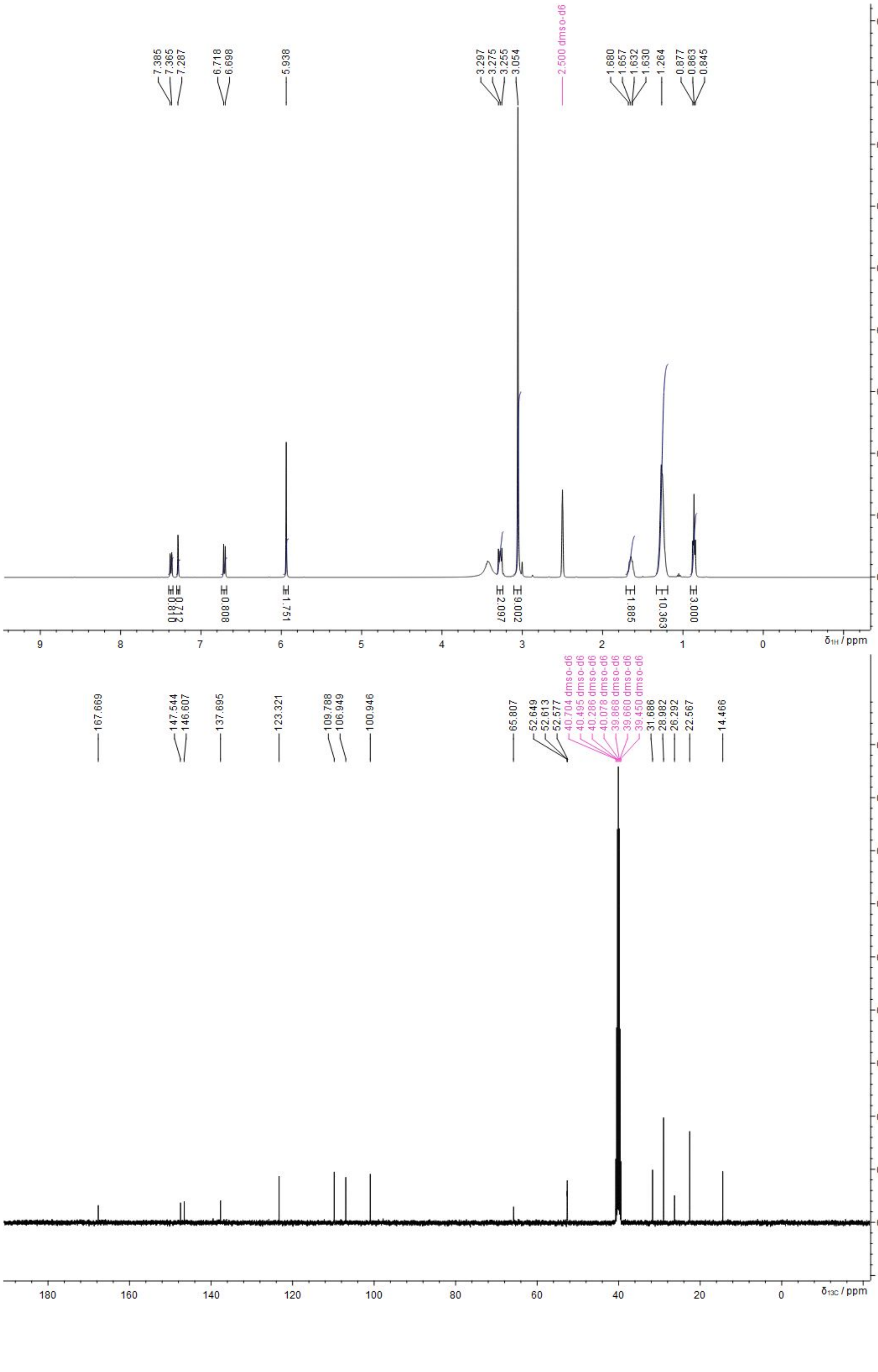

7.385
7.365
7.287
6.718
6.698
5.938
3.297
3.275
3.255
3.054
2.500 dmso-d6
1.680
1.657
1.632
1.630
1.264
0.877
0.863
0.845
0.810
0.712
0.808
1.751
2.097
9.002
1.885
10.363
3.000
δ1H / ppm
167.669
147.544
146.607
137.695
123.321
109.788
106.949
100.946
65.807
52.649
52.613
52.577
40.704 dmso-d6
40.495 dmso-d6
40.286 dmso-d6
40.078 dmso-d6
39.868 dmso-d6
39.660 dmso-d6
39.450 dmso-d6
31.686
28.982
26.292
22.567
14.466
δ13C / ppm

6 [TMC$_{16}$A][POAc]

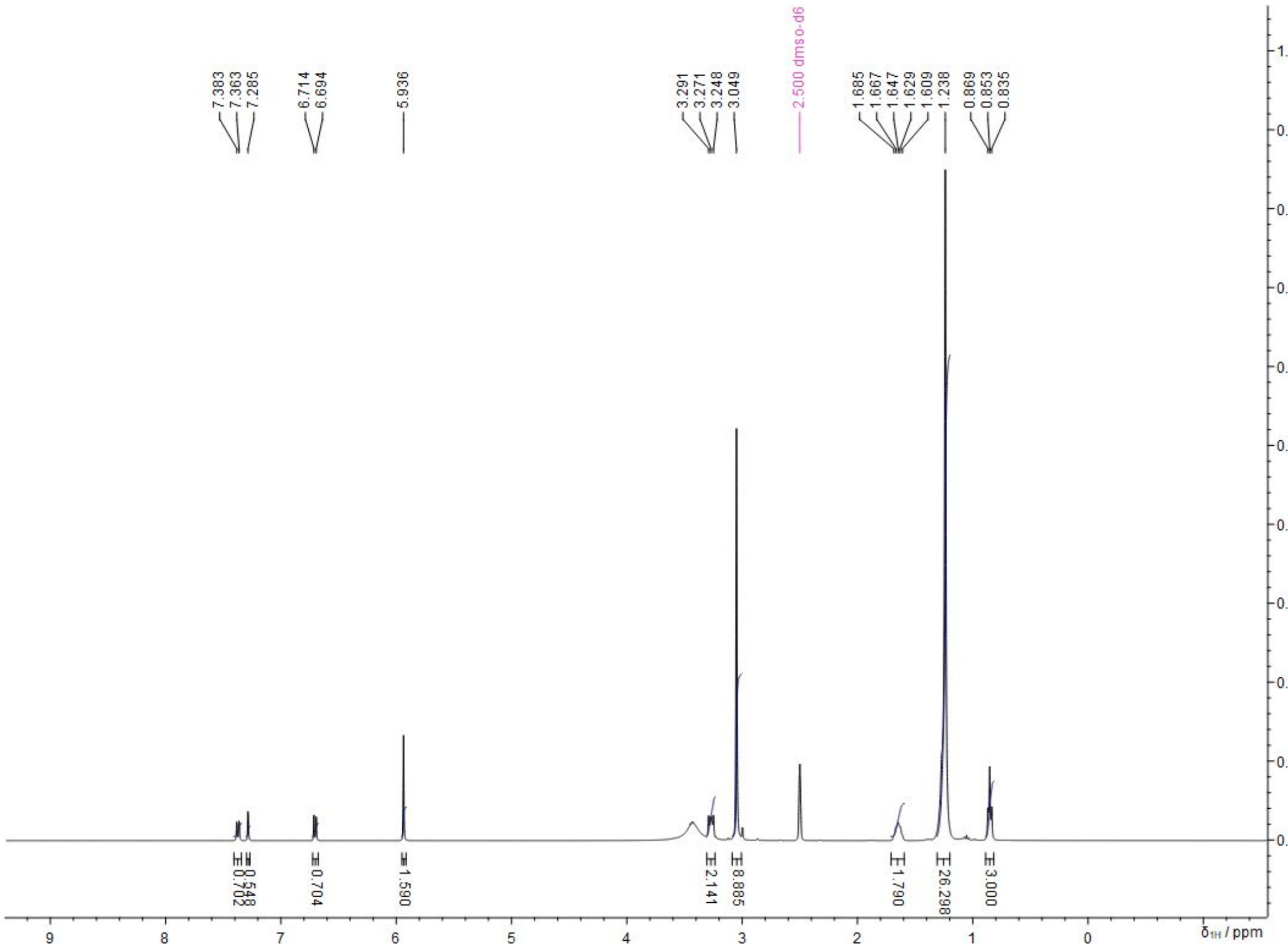

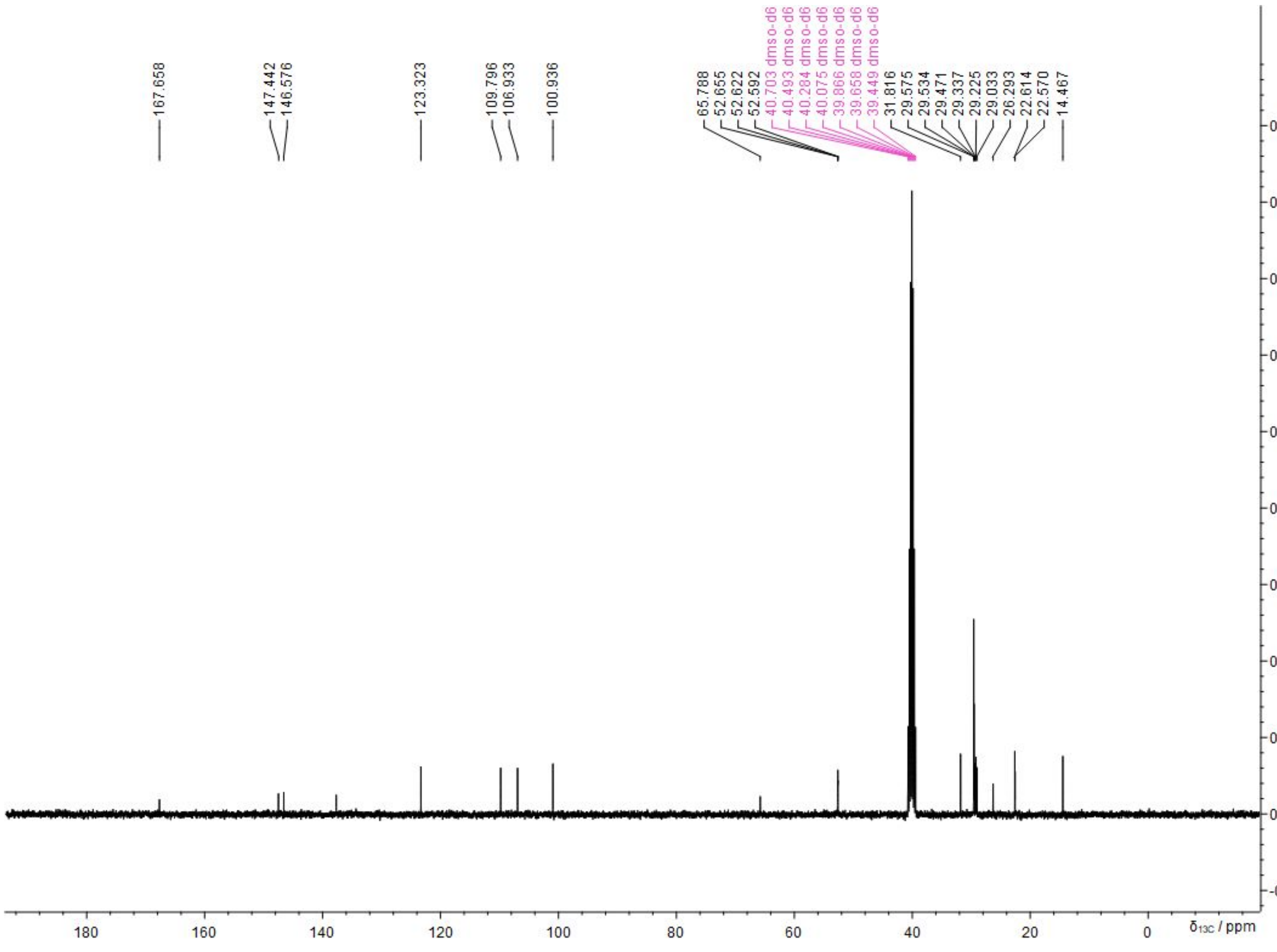

7 [TC_4MA][POAc]

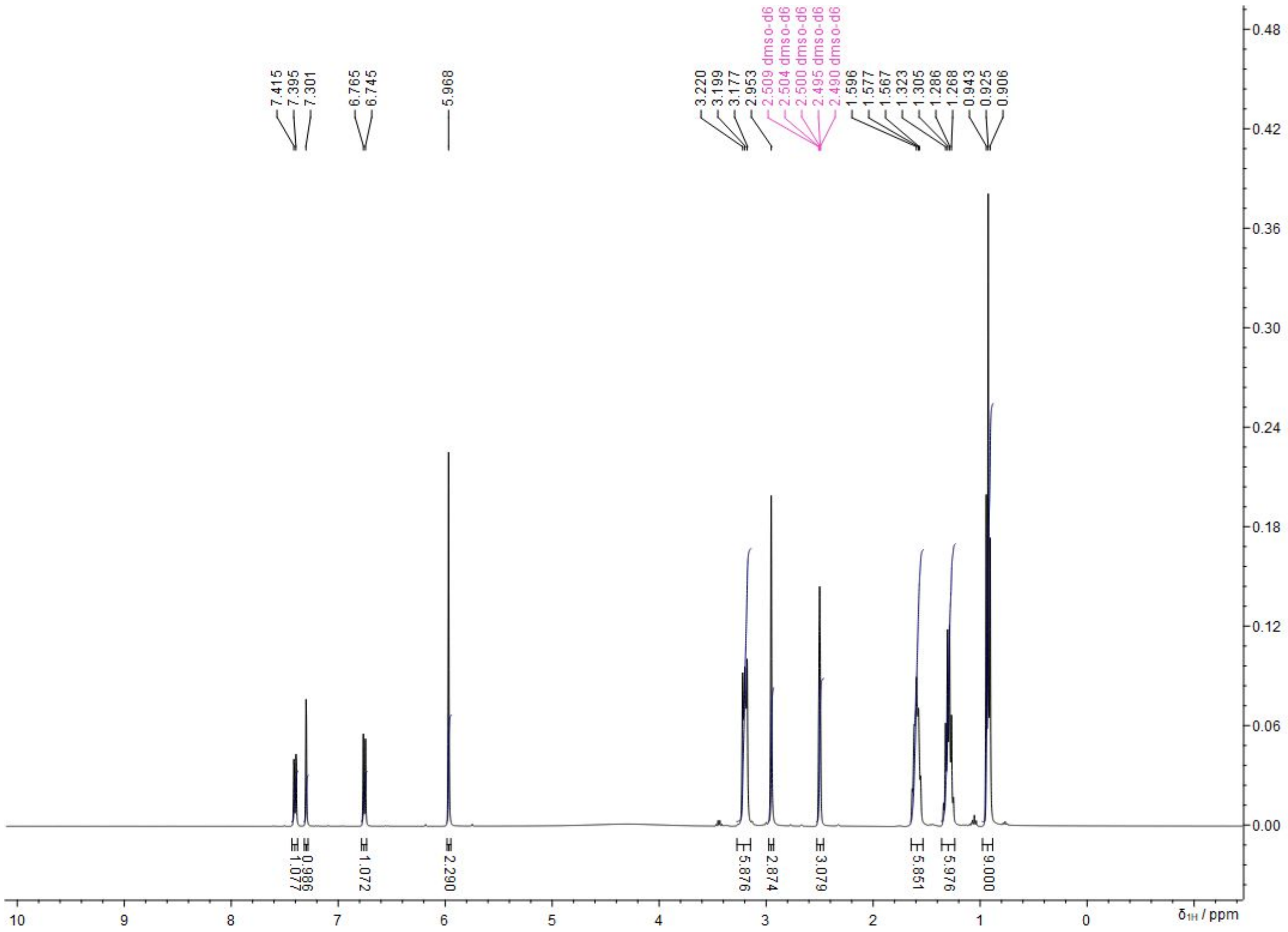
7.415
7.395
7.301
6.765
6.745
5.968
3.220
3.199
3.177
2.953
2.509 dmso-d6
2.504 dmso-d6
2.500 dmso-d6
2.495 dmso-d6
2.490 dmso-d6
1.596
1.577
1.567
1.323
1.305
1.286
1.268
0.943
0.925
0.906
1.077
0.986
1.072
2.290
5.876
2.874
3.079
5.851
5.976
9.000
δ1H / ppm

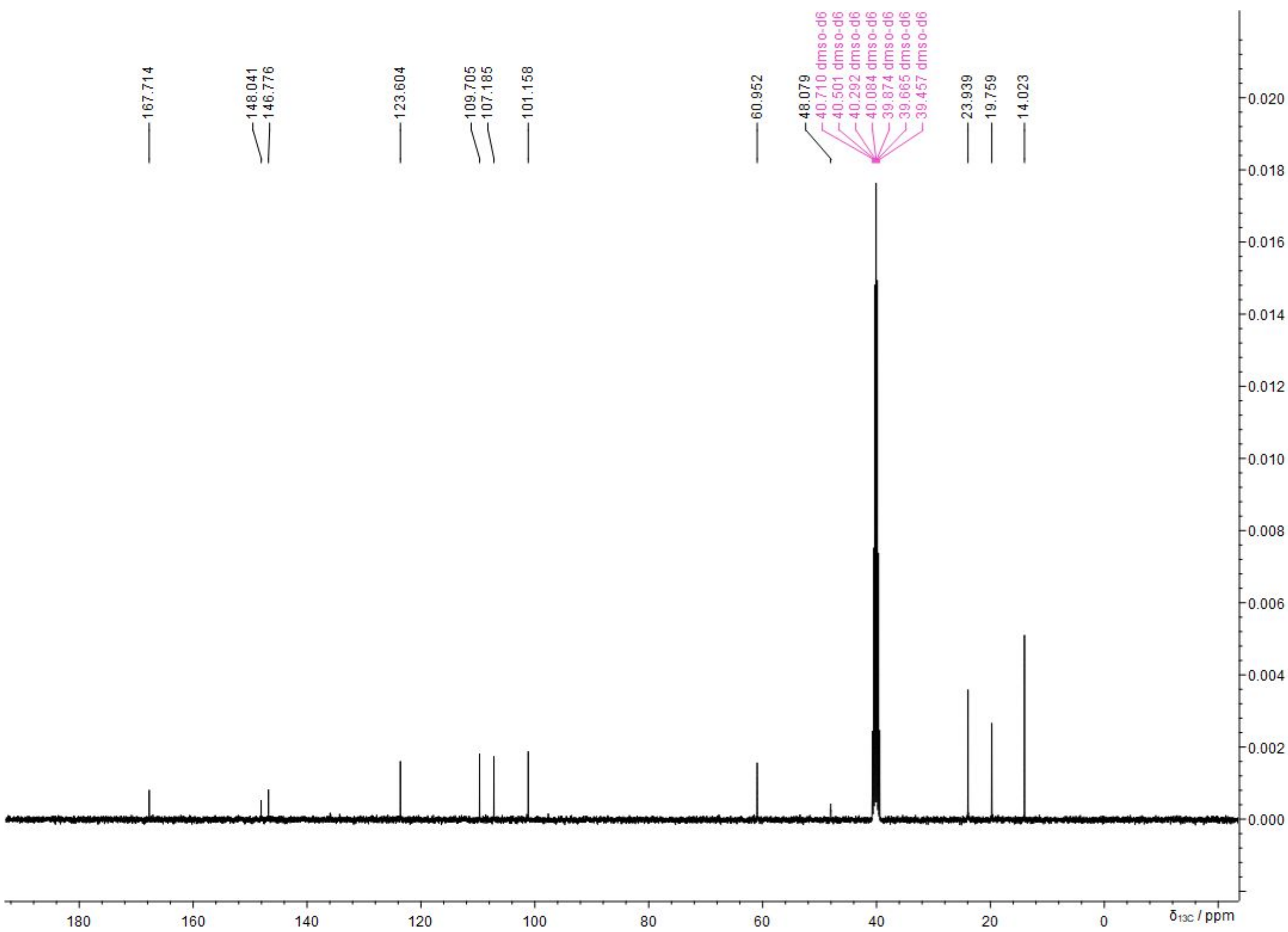
167.714
148.041
146.776
123.604
109.705
107.185
101.158
60.952
48.079
40.710 dmso-d6
40.501 dmso-d6
40.292 dmso-d6
40.084 dmso-d6
39.874 dmso-d6
39.665 dmso-d6
39.457 dmso-d6
23.939
19.759
14.023
δ13C / ppm

8 [TC$_8$MA][POAc]

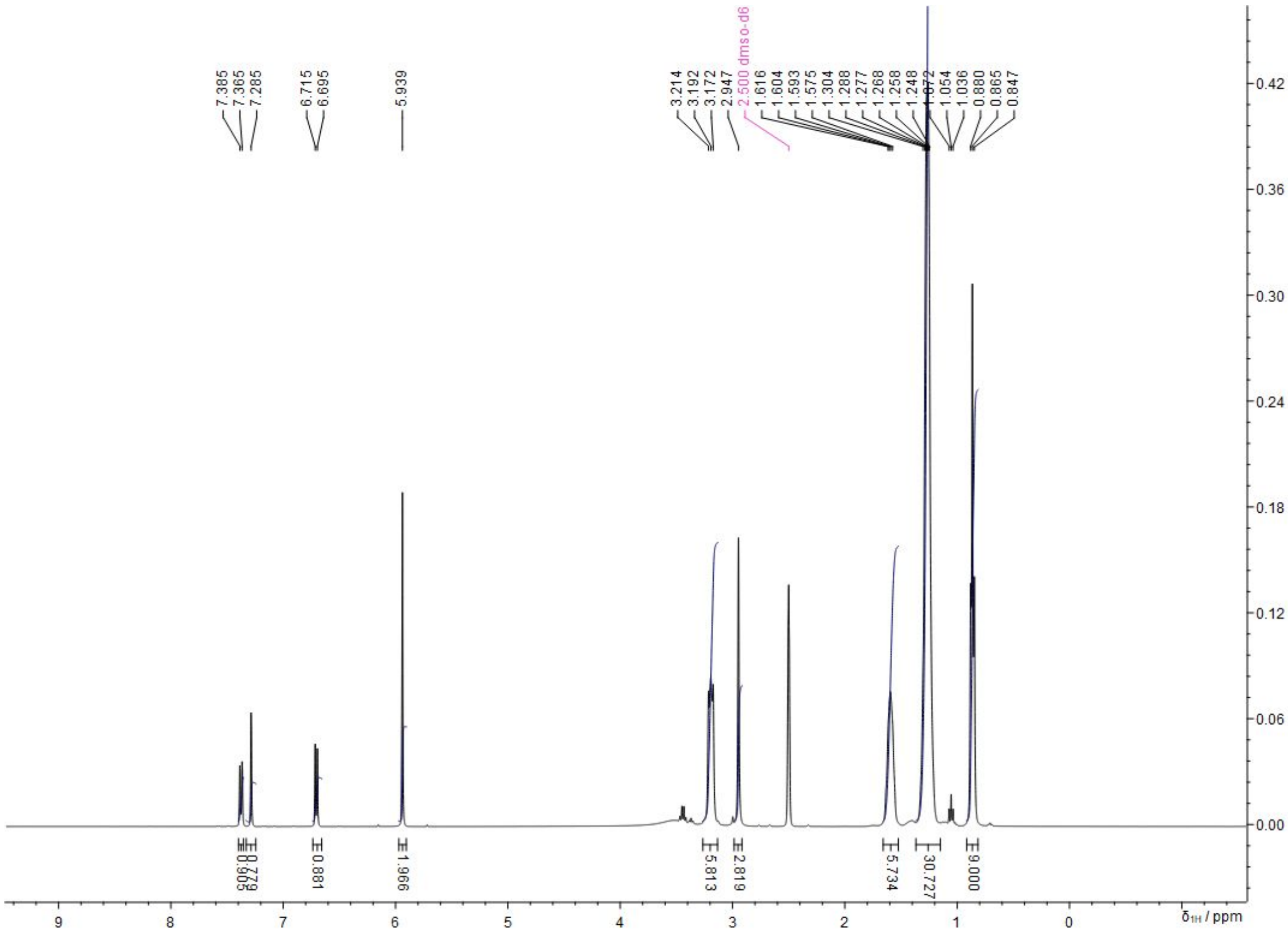
7.385
7.365
7.285
6.715
6.695
5.939
3.214
3.192
3.172
2.947
2.500 dmso-d6
1.616
1.604
1.593
1.575
1.304
1.288
1.277
1.268
1.258
1.248
1.072
1.054
1.036
0.880
0.865
0.847
0.905
0.779
0.881
1.966
5.813
2.819
5.734
30.727
9.000
δ1H / ppm

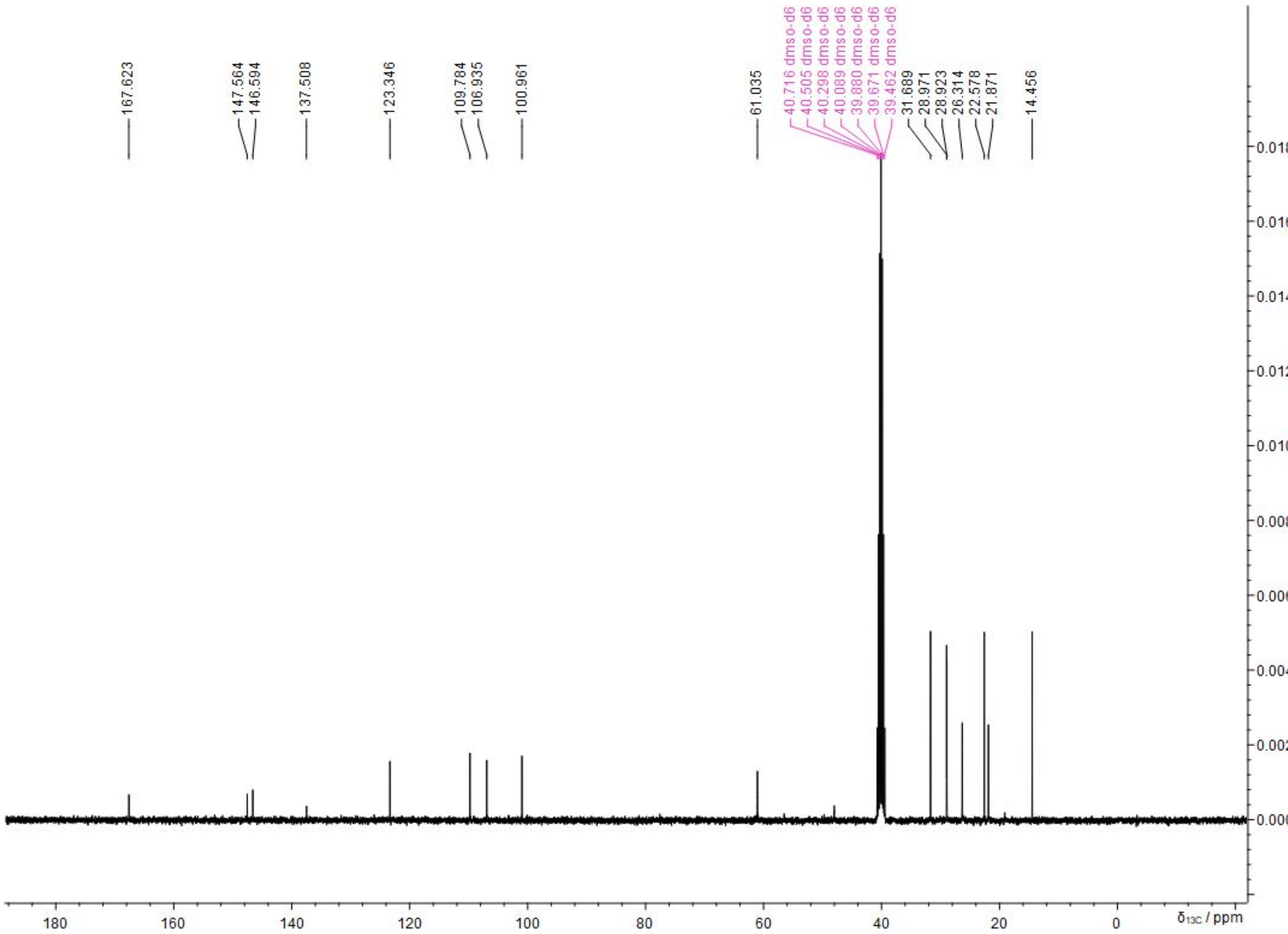
167.623
147.564
146.594
137.508
123.346
109.784
106.935
100.961
61.035
40.716 dmso-d6
40.505 dmso-d6
40.298 dmso-d6
40.089 dmso-d6
39.880 dmso-d6
39.671 dmso-d6
39.462 dmso-d6
31.689
28.971
28.923
26.314
22.578
21.871
14.456
δ13C / ppm

9 [C_2mim][3,4-MPOAc]

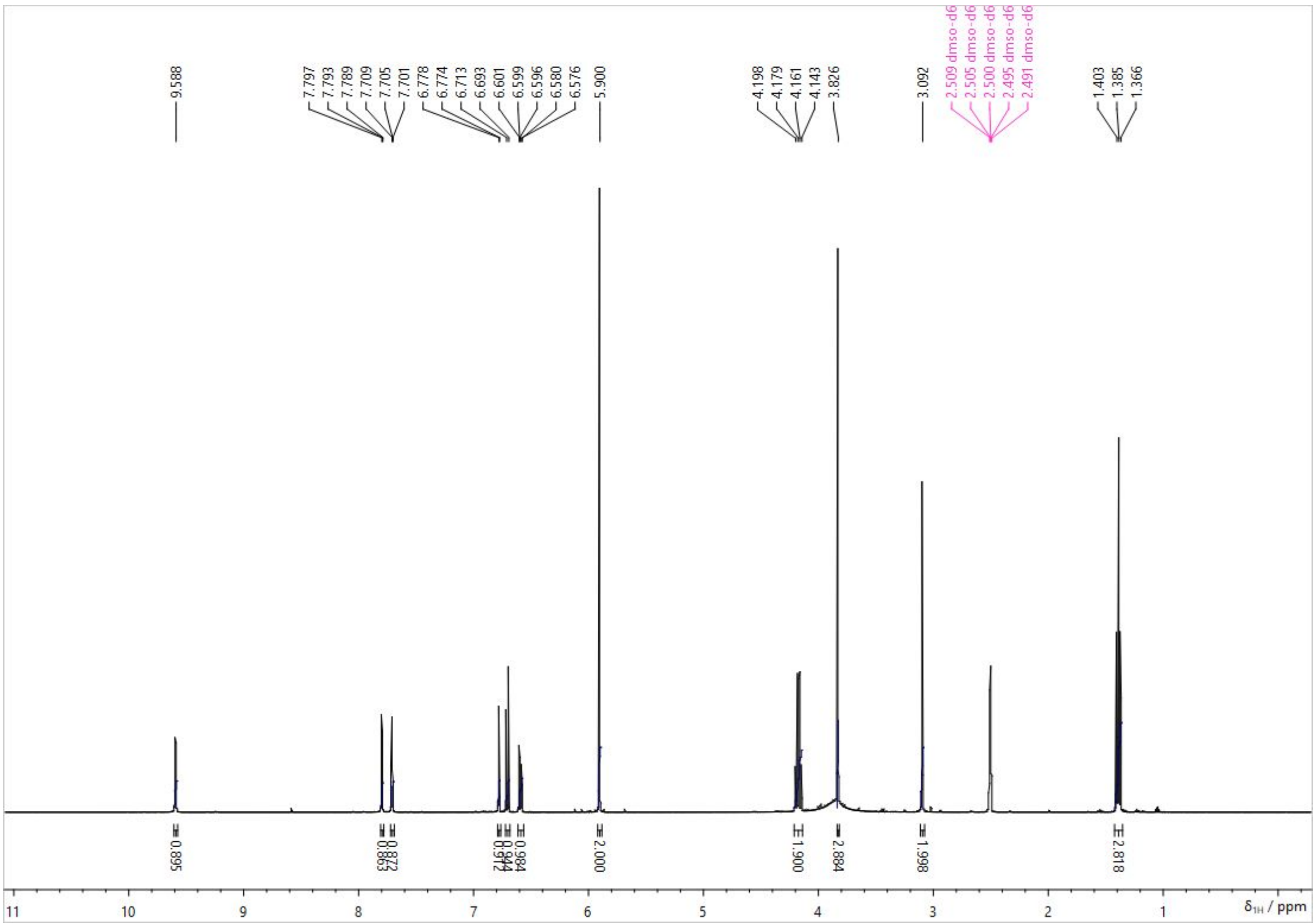

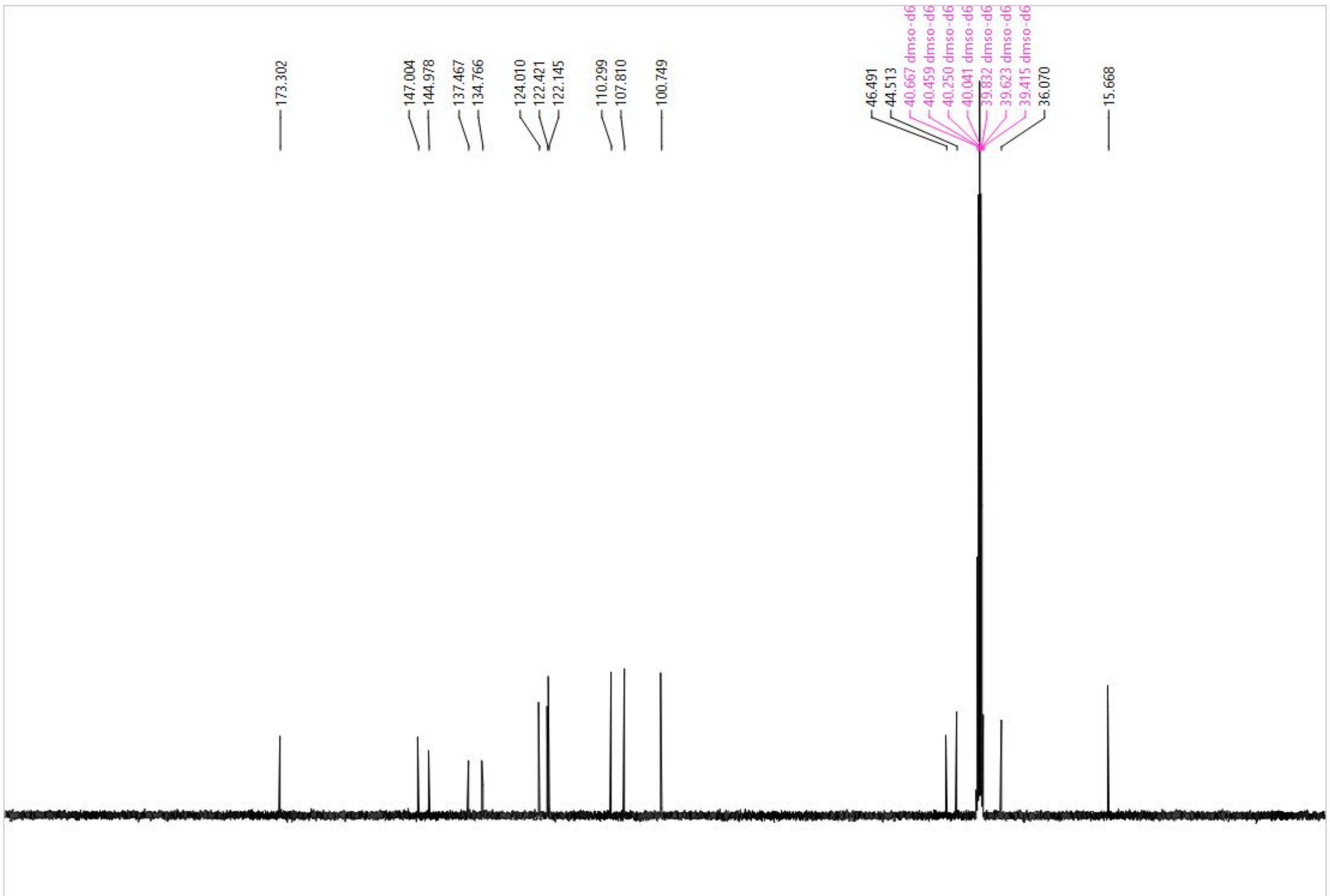

10 [C_4mim][3,4-MPOAc]

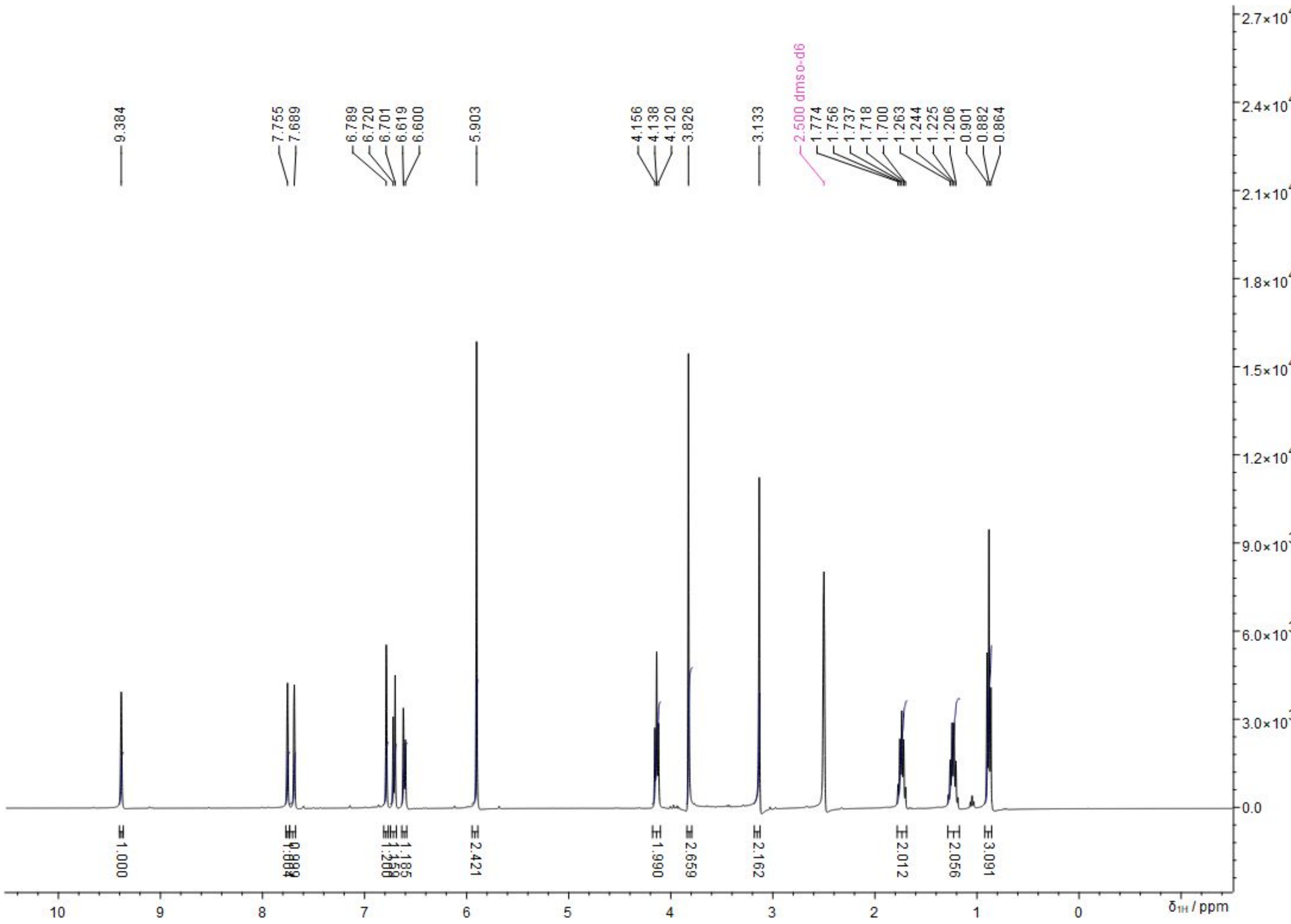

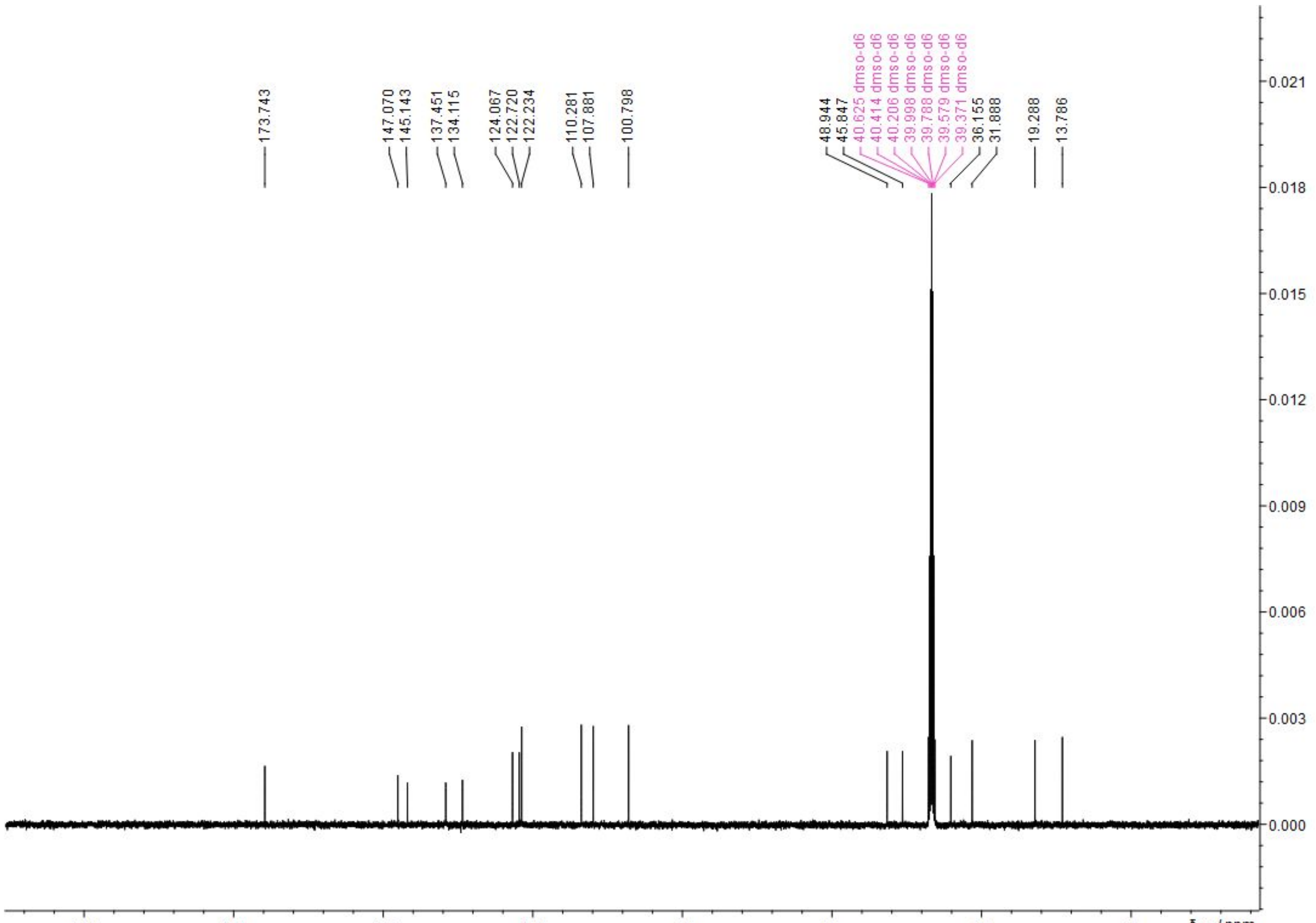

11 [TMiPrA][3,4-MPOAc]

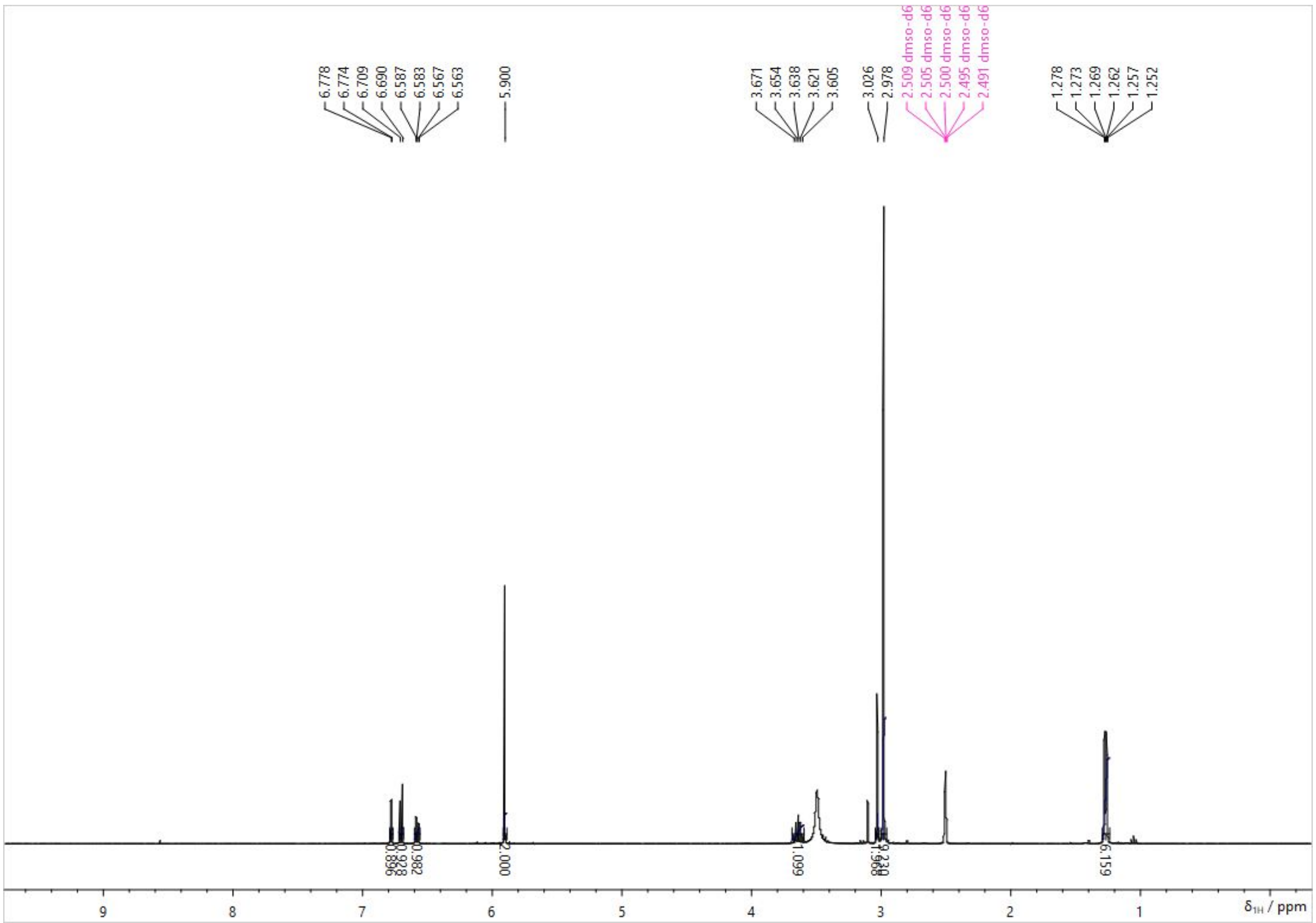

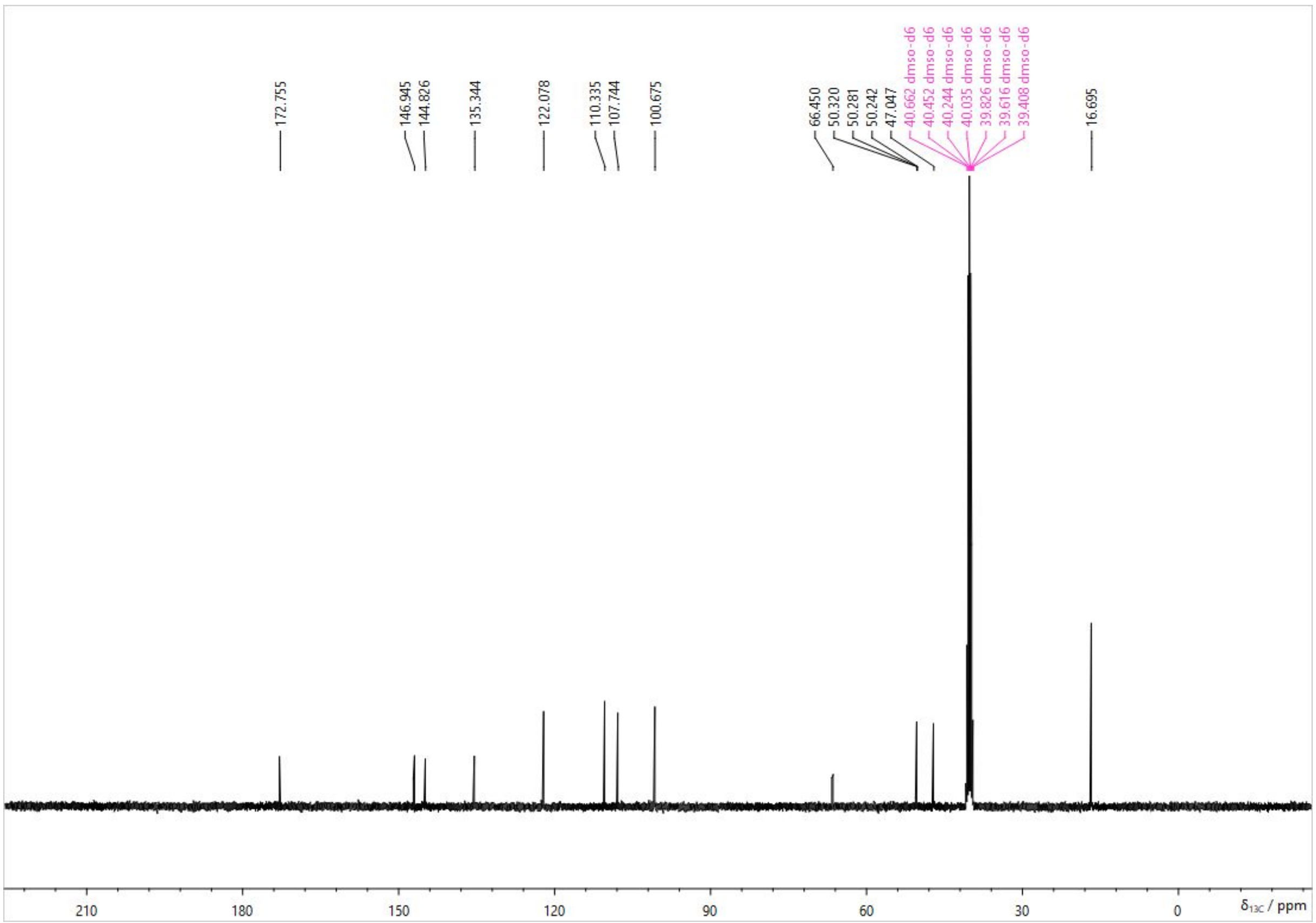

12 [TMC_4A][3,4-MPOAc]

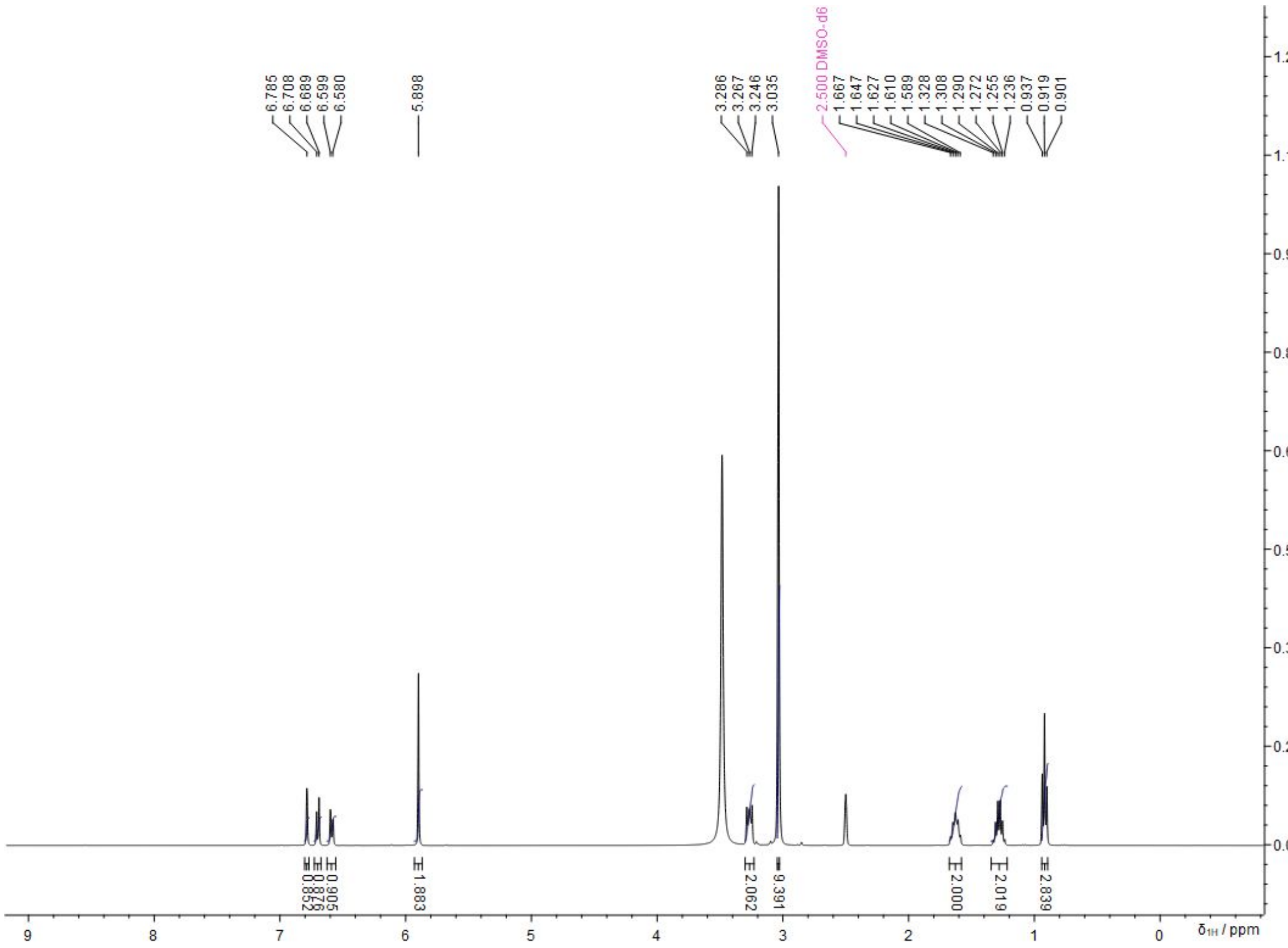

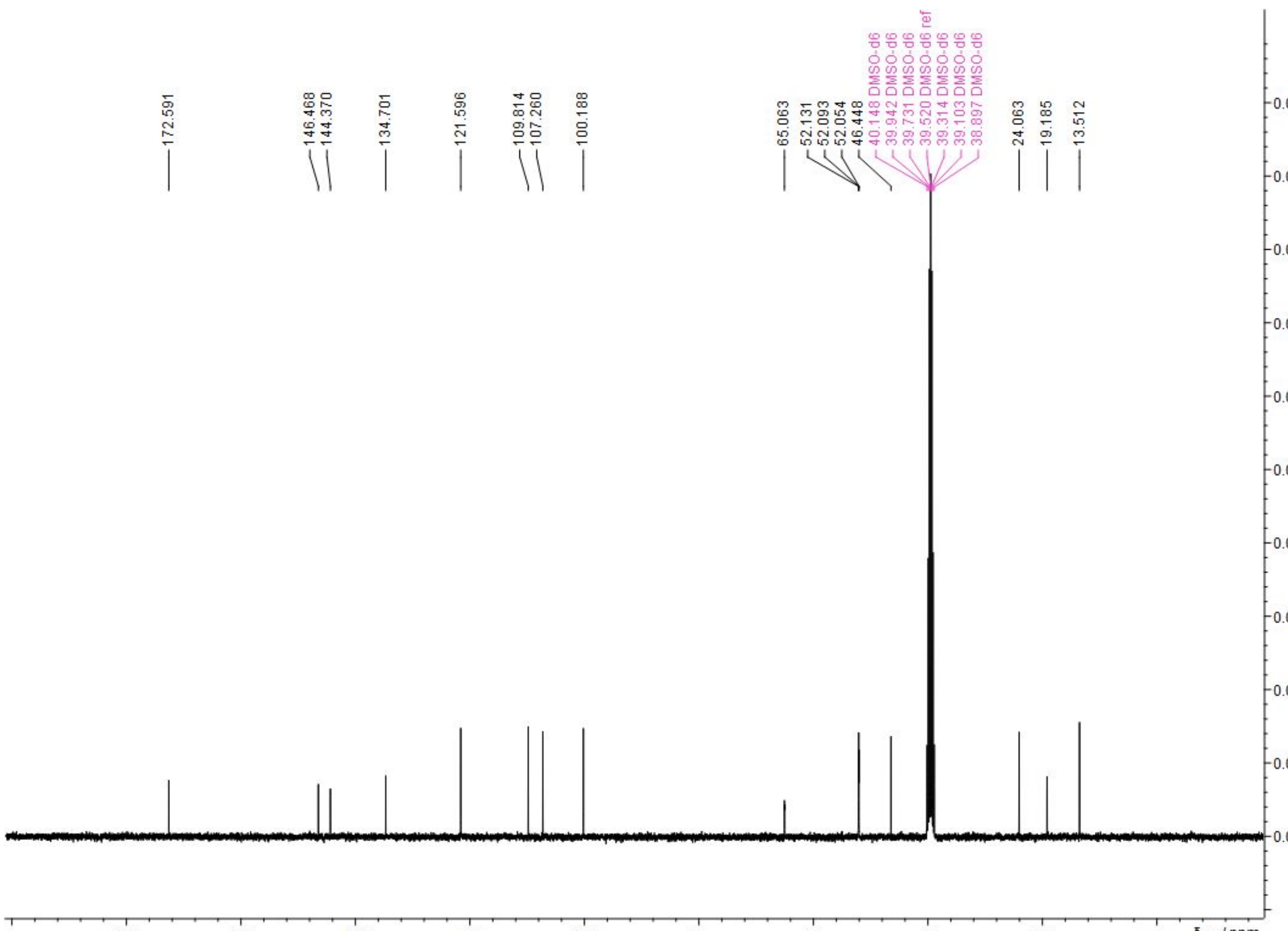

13 [TMC$_8$A][3,4-MPOAc]

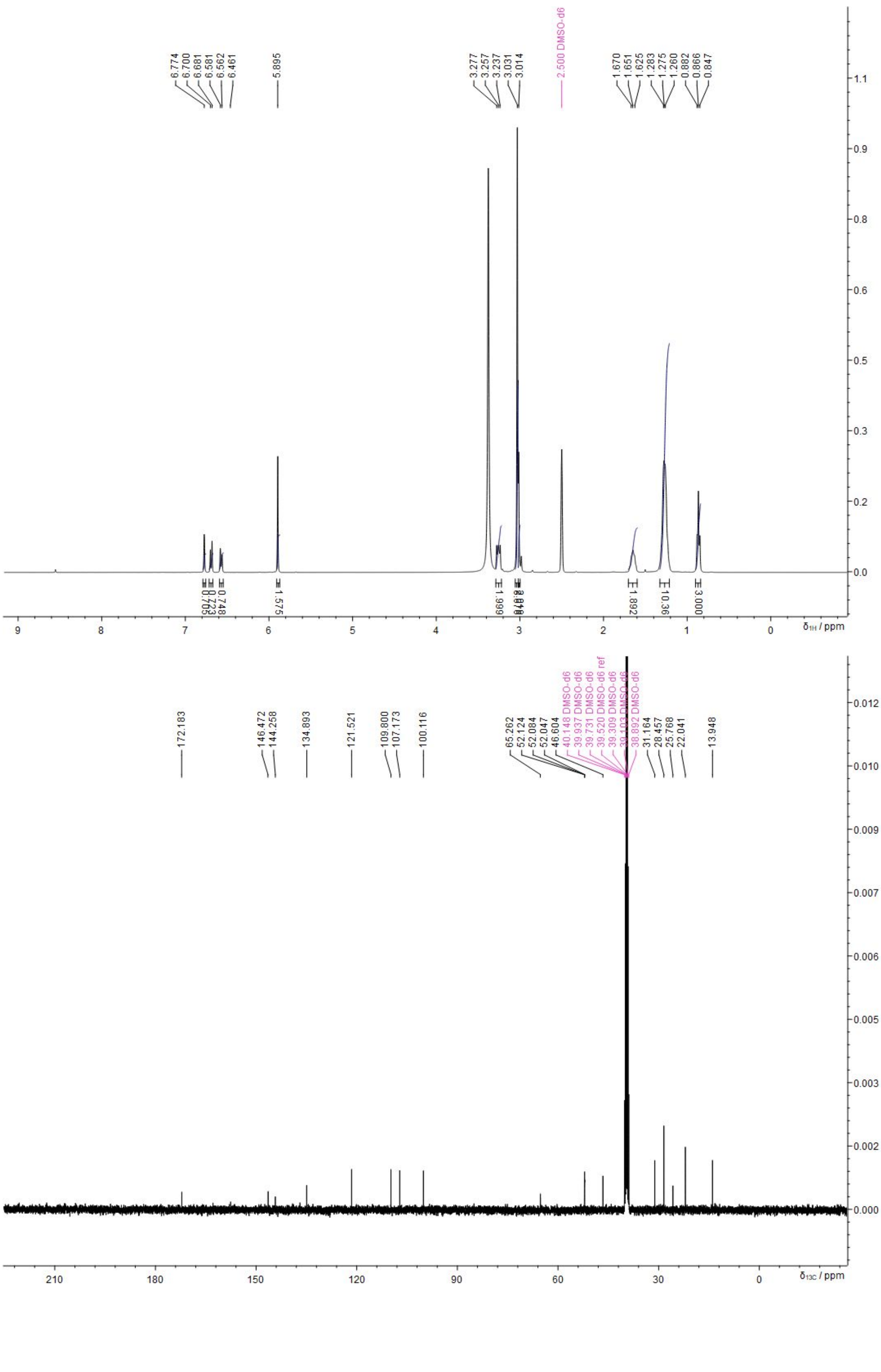

14 [$TMC_{16}A$][3,4-MPOAc]

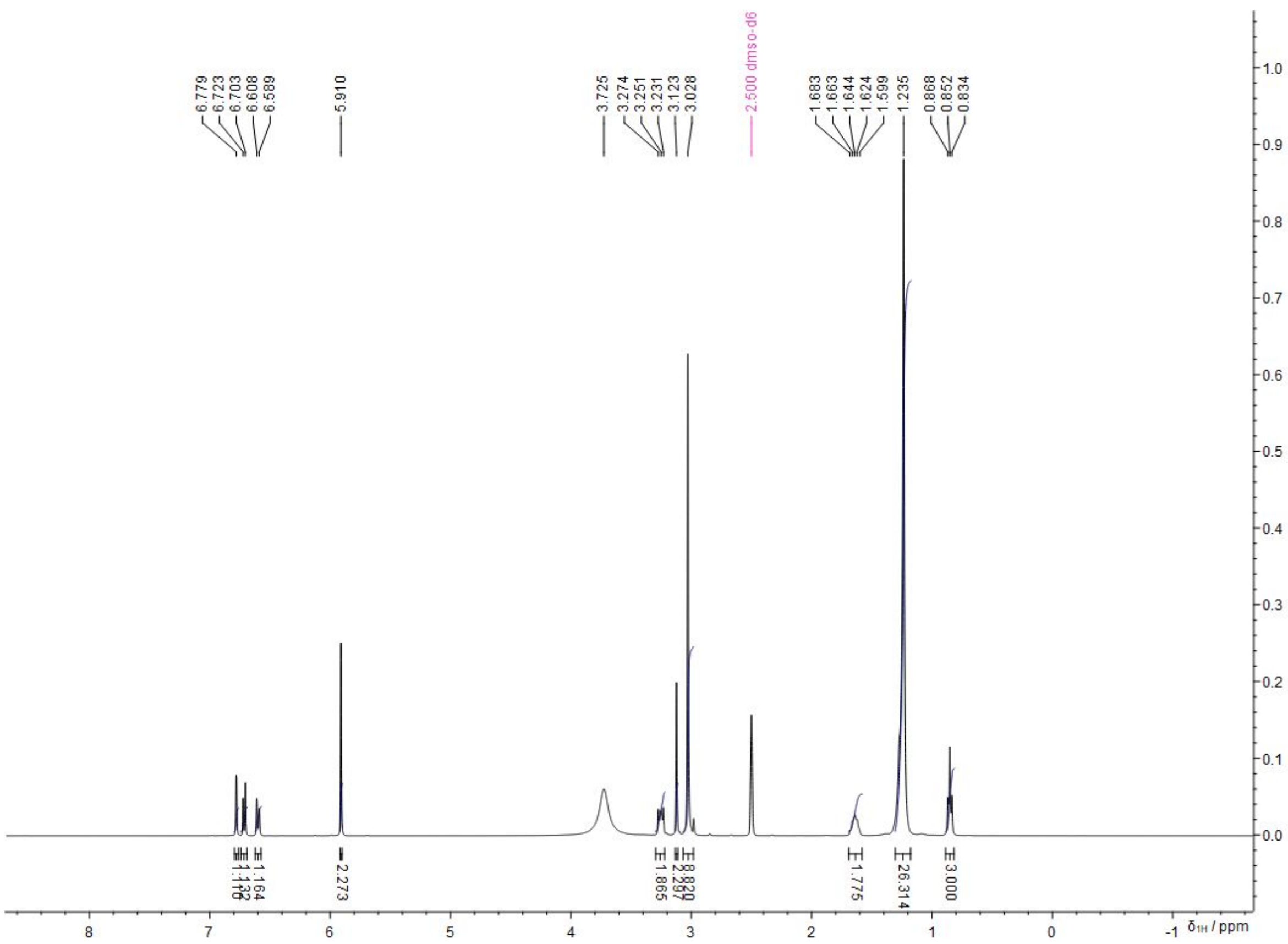

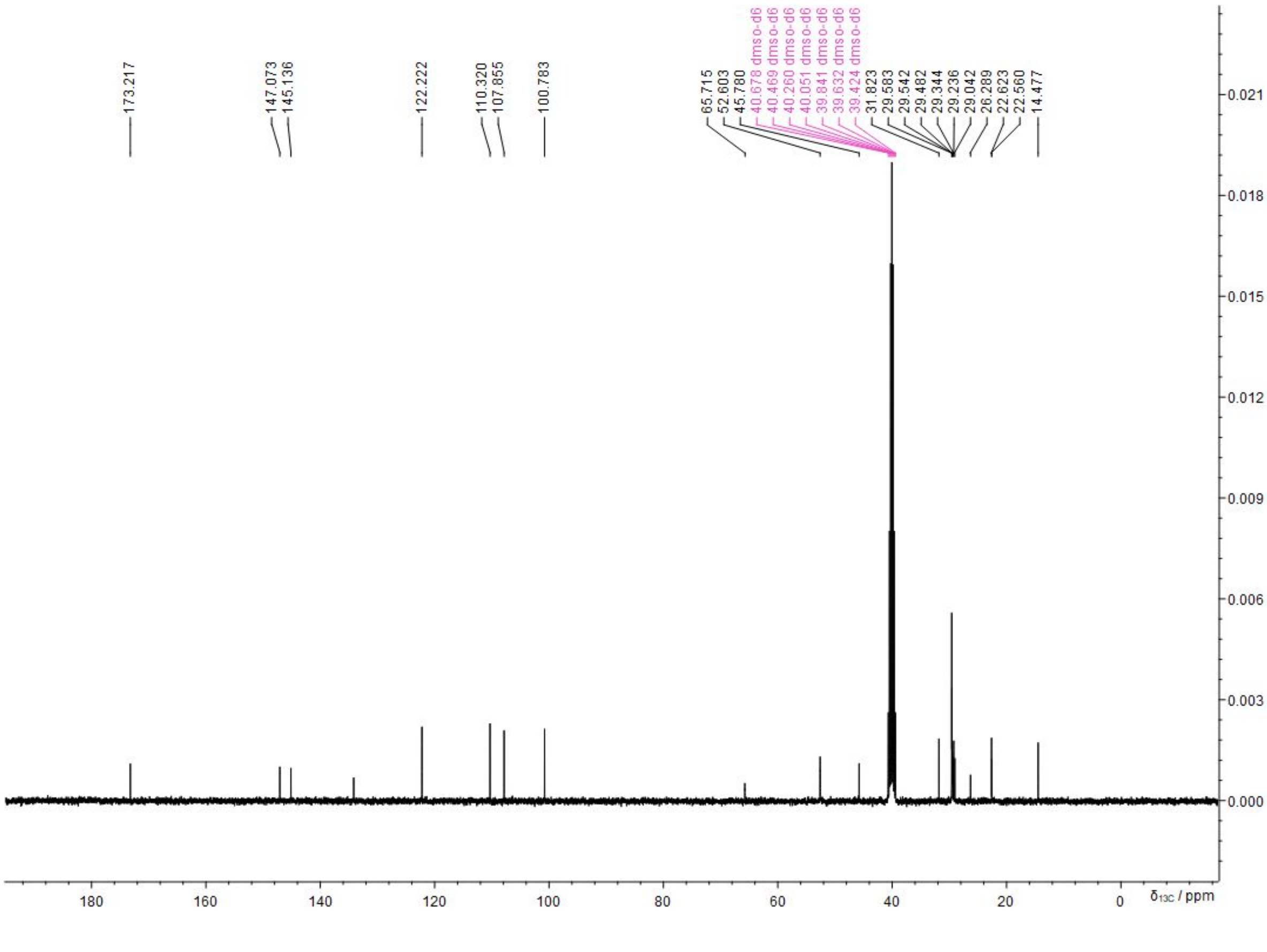

15 [TC$_4$MA][3,4-MPOAc]

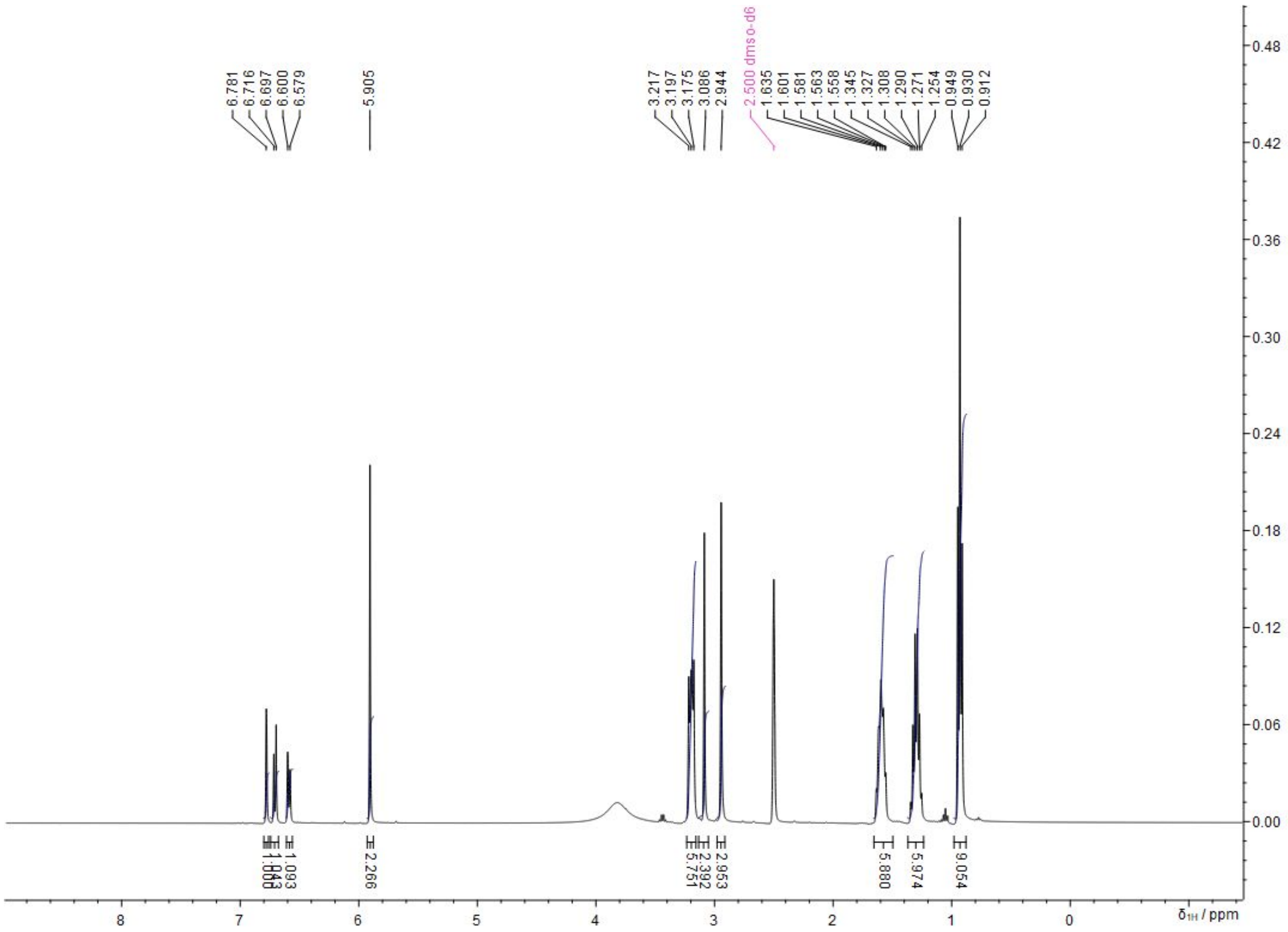

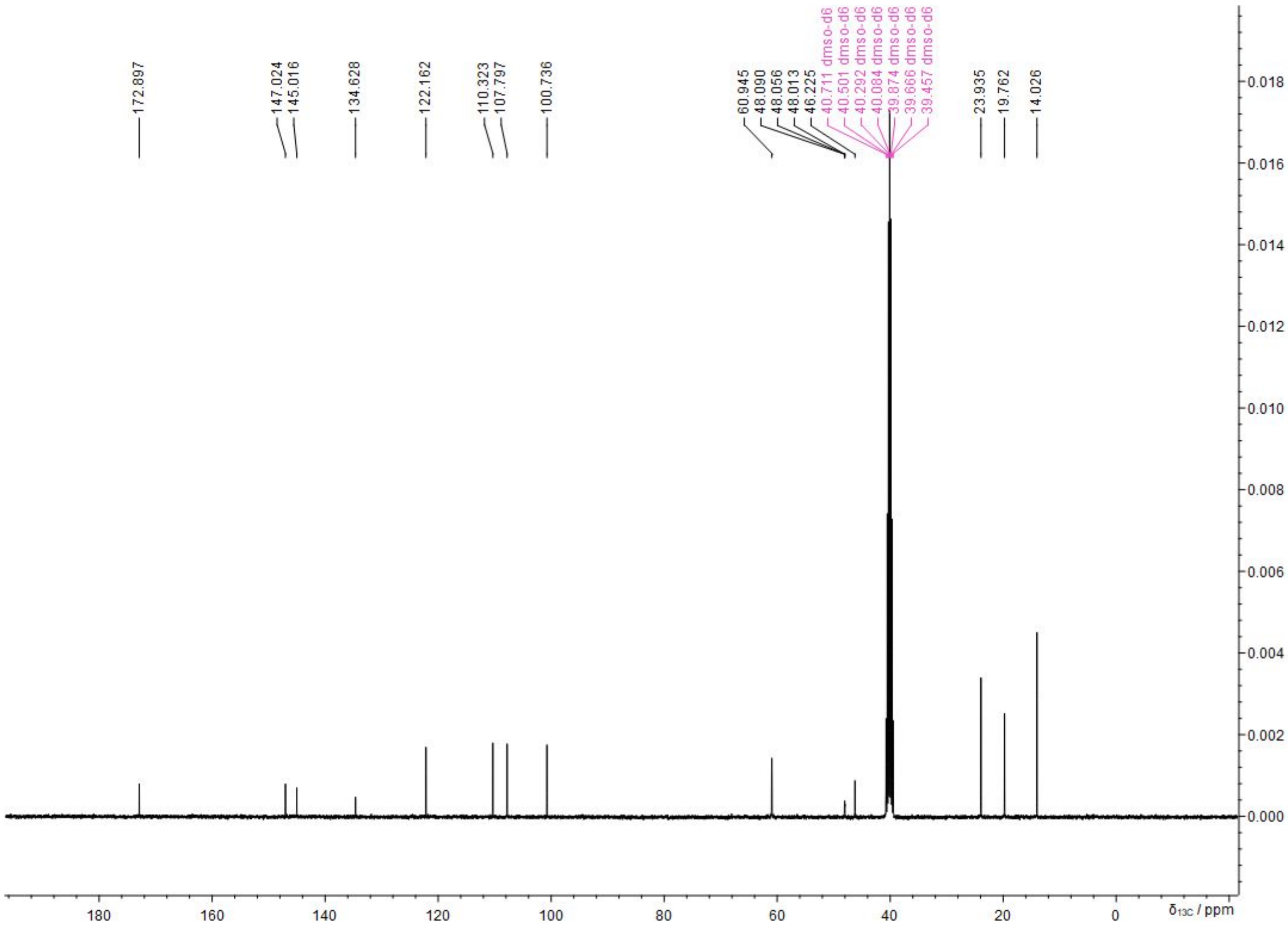

16 [TC_8MA][3,4-MPOAc]

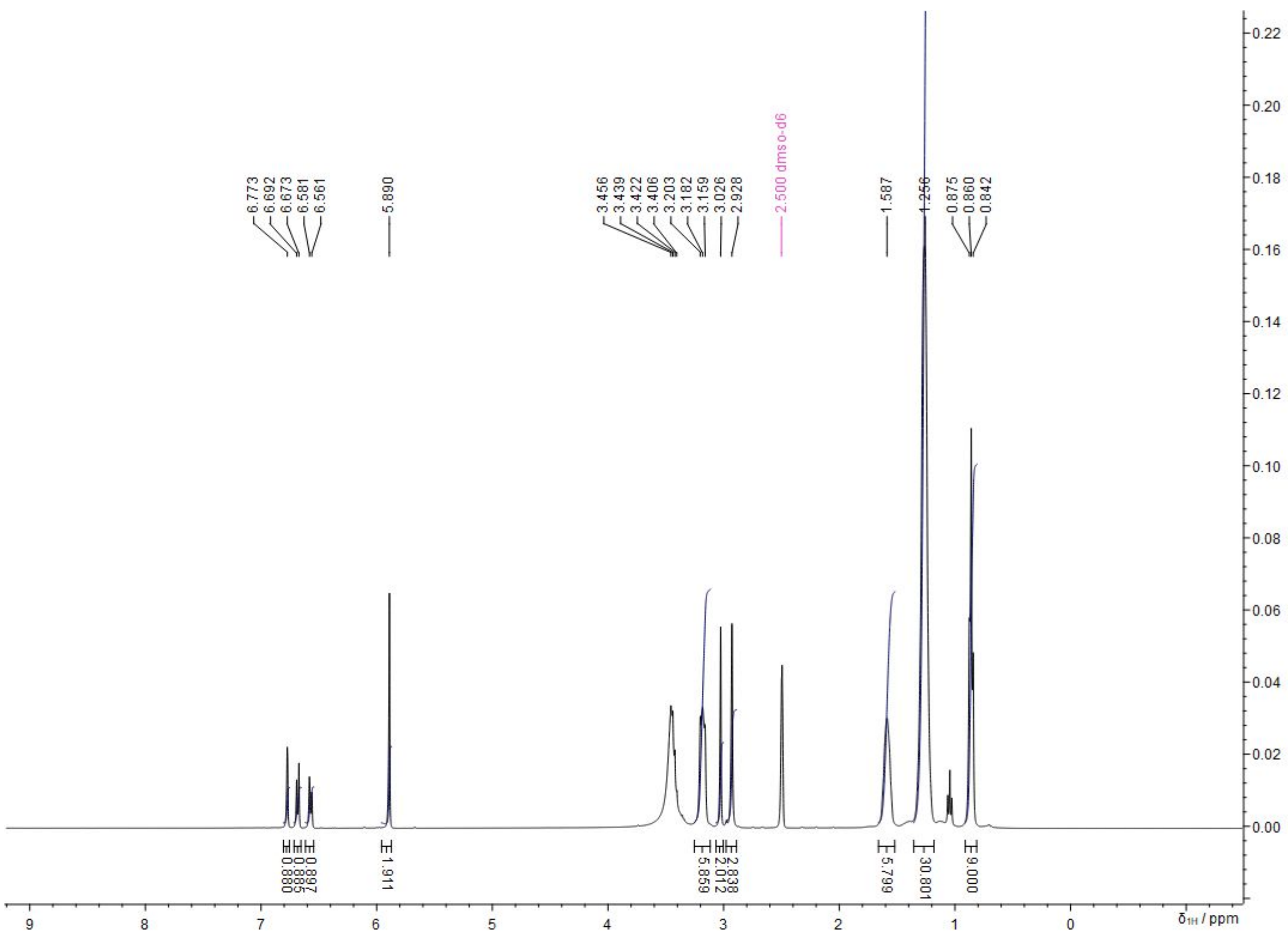

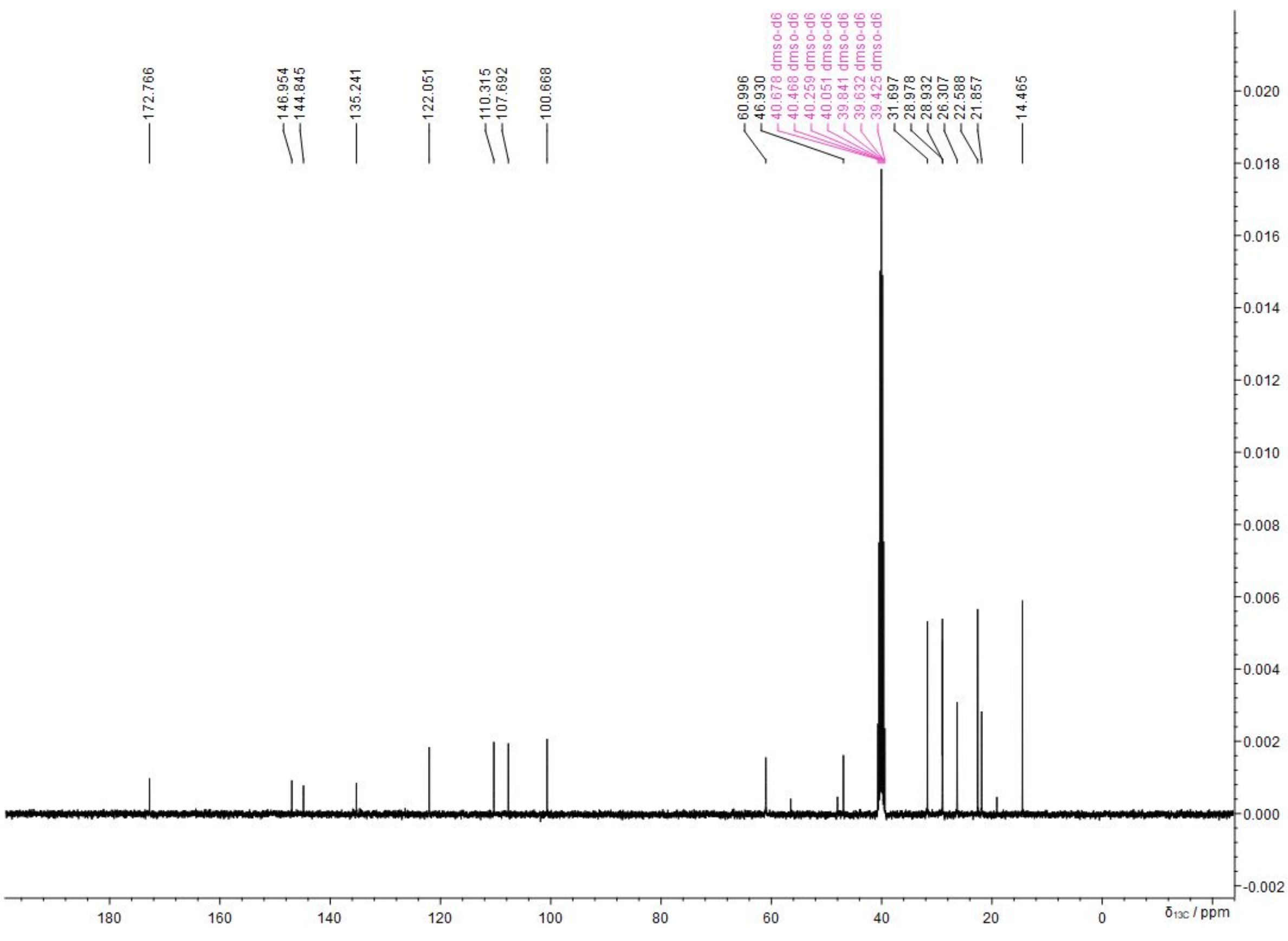

17 [C_2mim][3,4-MCOAc]

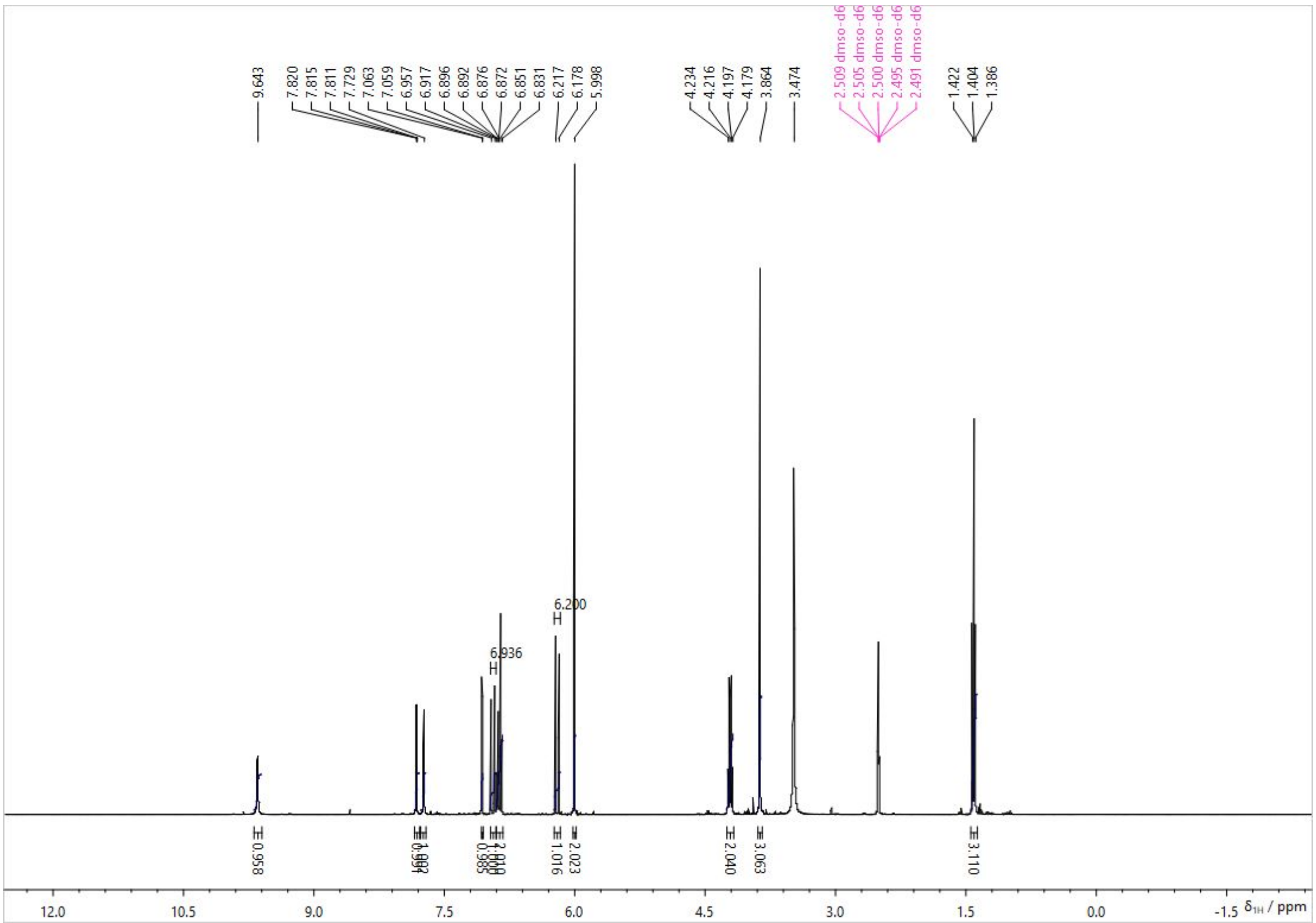

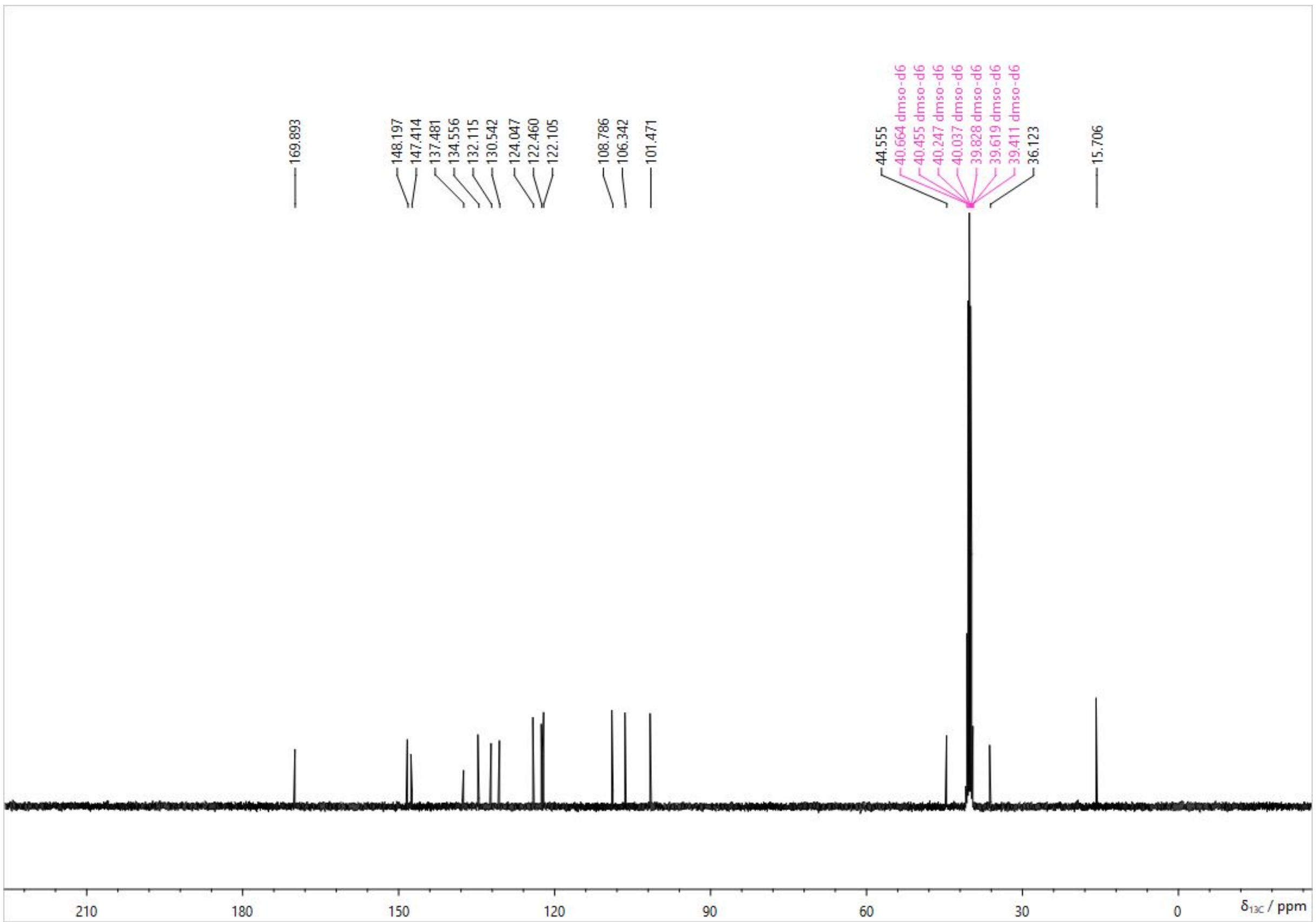

18 [C_4mim][3,4-MCOAc]

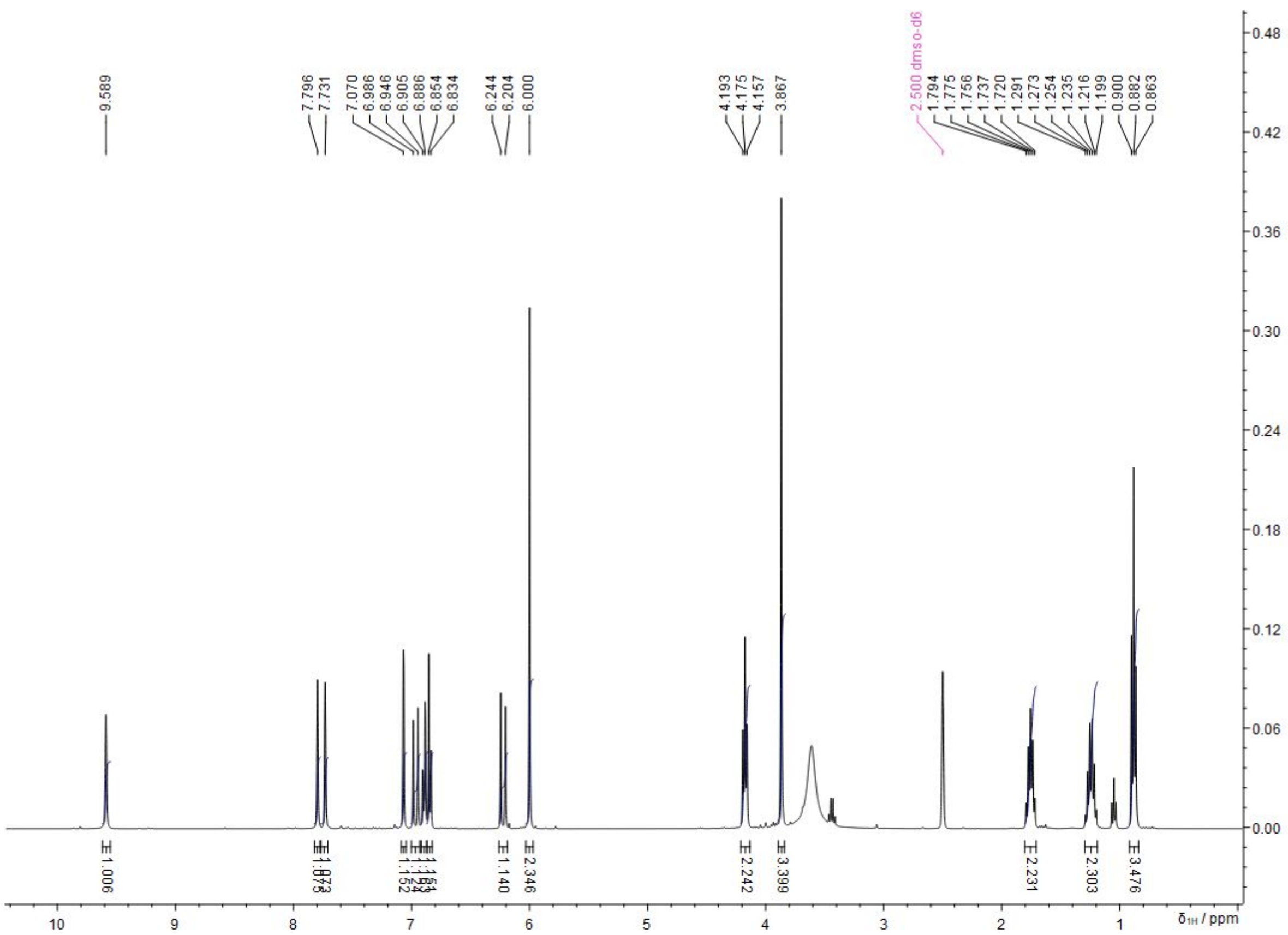

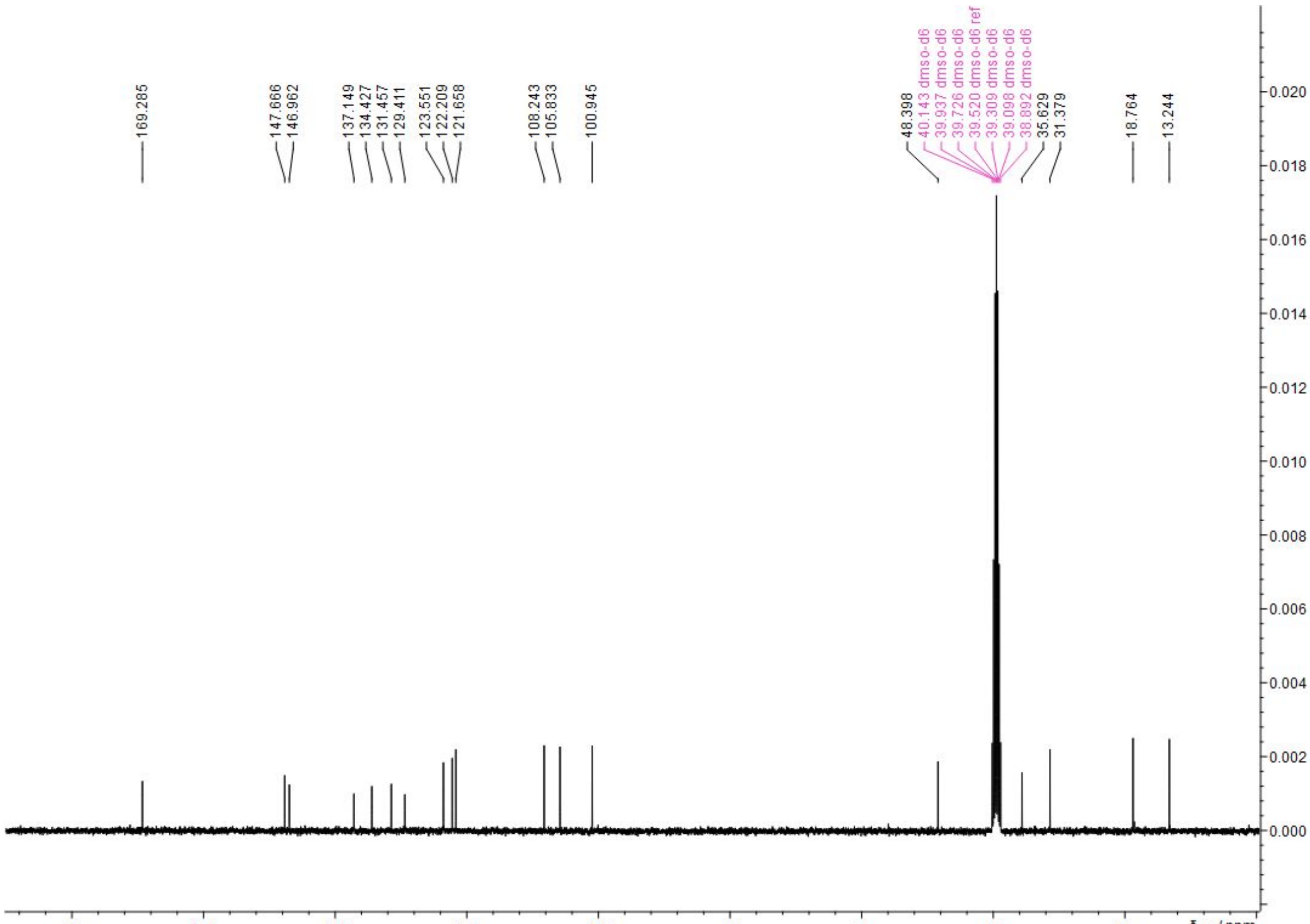

19 [TMiPrA][3,4-MCOAc]

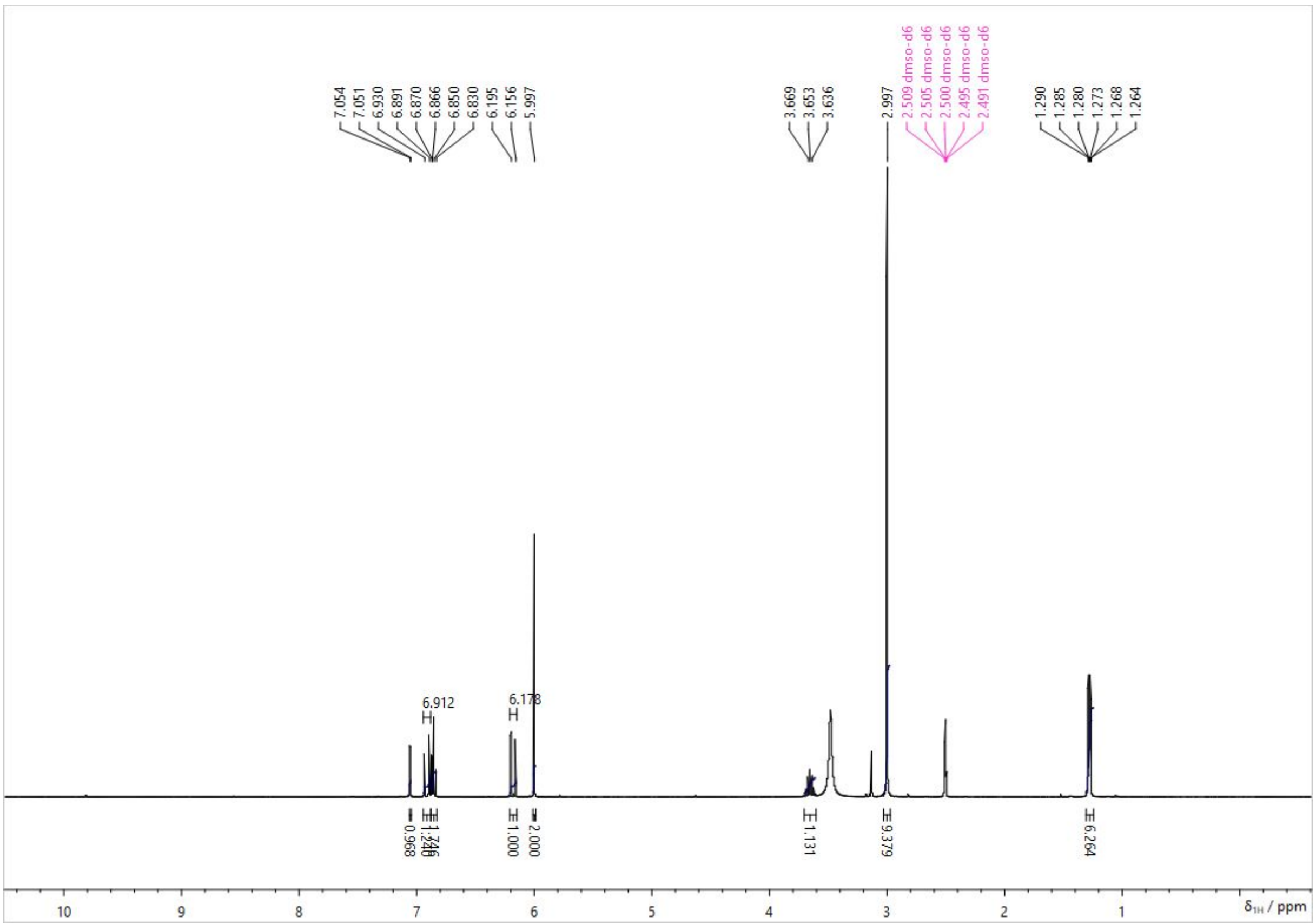

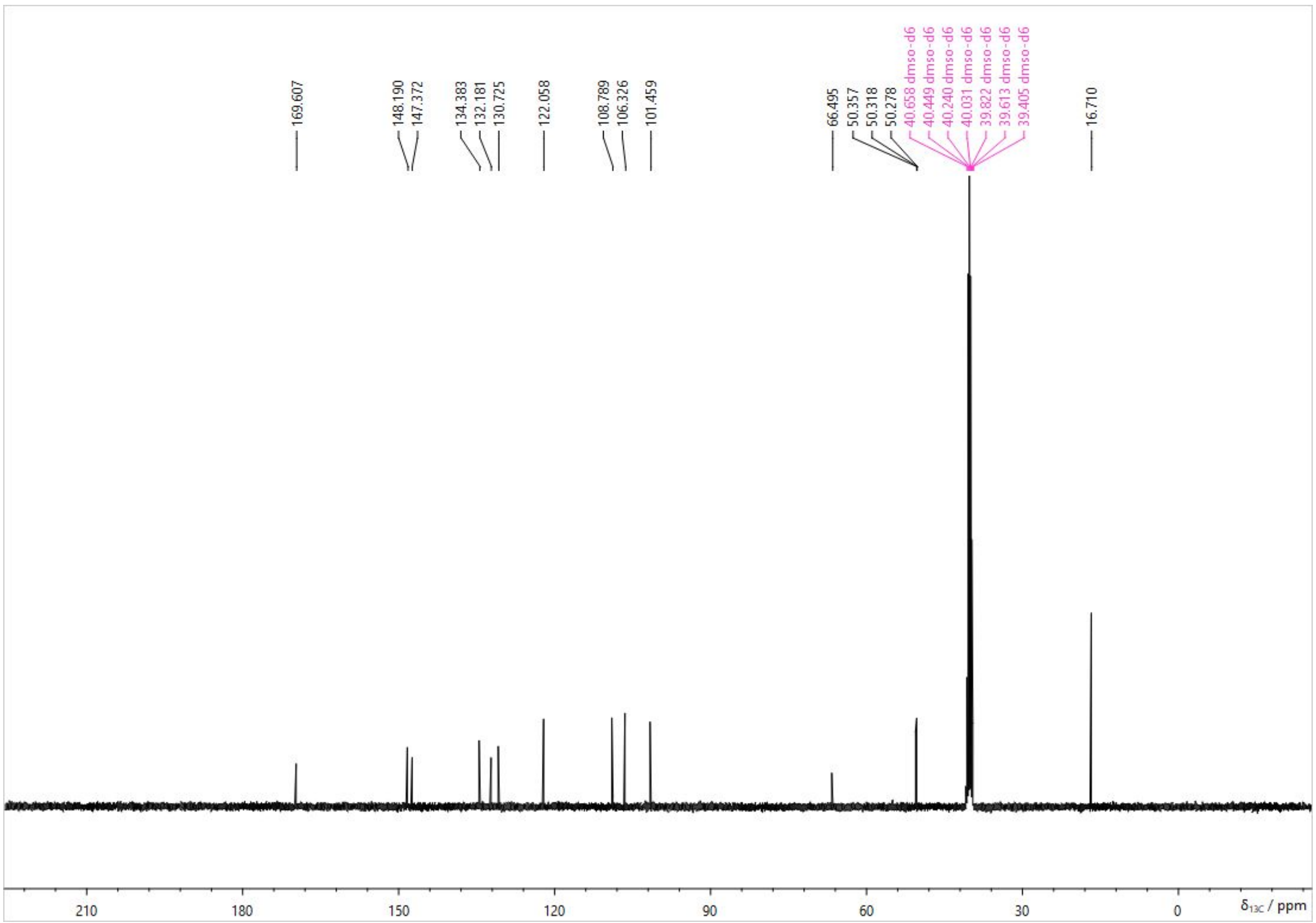

20 [TMC$_4$A][3,4-MCOAc]

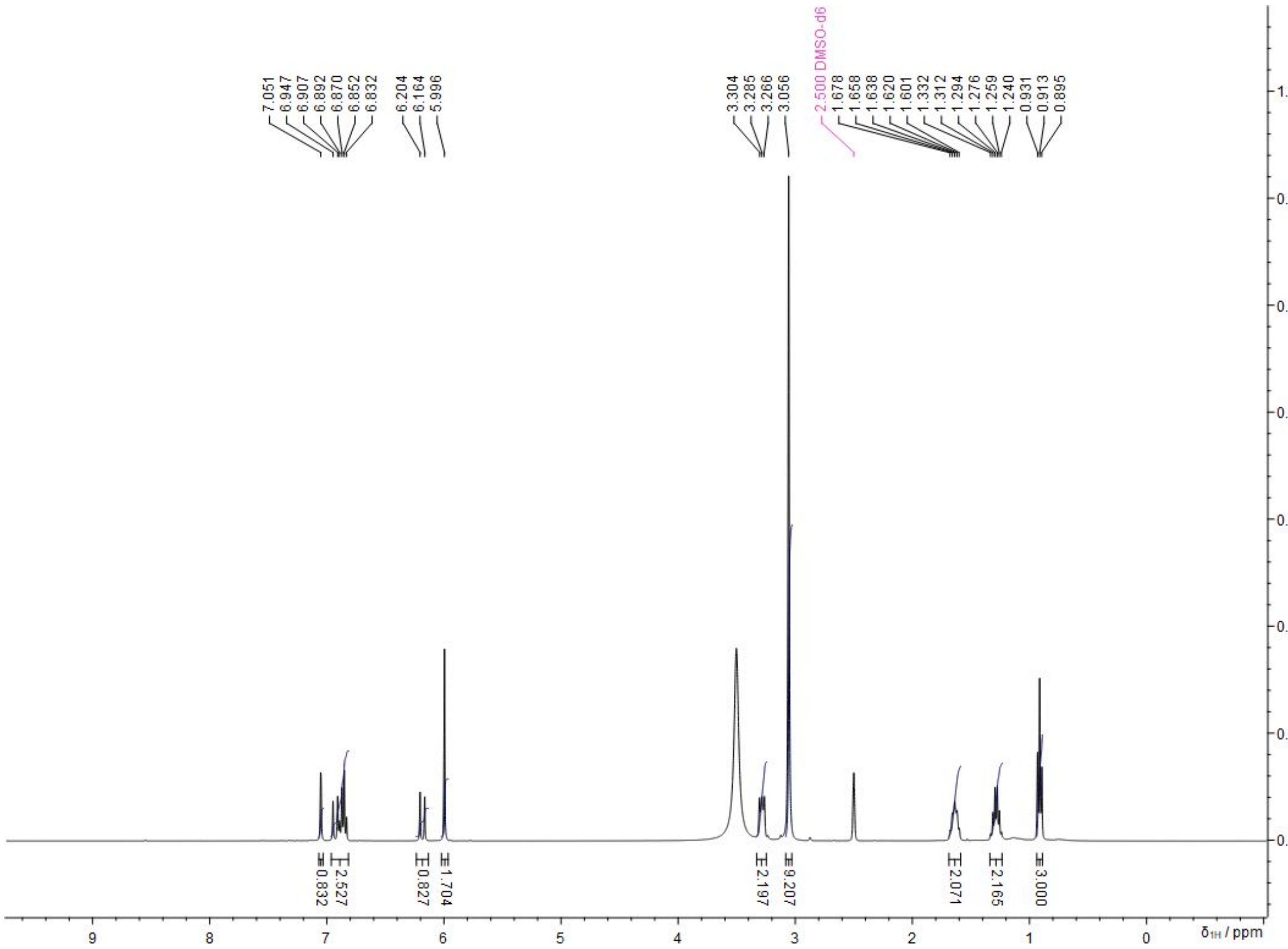

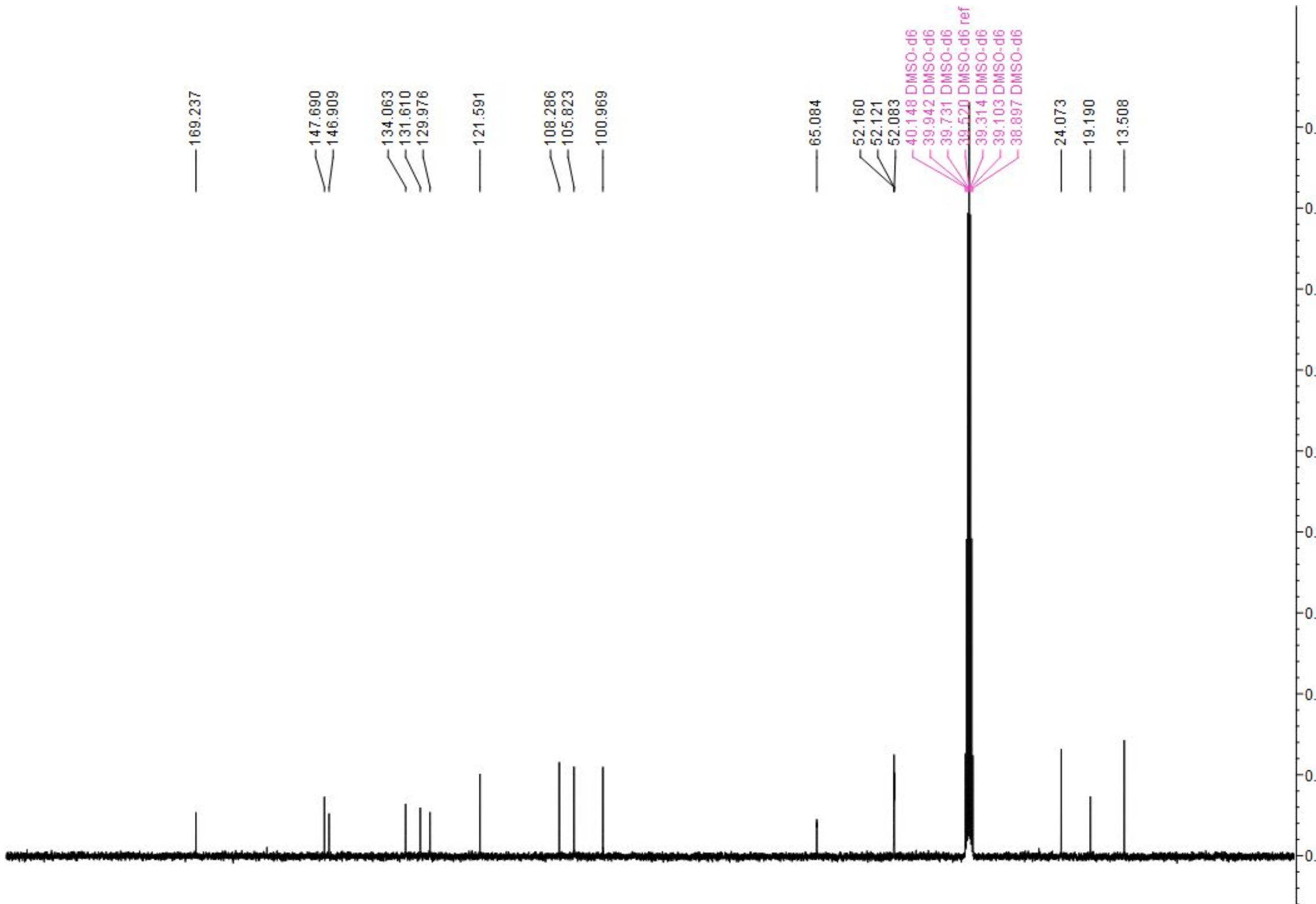

21 [TMC$_8$A][3,4-DCOAc]

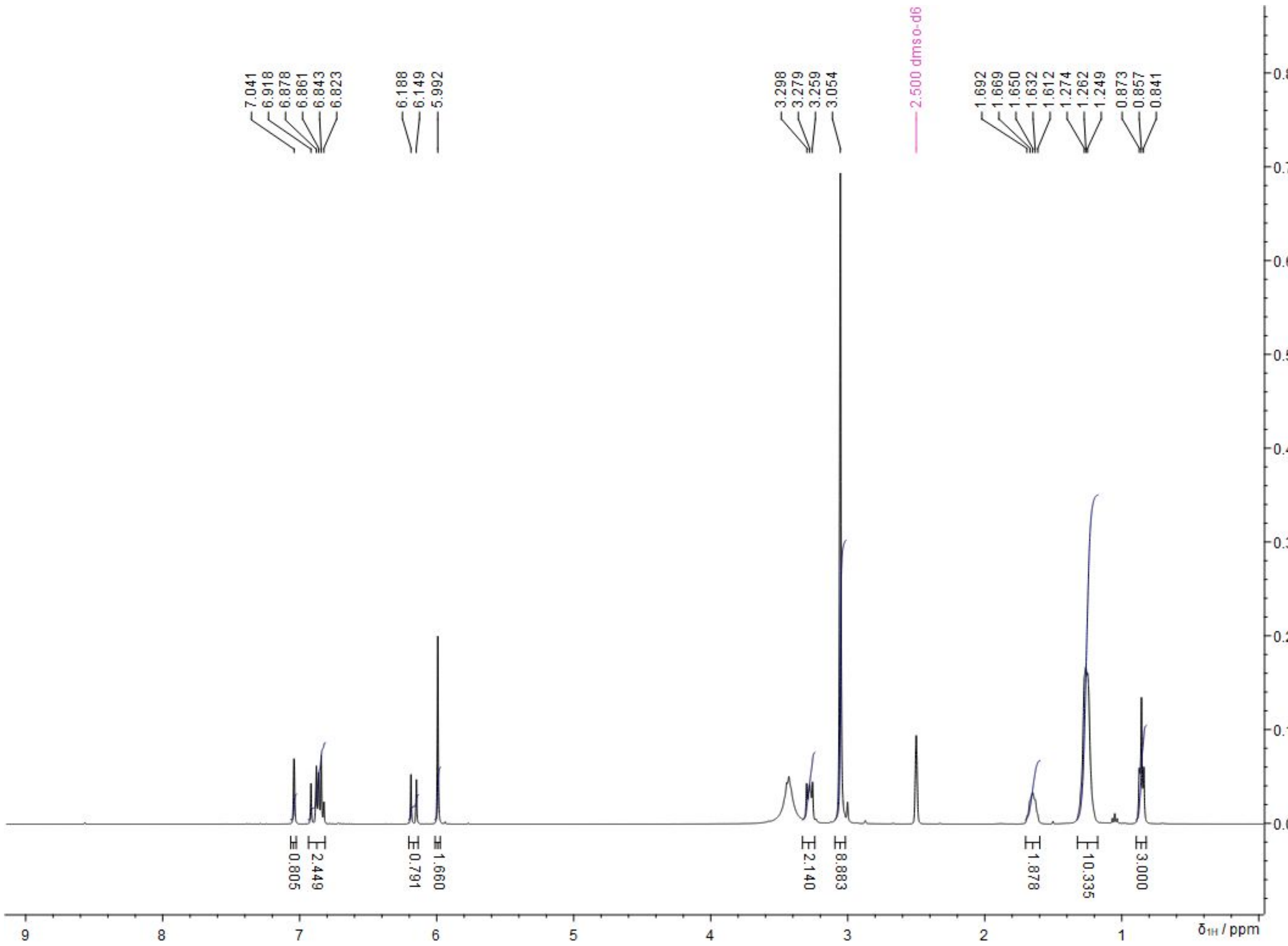

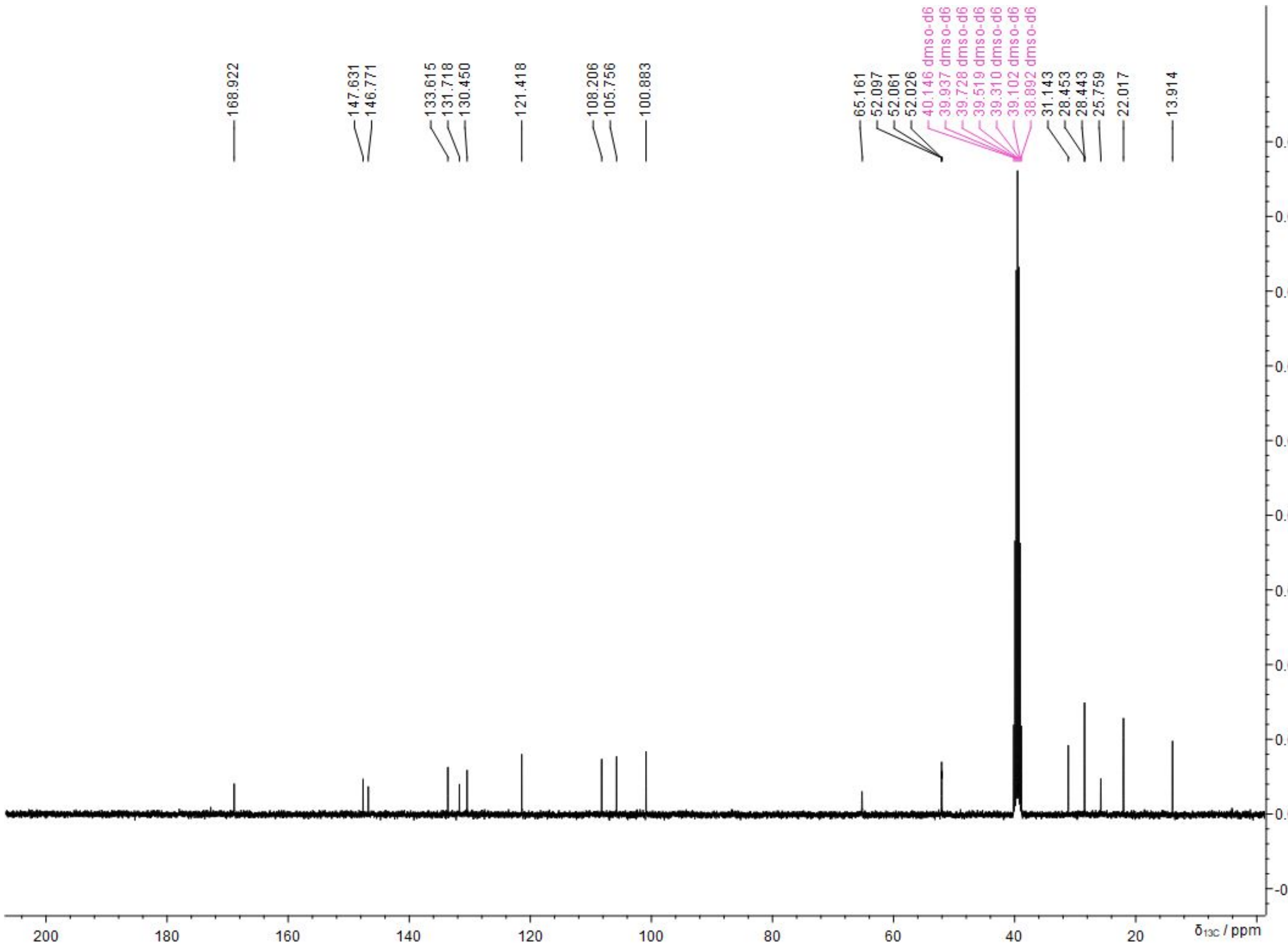

22 [$TMC_{16}A$][3,4-MCOAc]

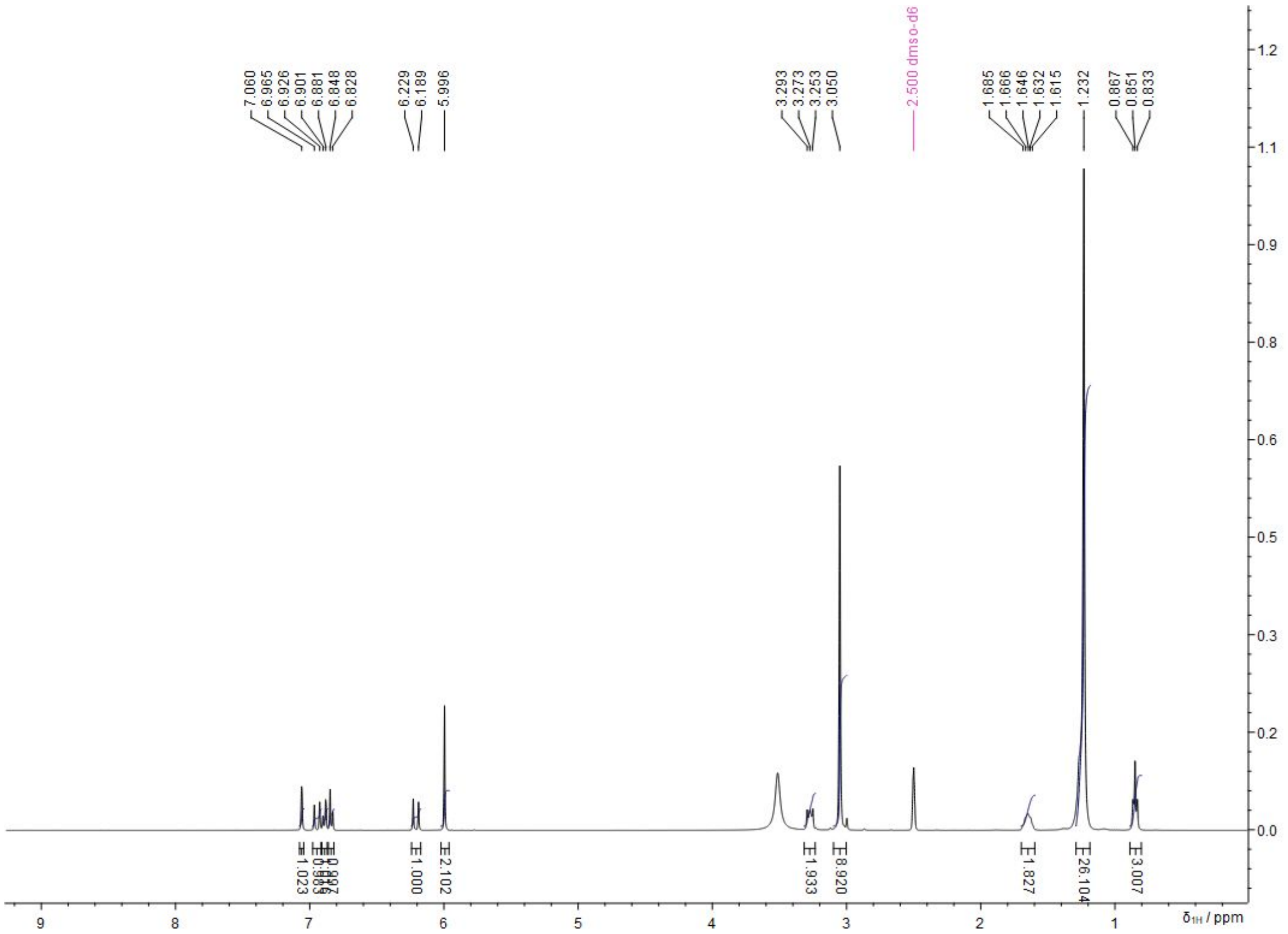

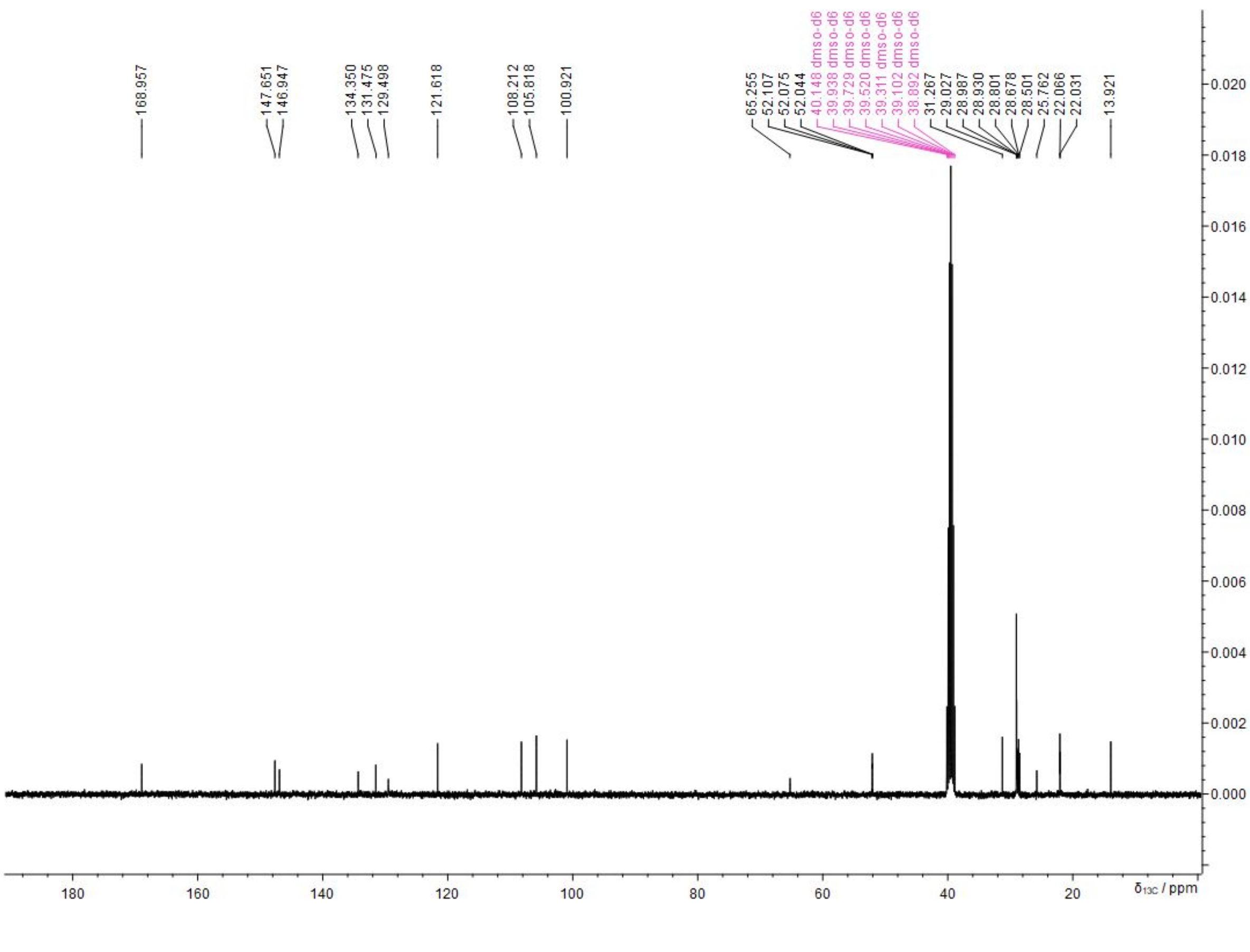

23 [TC_4MA][3,4-MCOAc]

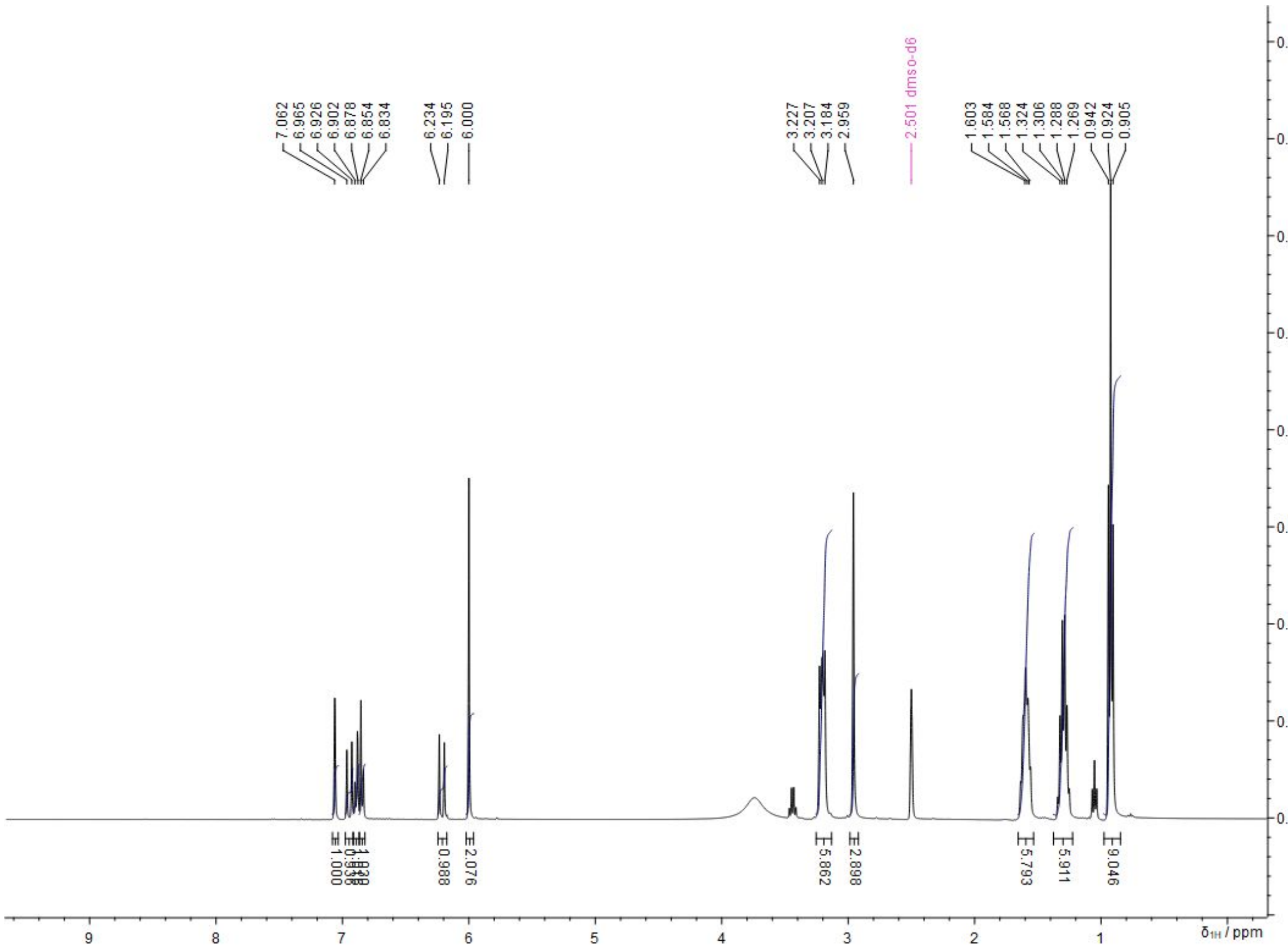

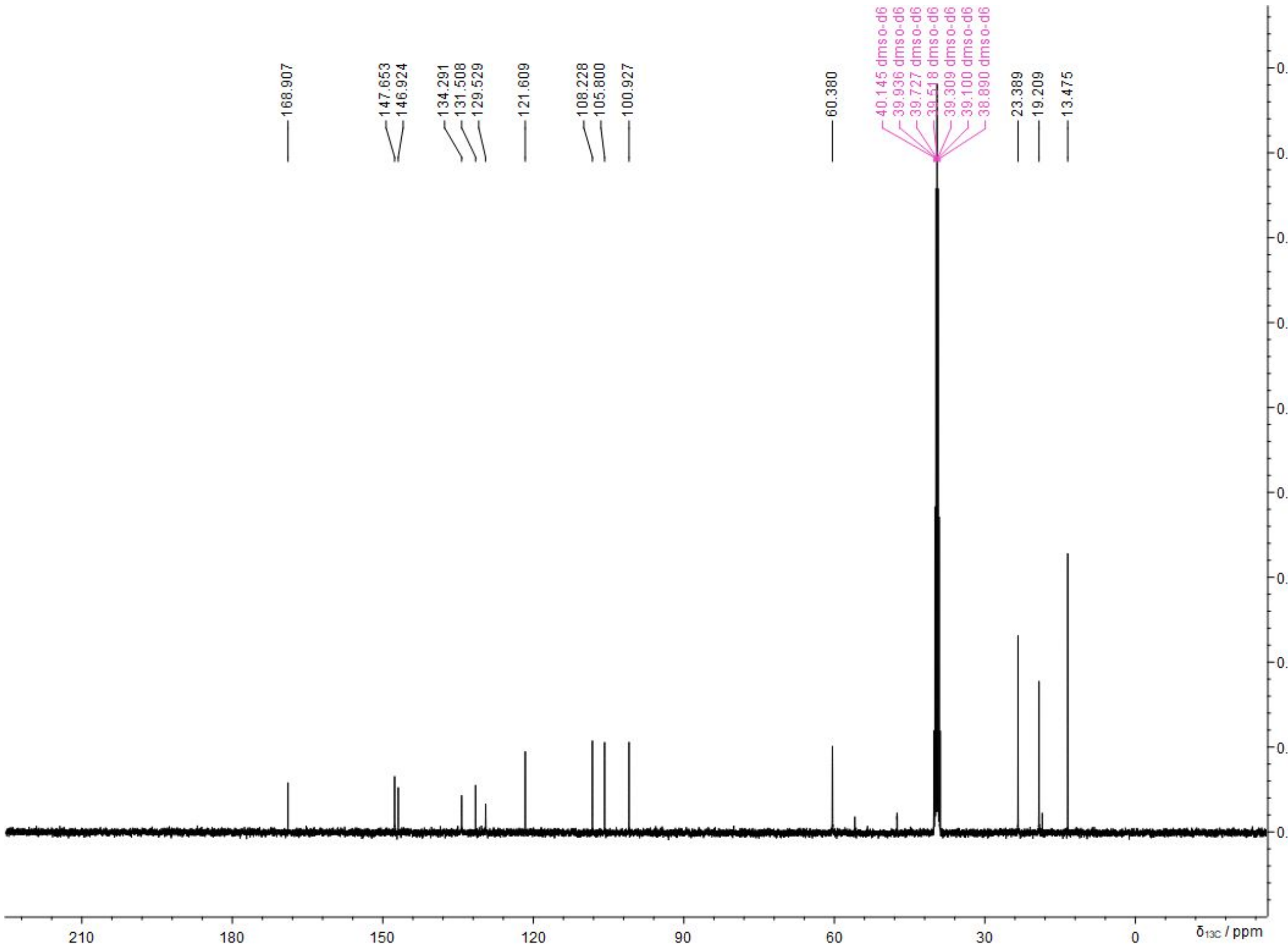

24 [TC_8MA][3,4-MCOAc]

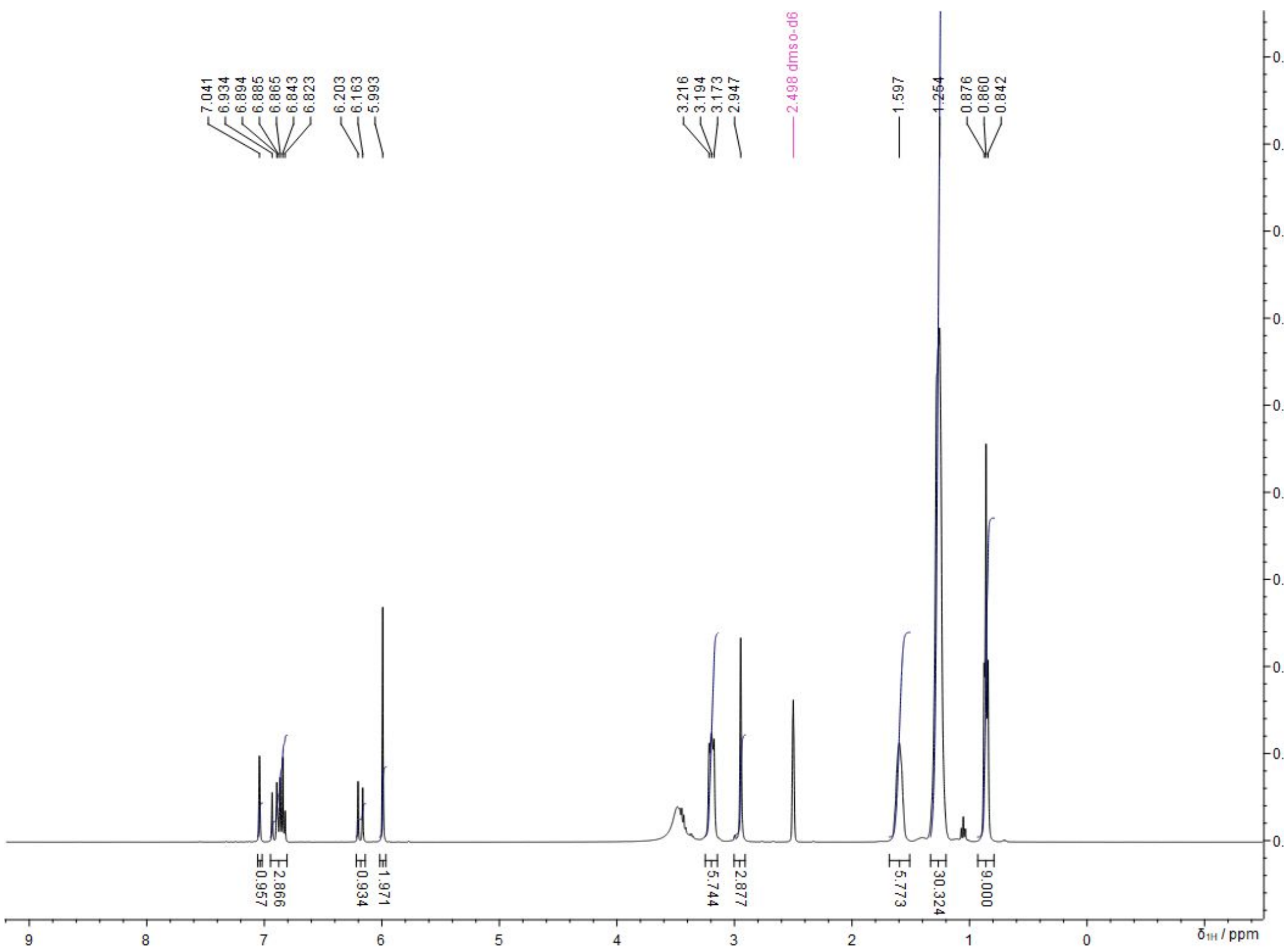

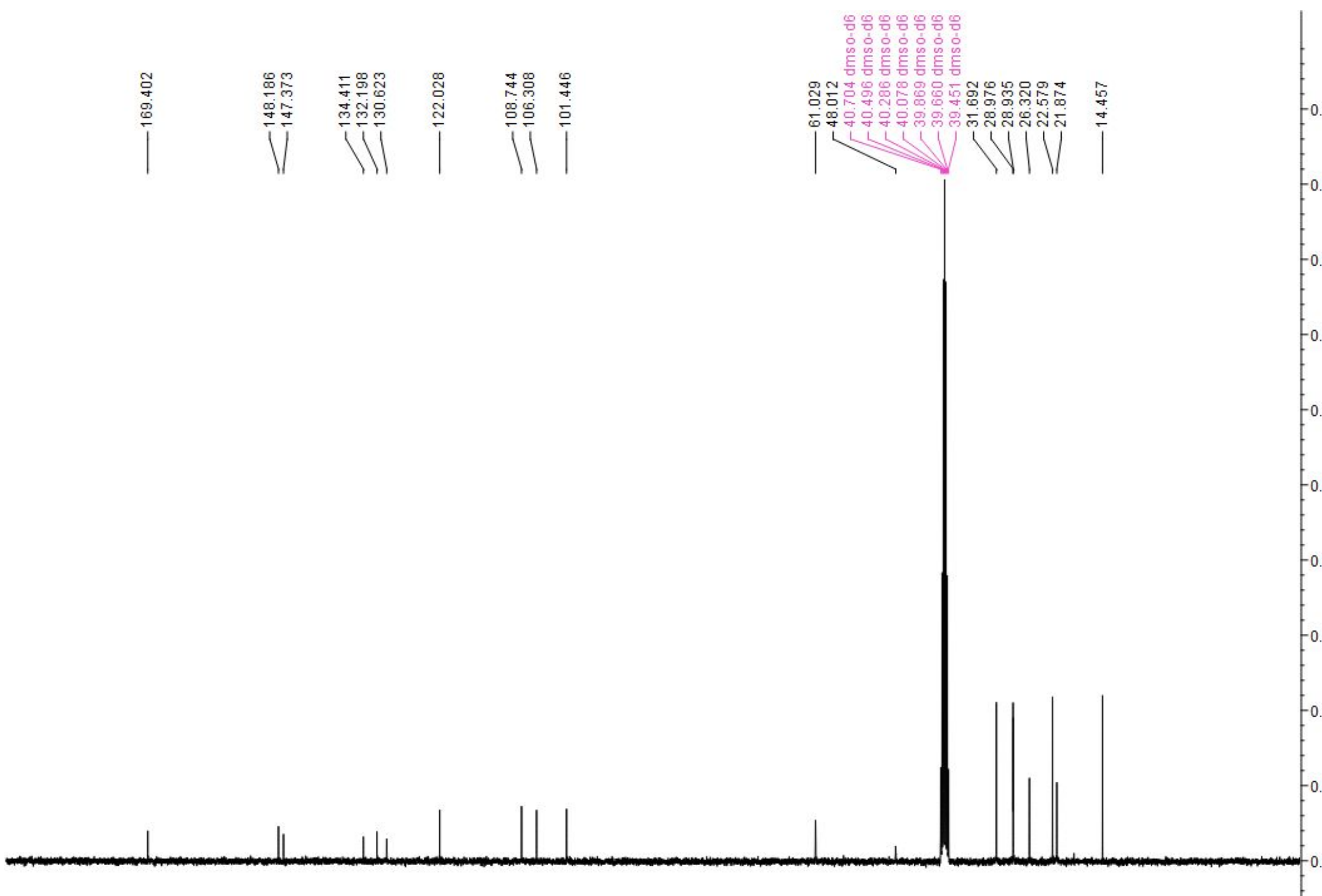

25 [C_2mim][3,4-DCOAc]

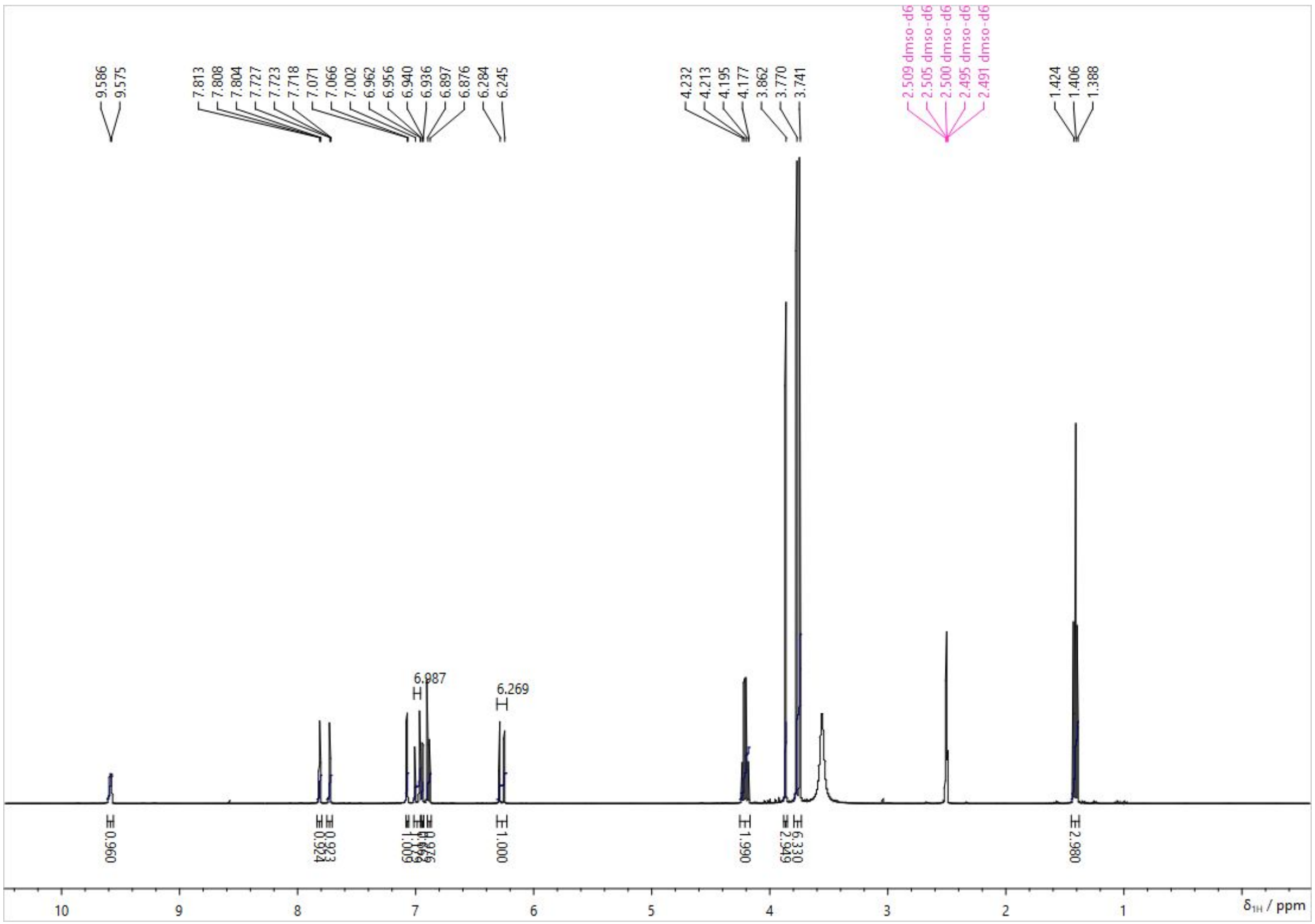

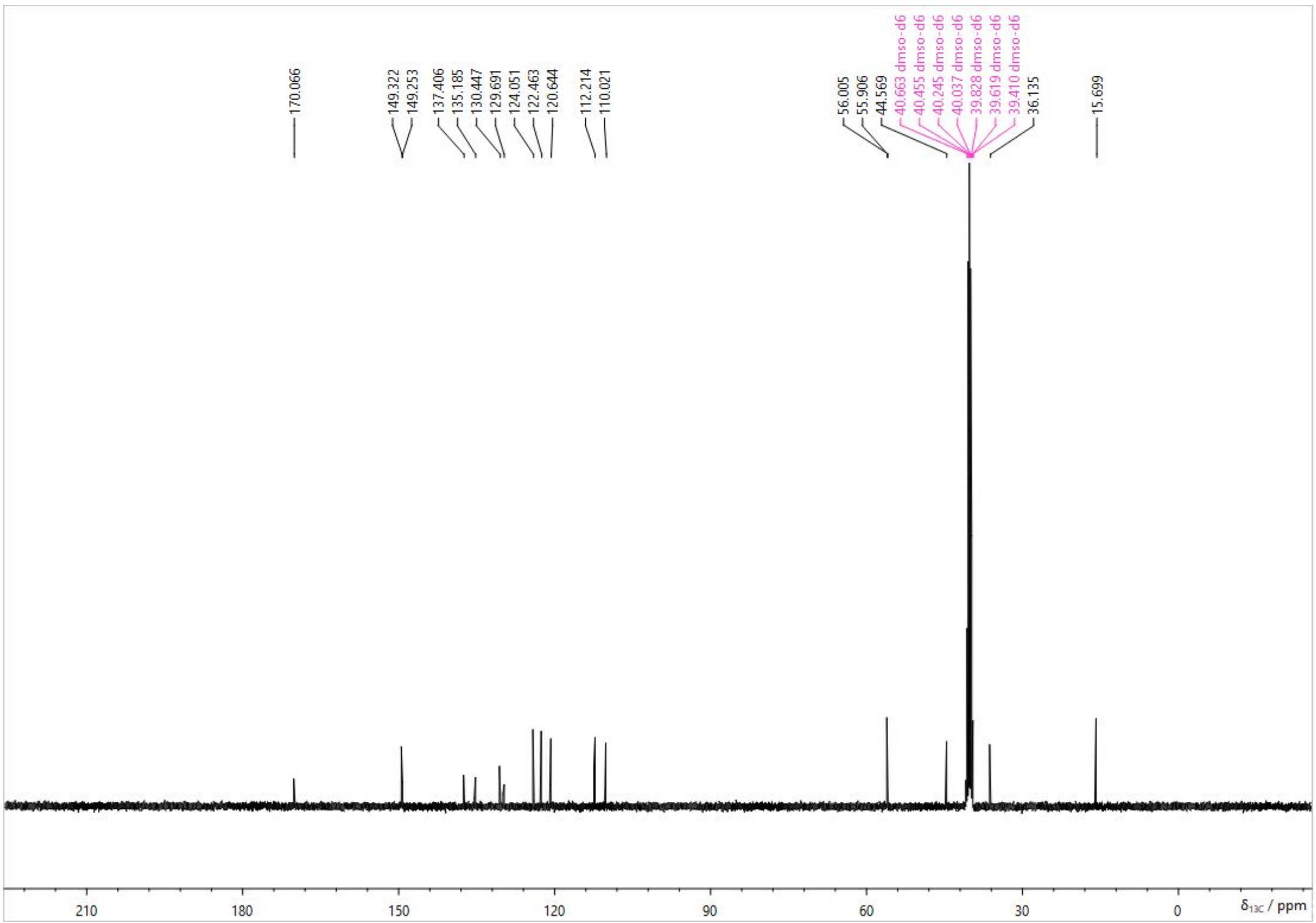

26 [C$_4$mim][3,4-DCOAc]

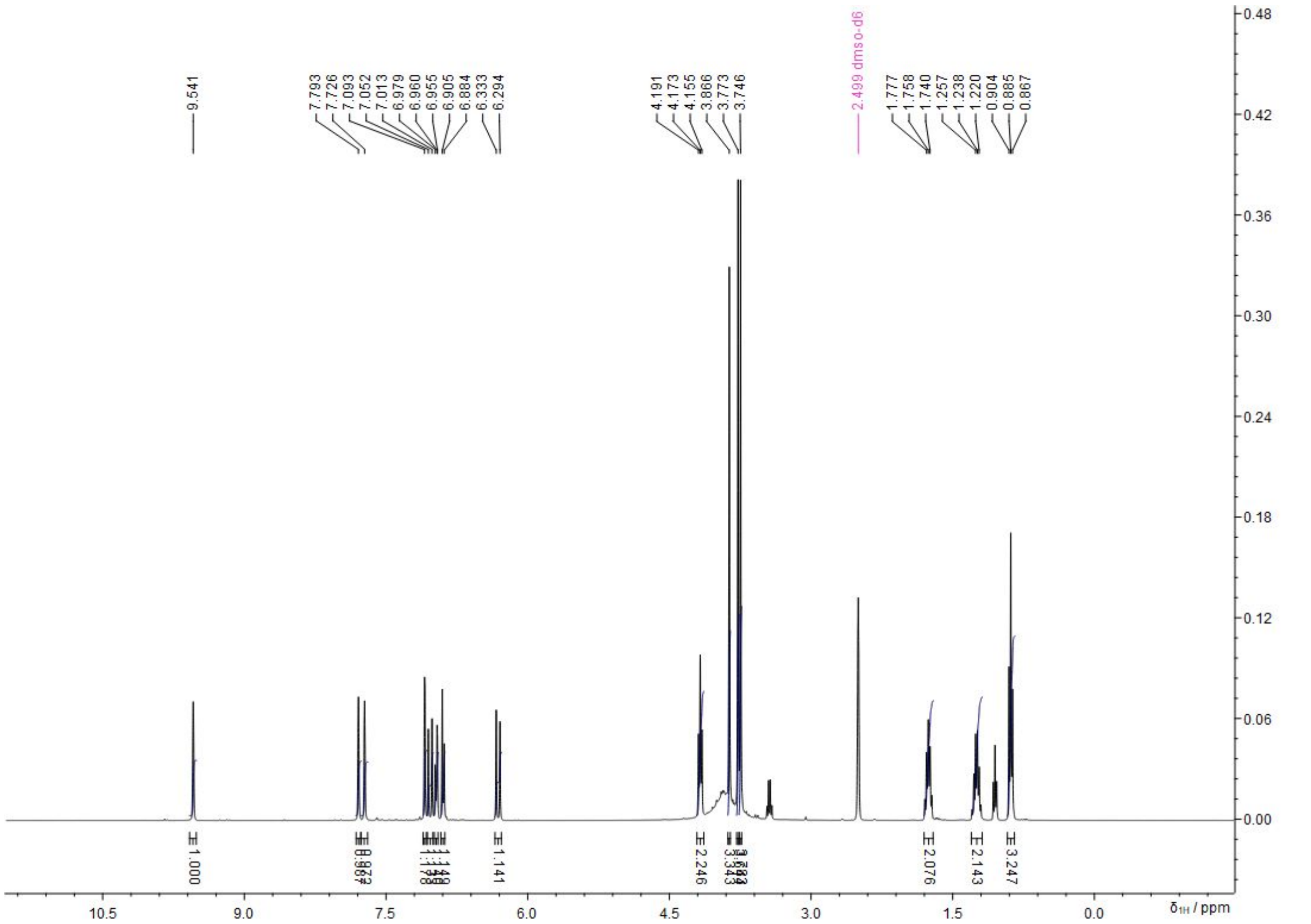

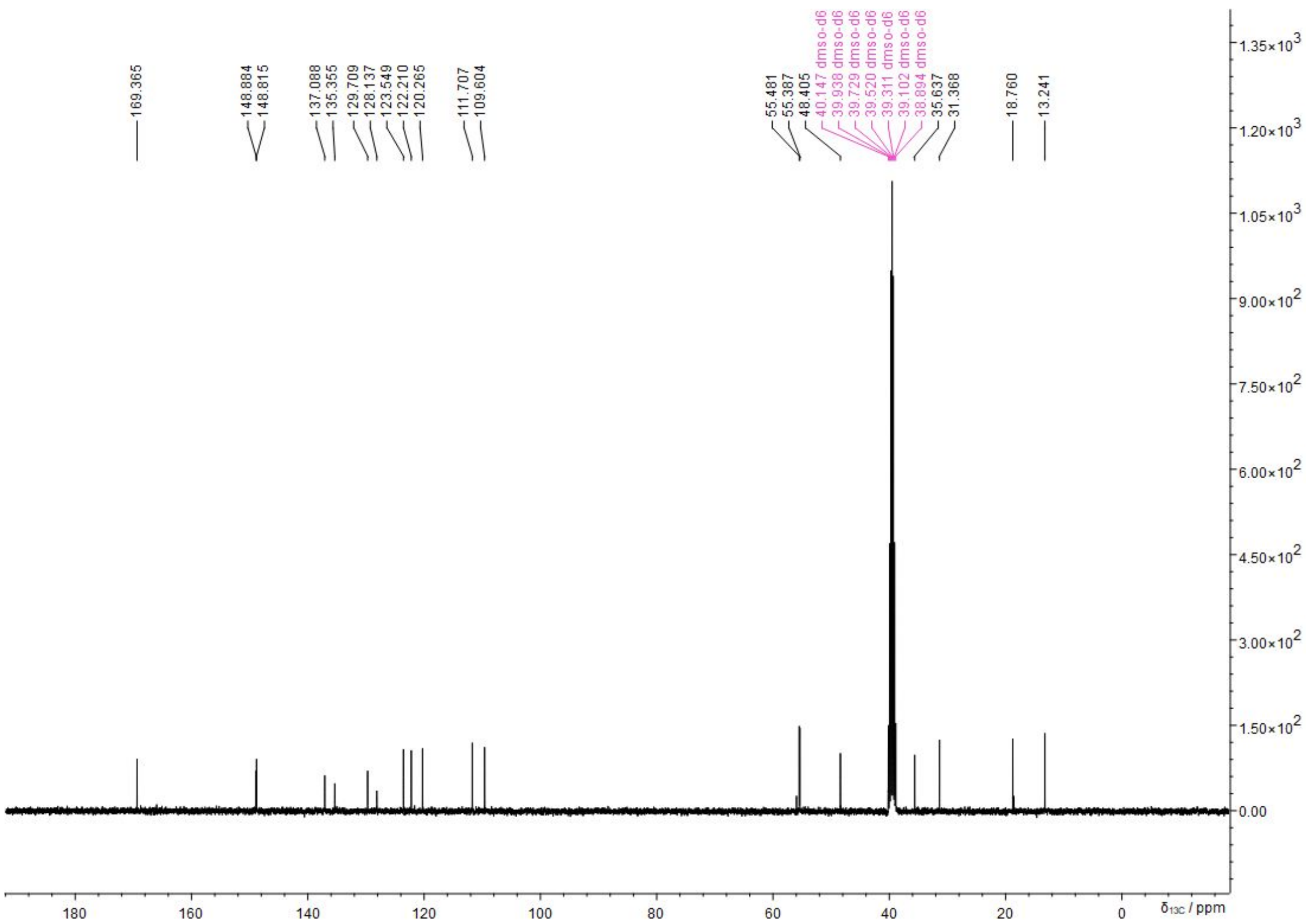

27 [TMiPrA][3,4-DCOAc]

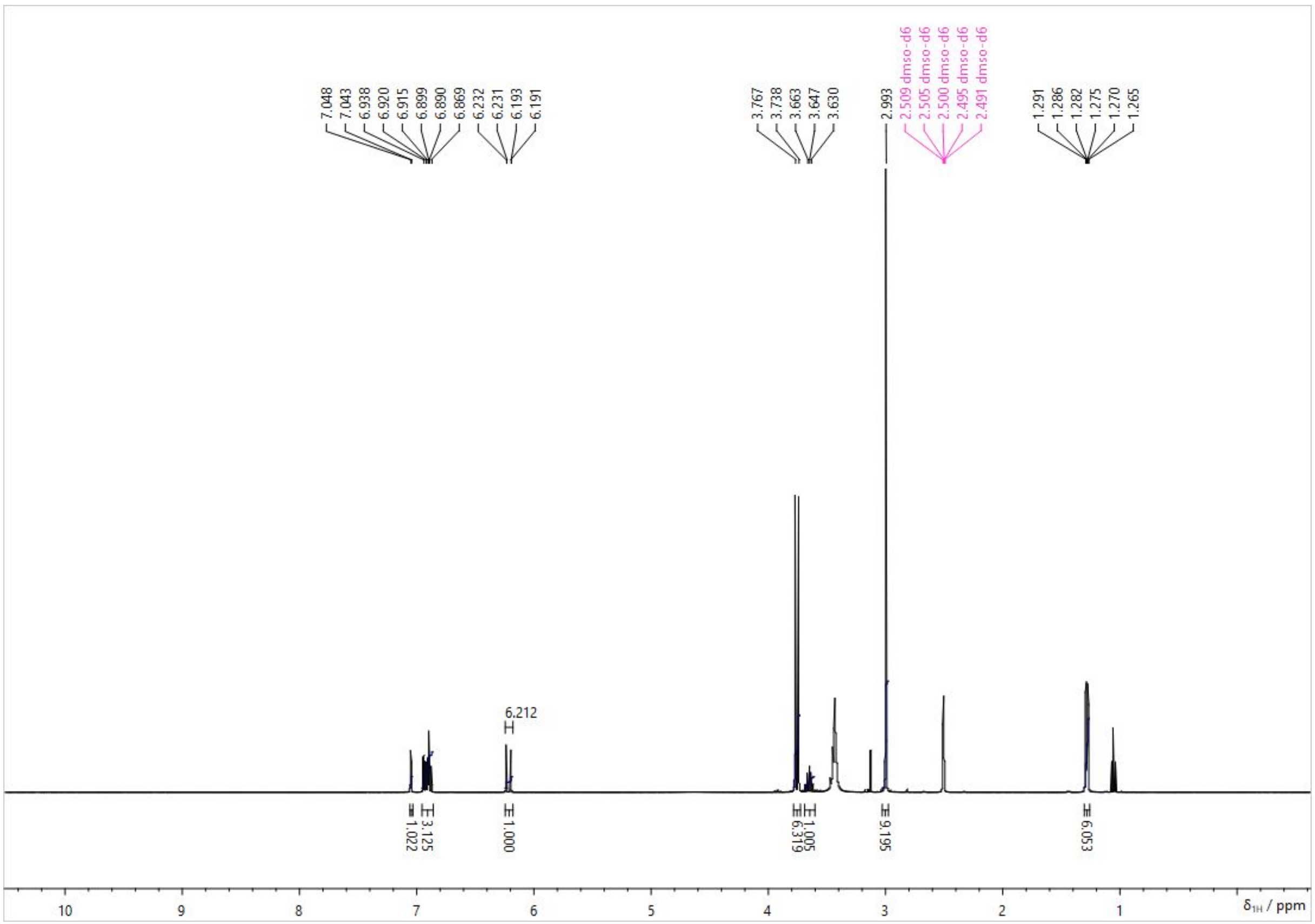

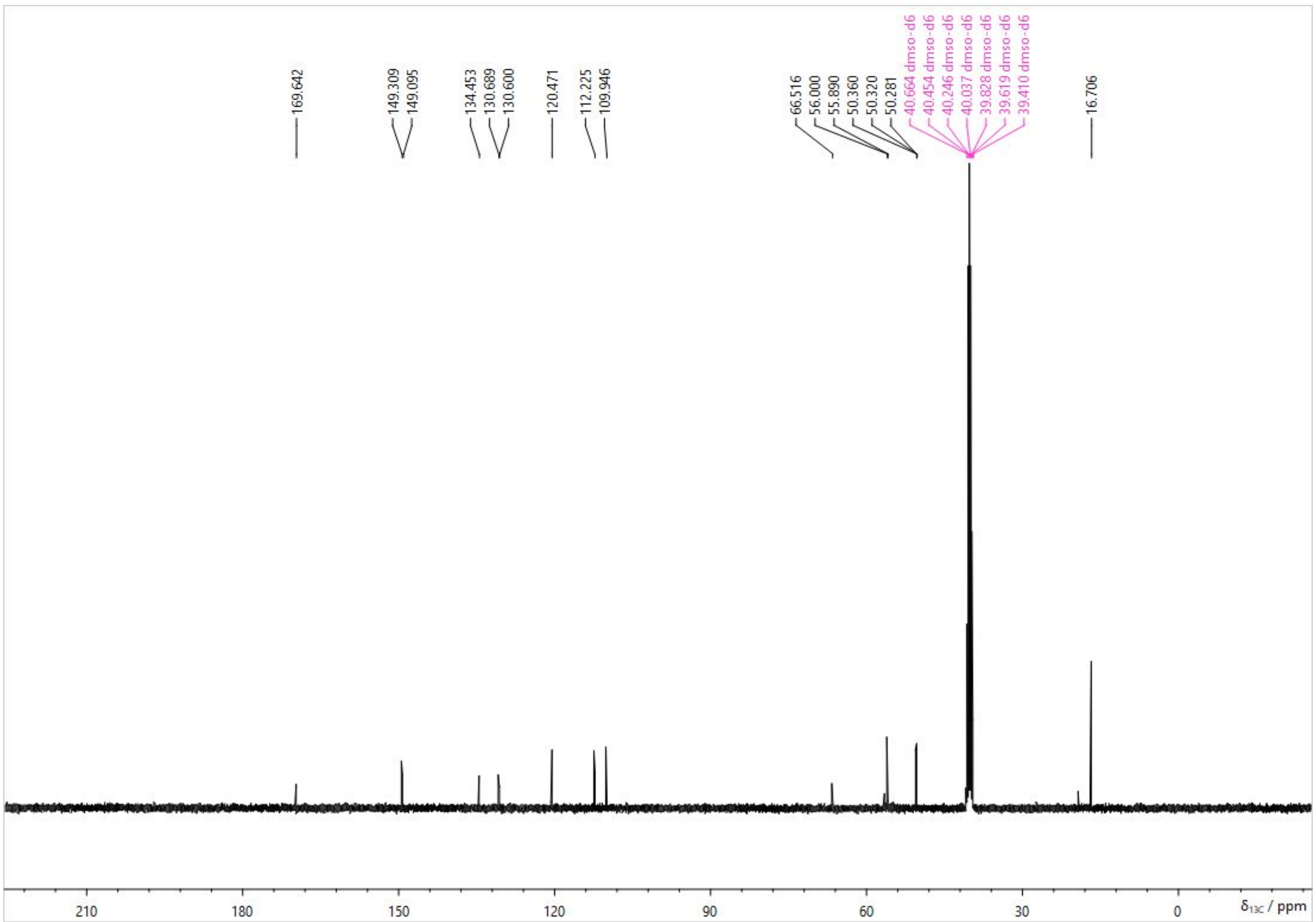

28 [TMC$_4$A][3,4-DCOAc]

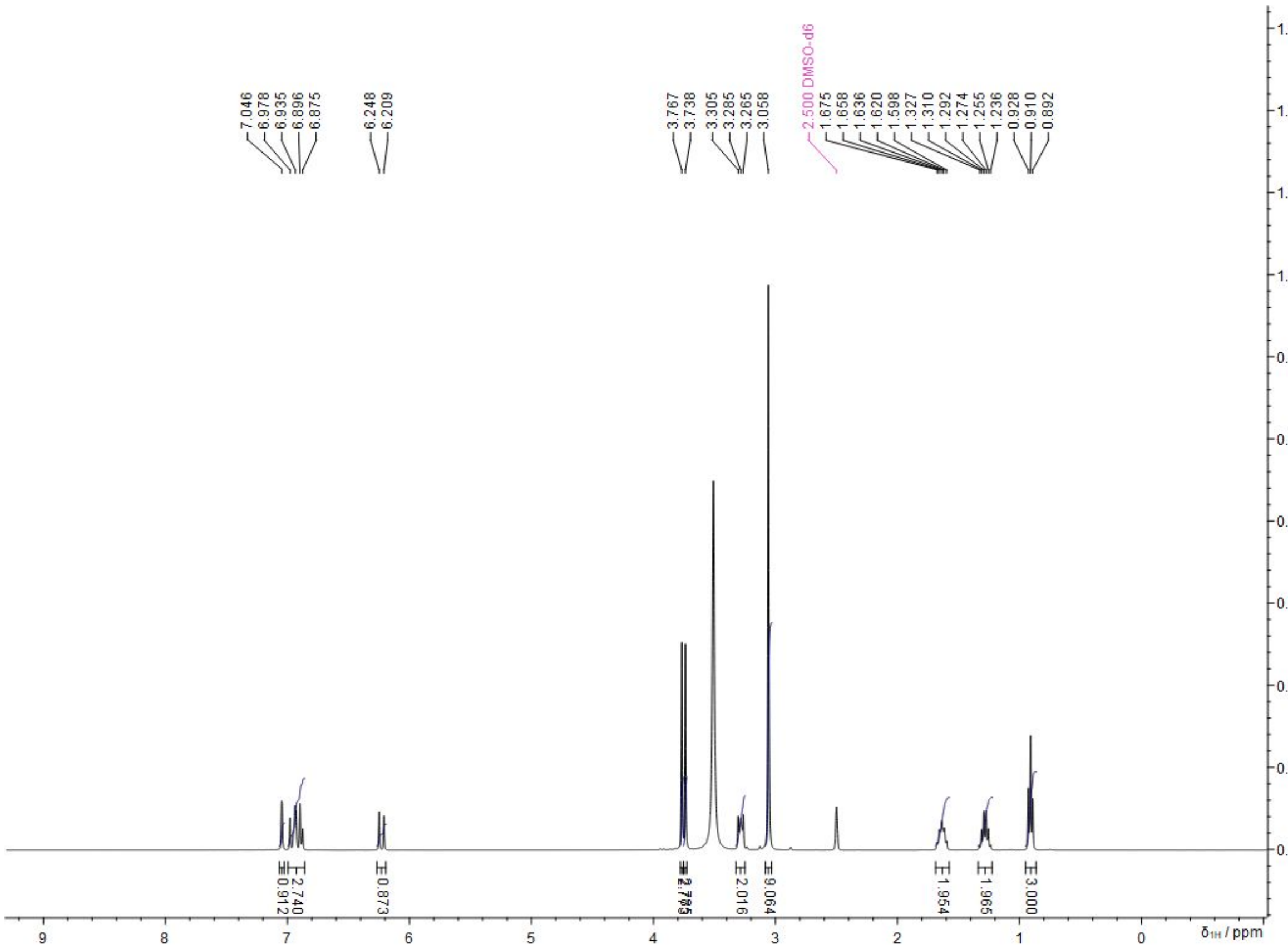

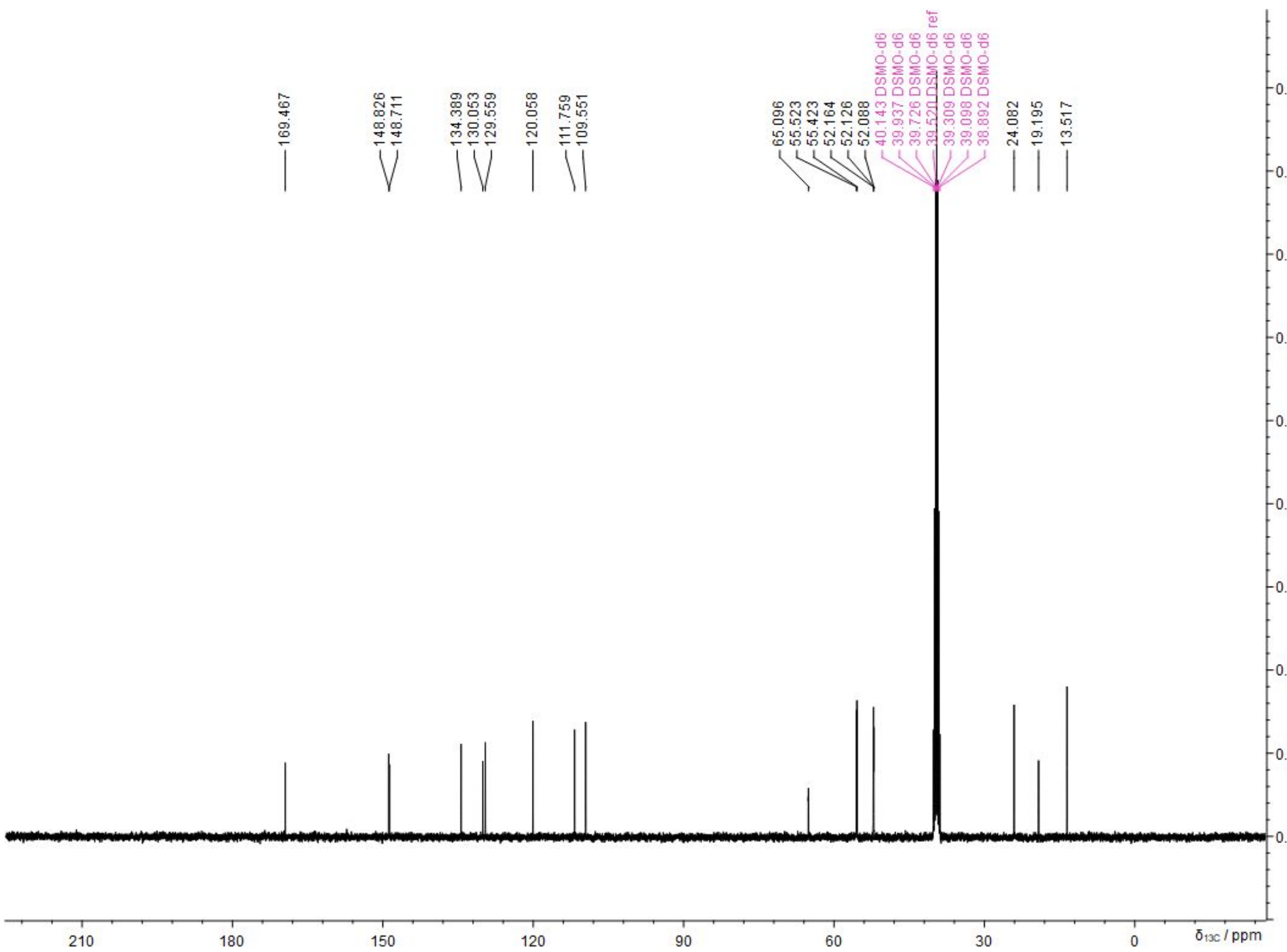

29 [TMC$_8$A][3,4-DCOAc]

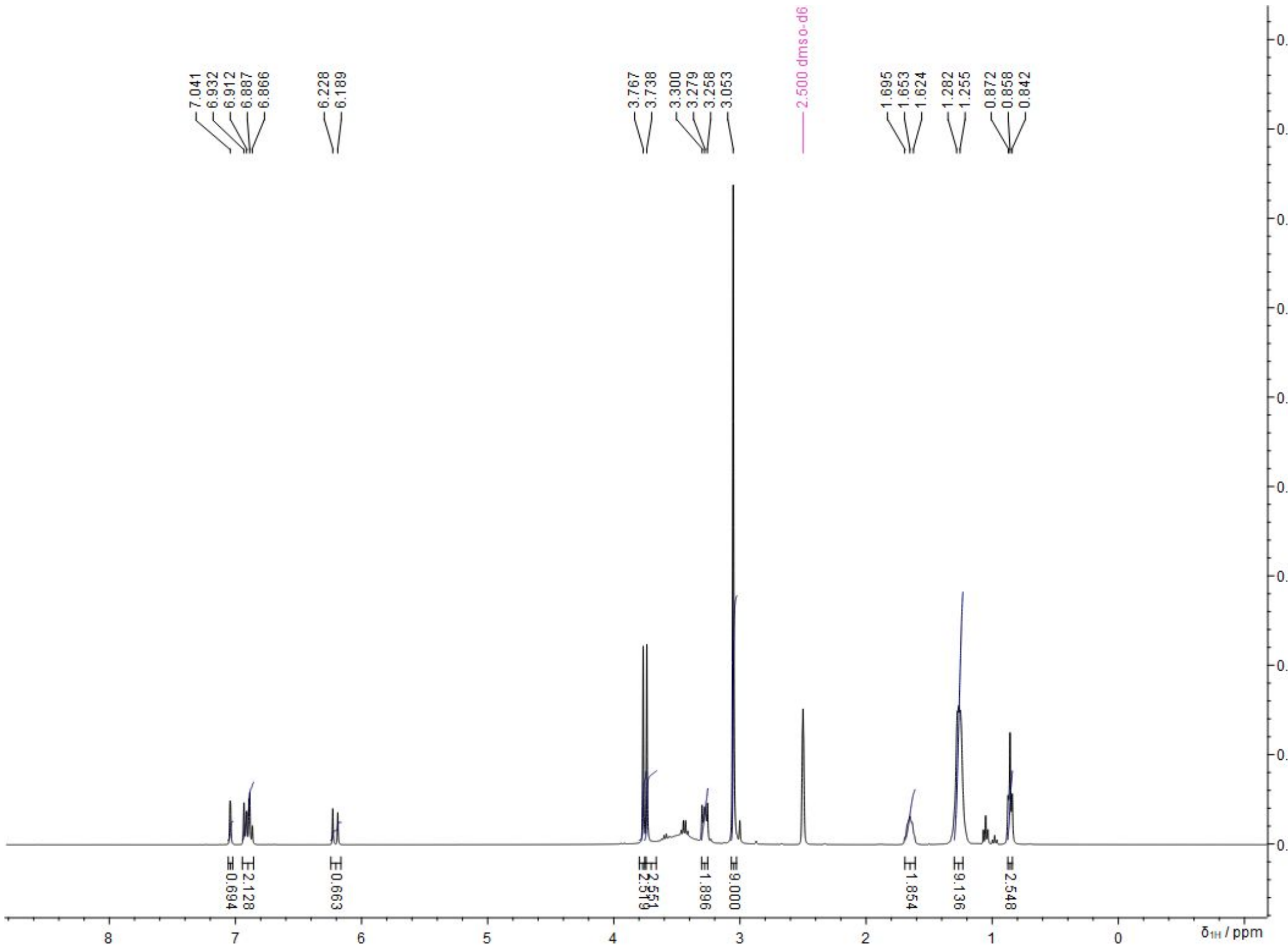

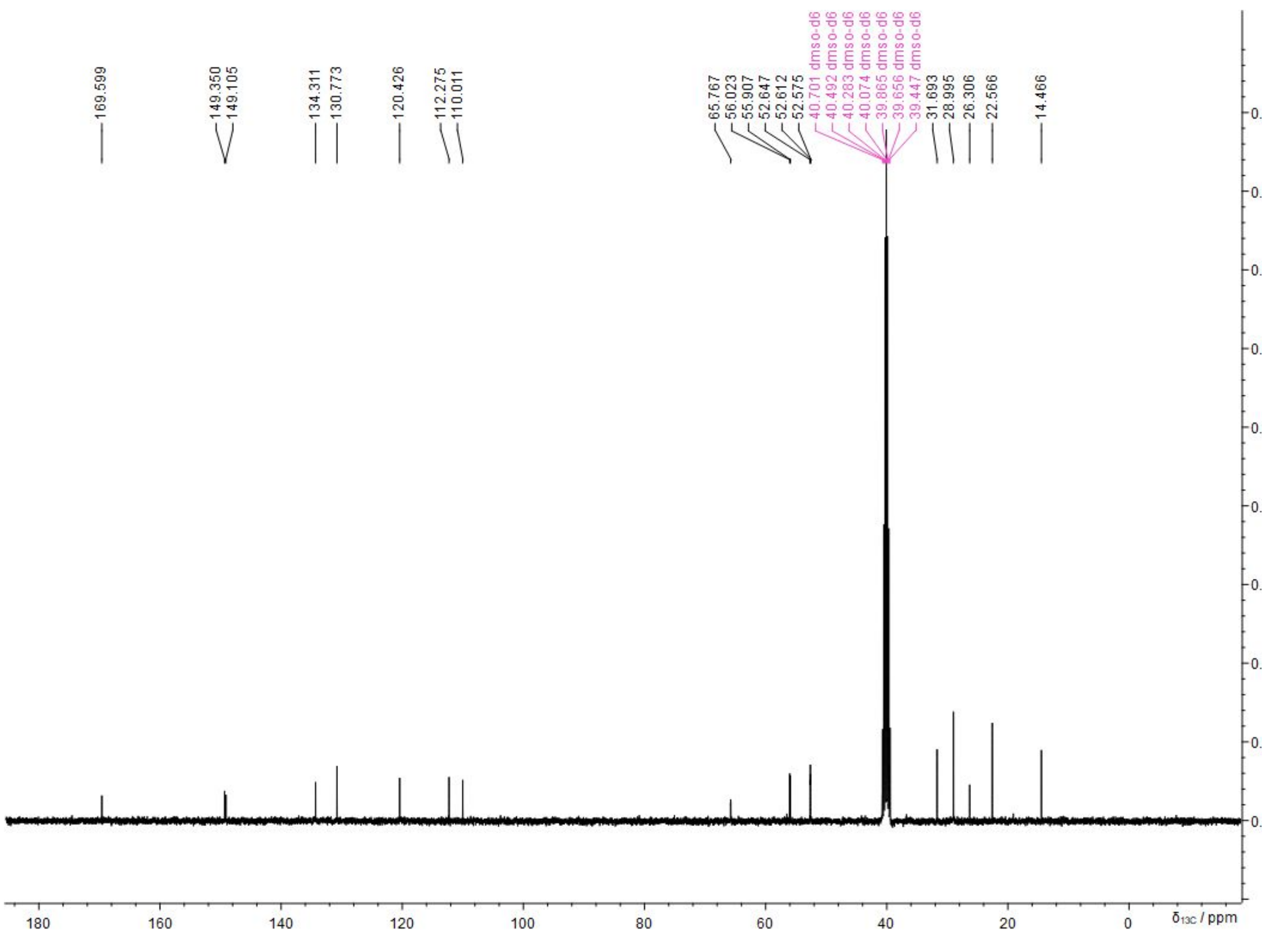

30 [$TMC_{16}A$][3,4-DCOAc]

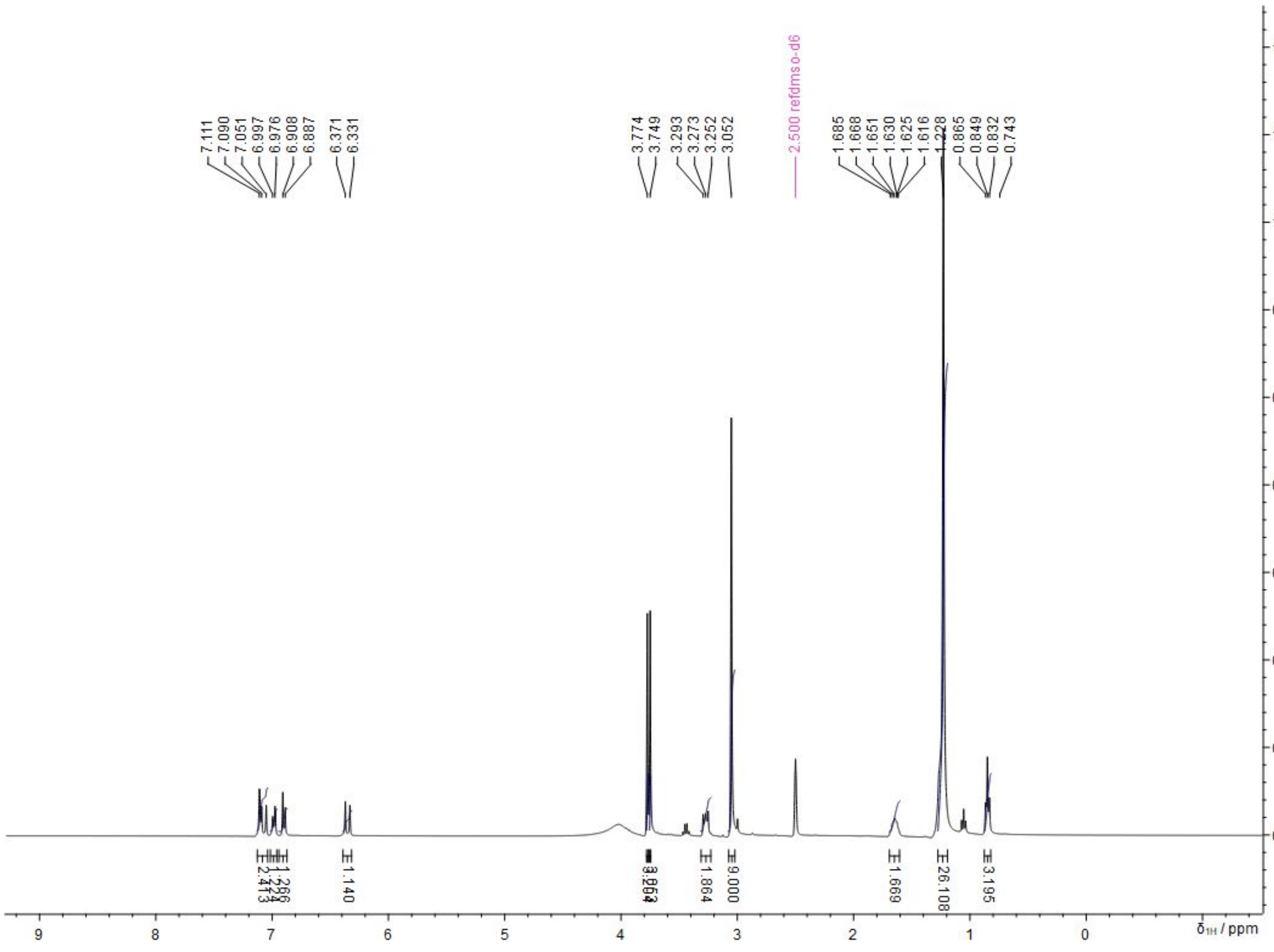

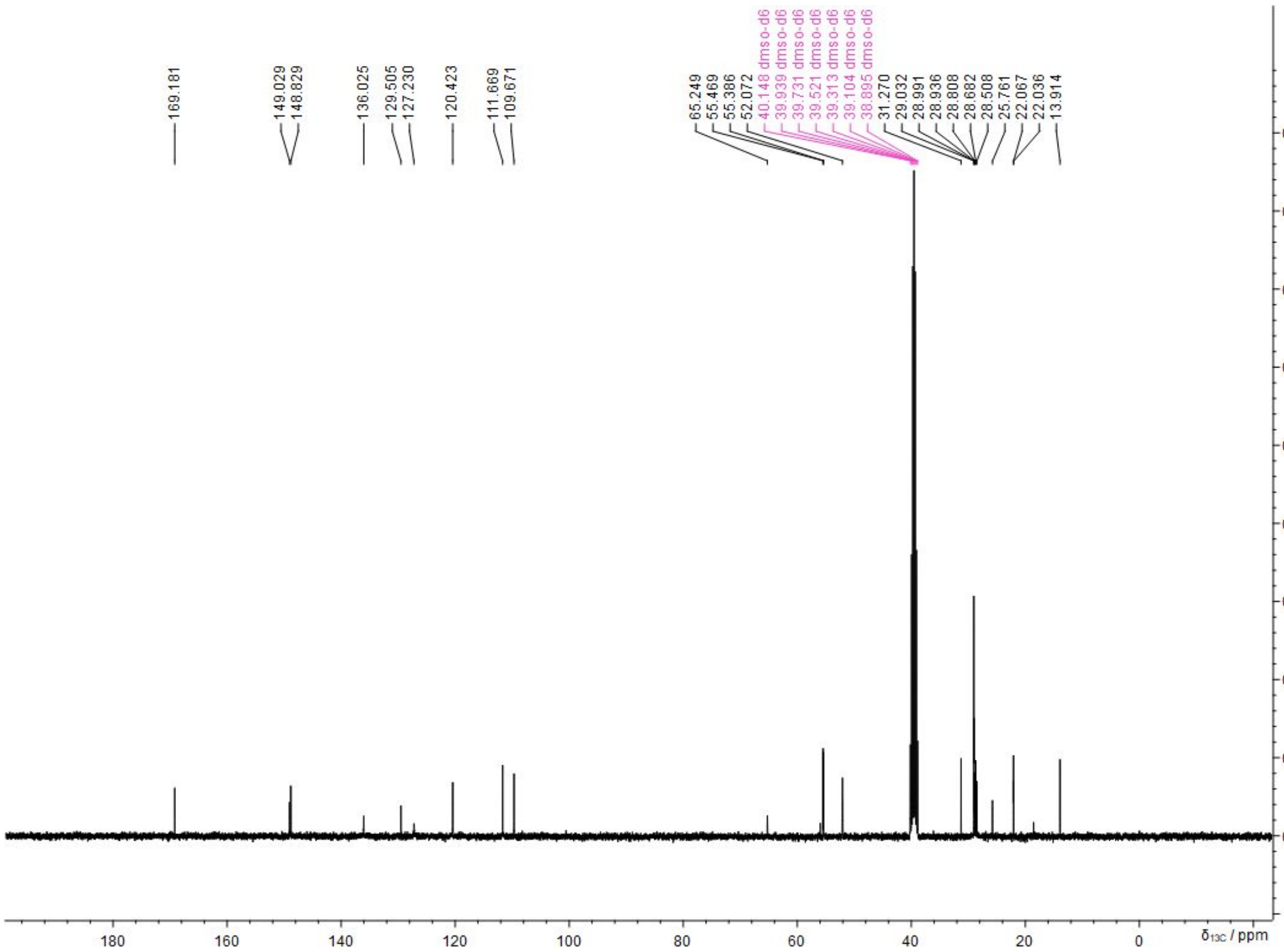

31 [TC$_4$MA][3,4-DCOAc]

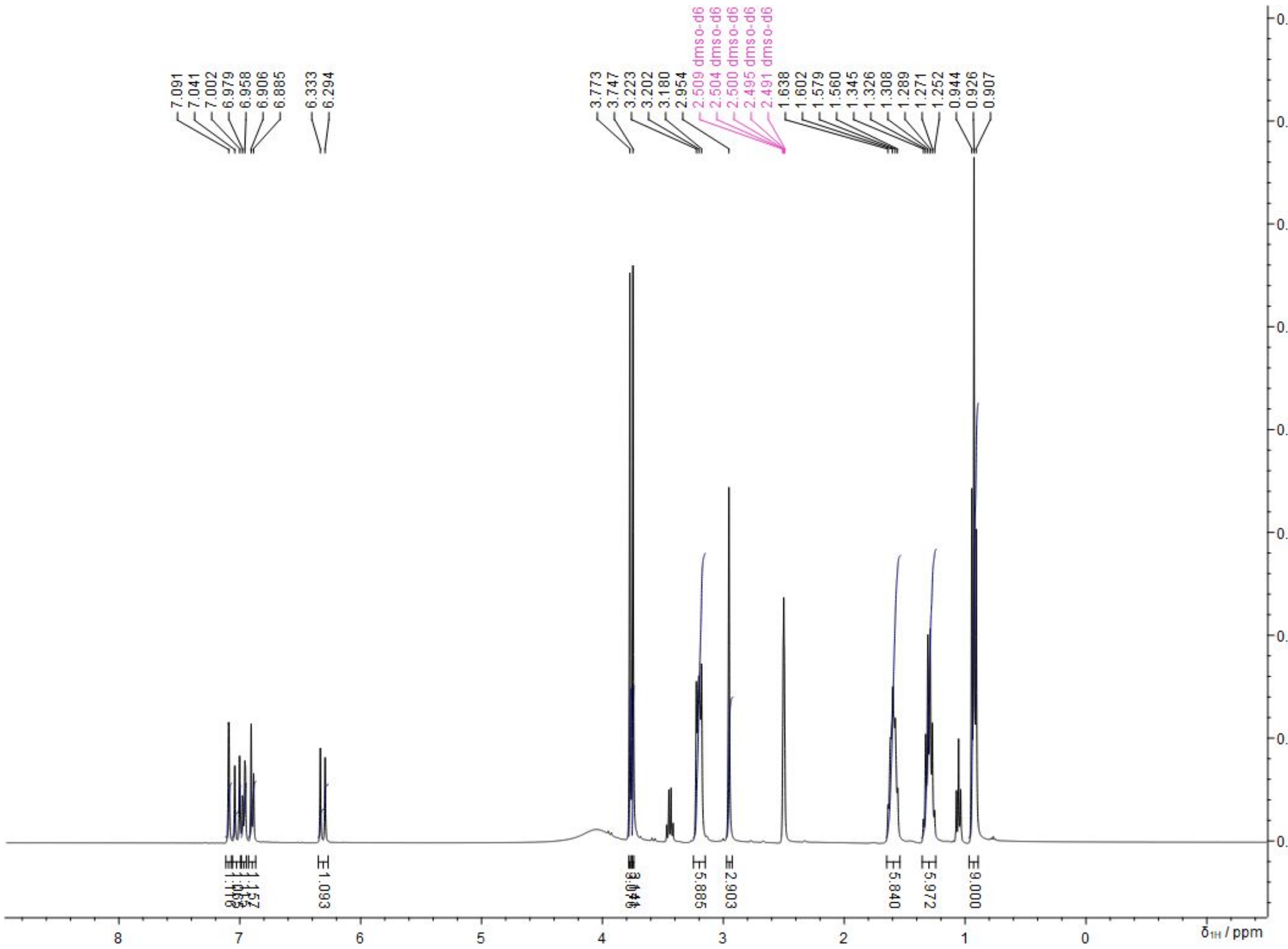

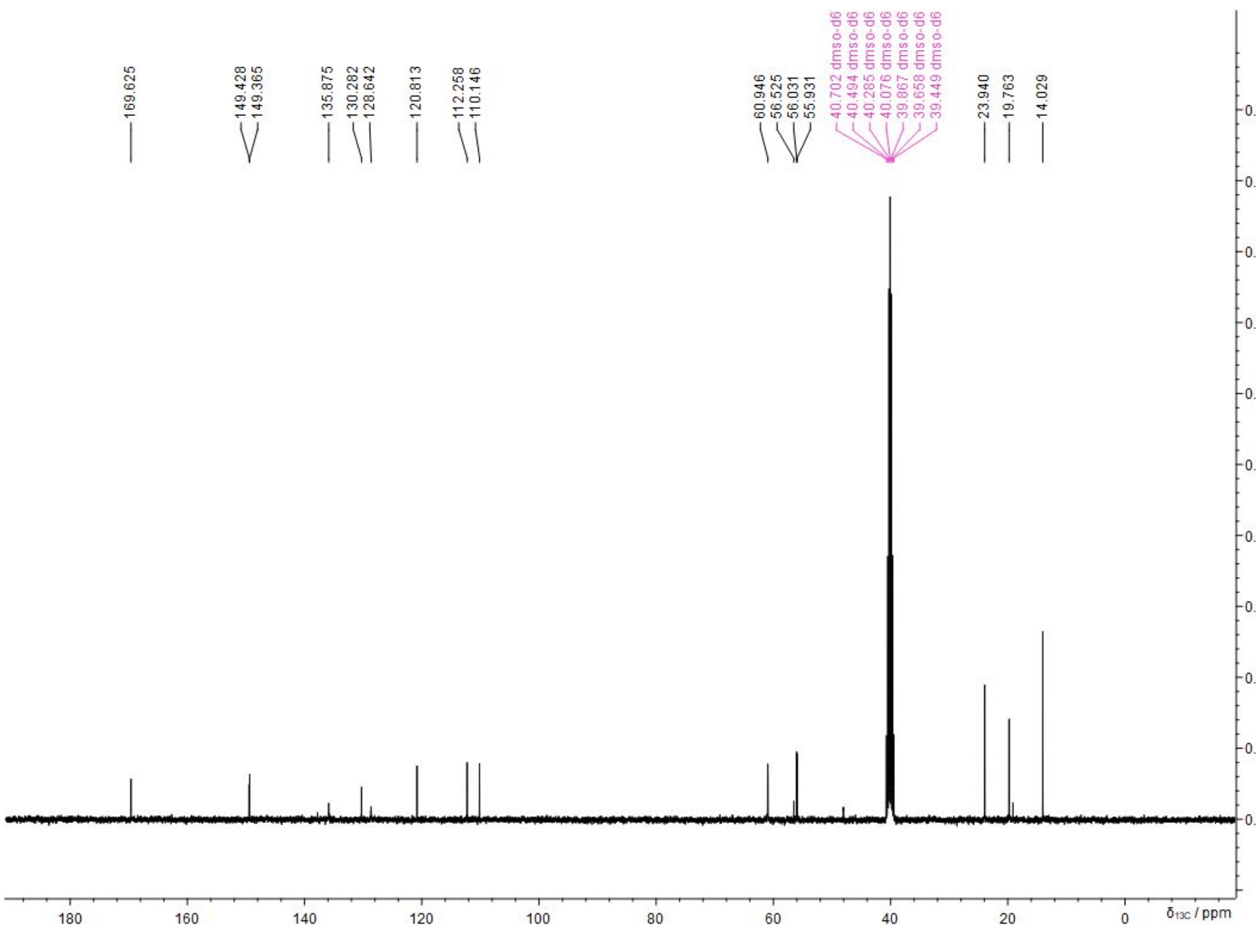

32 [TC_8MA][3,4-DCOAc]

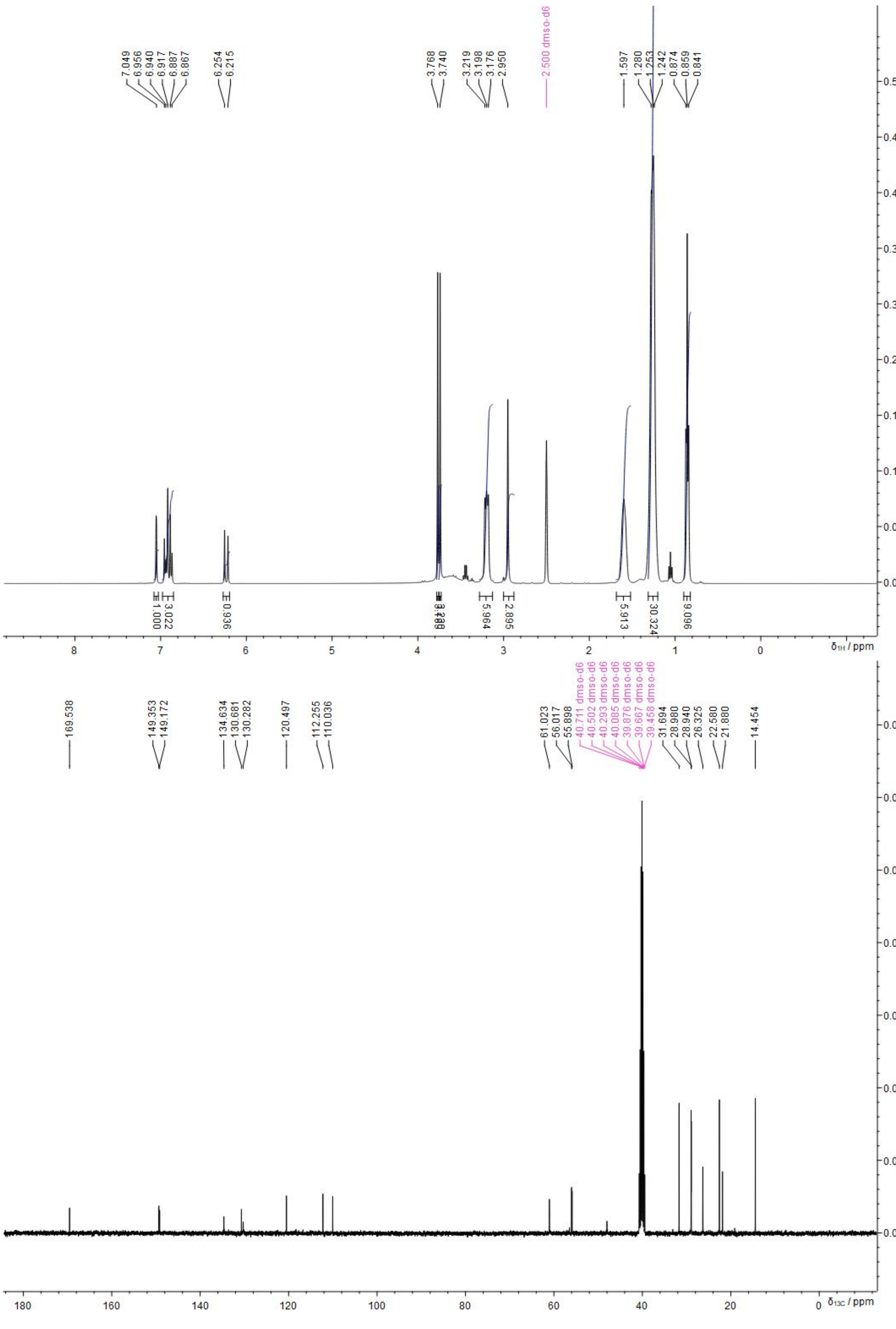

Supplement: Supplementary file 1 [file ao6c05301_si_001.pdf]
